# Routine Pediatric Enterovirus 71 Vaccination in China: a Cost-Effectiveness Analysis

**DOI:** 10.1371/journal.pmed.1001975

**Published:** 2016-03-15

**Authors:** Joseph T. Wu, Mark Jit, Yaming Zheng, Kathy Leung, Weijia Xing, Juan Yang, Qiaohong Liao, Benjamin J. Cowling, Bingyi Yang, Eric H. Y. Lau, Saki Takahashi, Jeremy J. Farrar, Bryan T. Grenfell, Gabriel M. Leung, Hongjie Yu

**Affiliations:** 1 WHO Collaborating Centre for Infectious Disease Epidemiology and Control, School of Public Health, Li Ka Shing Faculty of Medicine, The University of Hong Kong, Hong Kong Special Administrative Region, China; 2 Modelling and Economics Unit, Public Health England, London, United Kingdom; 3 Department of Infectious Disease Epidemiology, London School of Hygiene &Tropical Medicine, London, United Kingdom; 4 Division of Infectious Disease, Key Laboratory of Surveillance and Early Warning on Infectious Disease, Chinese Center for Disease Control and Prevention, Beijing, China; 5 Department of Ecology and Evolutionary Biology, Princeton University, Princeton, New Jersey, United States of America; 6 Oxford University Clinical Research Unit, Hospital for Tropical Diseases, Ho Chi Minh City, Viet Nam; 7 Fogarty International Center, National Institutes of Health, Bethesda, Maryland, United States of America; George Washington University, UNITED STATES

## Abstract

**Background:**

China accounted for 87% (9.8 million/11.3 million) of all hand, foot, and mouth disease (HFMD) cases reported to WHO during 2010–2014. Enterovirus 71 (EV71) is responsible for most of the severe HFMD cases. Three EV71 vaccines recently demonstrated good efficacy in children aged 6–71 mo. Here we assessed the cost-effectiveness of routine pediatric EV71 vaccination in China.

**Methods and Findings:**

We characterized the economic and health burden of EV71-associated HFMD (EV71-HFMD) in China using (i) the national surveillance database, (ii) virological surveillance records from all provinces, and (iii) a caregiver survey on the household costs and health utility loss for 1,787 laboratory-confirmed pediatric cases. Using a static model parameterized with these data, we estimated the effective vaccine cost (EVC, defined as cost/efficacy or simply the cost of a 100% efficacious vaccine) below which routine pediatric vaccination would be considered cost-effective. We performed the base-case analysis from the societal perspective with a willingness-to-pay threshold of one times the gross domestic product per capita (GDPpc) and an annual discount rate of 3%. We performed uncertainty analysis by (i) accounting for the uncertainty in the risk of EV71-HFMD due to missing laboratory data in the national database, (ii) excluding productivity loss of parents and caregivers, (iii) increasing the willingness-to-pay threshold to three times GDPpc, (iv) increasing the discount rate to 6%, and (v) accounting for the proportion of EV71-HFMD cases not registered by national surveillance. In each of these scenarios, we performed probabilistic sensitivity analysis to account for parametric uncertainty in our estimates of the risk of EV71-HFMD and the expected costs and health utility loss due to EV71-HFMD. Routine pediatric EV71 vaccination would be cost-saving if the all-inclusive EVC is below US$10.6 (95% CI US$9.7–US$11.5) and would remain cost-effective if EVC is below US$17.9 (95% CI US$16.9–US$18.8) in the base case, but these ceilings could be up to 66% higher if all the test-negative cases with missing laboratory data are EV71-HFMD. The EVC ceiling is (i) 10%–14% lower if productivity loss of parents/caregivers is excluded, (ii) 58%–84% higher if the willingness-to-pay threshold is increased to three times GDPpc, (iii) 14%–19% lower if the discount rate is increased to 6%, and (iv) 36% (95% CI 23%–50%) higher if the proportion of EV71-HFMD registered by national surveillance is the same as that observed in the three EV71 vaccine phase III trials. The validity of our results relies on the following assumptions: (i) self-reported hospital charges are a good proxy for the opportunity cost of care, (ii) the cost and health utility loss estimates based on laboratory-confirmed EV71-HFMD cases are representative of all EV71-HFMD cases, and (iii) the long-term average risk of EV71-HFMD in the future is similar to that registered by national surveillance during 2010–2013.

**Conclusions:**

Compared to no vaccination, routine pediatric EV71 vaccination would be very cost-effective in China if the cost of immunization (including all logistical, procurement, and administration costs needed to confer 5 y of vaccine protection) is below US$12.0–US$18.3, depending on the choice of vaccine among the three candidates. Given that the annual number of births in China has been around 16 million in recent years, the annual costs for routine pediatric EV71 vaccination at this cost range should not exceed US$192–US$293 million. Our results can be used to determine the optimal vaccine when the prices of the three vaccines are known.

## Abstract 2

Using surveillance and survey data, Joseph T. Wu and colleagues assess the cost-effectiveness of routine pediatric EV71 vaccination in China.

## Editors' Summary

**Background:**

Since 2007, outbreaks of hand, foot, and mouth disease (HFMD)—a contagious infection that mainly affects young children—have been occurring annually in China. Between 2010 and 2014, China accounted for 9.8 million of the 11.3 million cases of HFMD reported to the World Health Organization (WHO); in 2012, HFMD was the leading notifiable disease in China among children under five years old. HFMD is caused by a group of viruses called enteroviruses that are transmitted through contact with the mucus produced when an infected individual coughs or sneezes, through contact with the feces of an infected person, and through contact with contaminated surfaces. Good hygiene and frequent handwashing can reduce the spread of HFMD. The characteristic symptoms of HFMD are a non-itchy red rash with blisters on the hands and feet and painful mouth ulcers. There is no cure for HFMD, and most infected children get better within 7–10 days. However, some individuals develop potentially fatal complications such as encephalitis (infection and inflammation of the brain).

**Why Was This Study Done?:**

In China, enterovirus 71 (EV71) causes most laboratory-confirmed fatal cases of HFMD. Routine vaccination against EV71 during the first few months of life might therefore be one way to reduce China’s HFMD burden. In clinical trials, three inactivated monovalent EV71 vaccines made in China were shown to be safe and highly efficacious against EV71-associated HFMD (inactivated monovalent vaccines contain a single virus strain that cannot replicate; exposure to the vaccine “primes” the immune system to respond quickly when challenged with live virus, thereby preventing infection with that virus). However, before implementing routine EV71 vaccination, it is important to know whether this intervention is a good value for the money it would cost. For example, how much money needs to be spent on vaccination to save one life? In this cost-effectiveness analysis (a study that estimates the costs and health effects of a medical intervention), the researchers assess the value for money of routine vaccination of young children against EV71 in China.

**What Did the Researchers Do and Find?:**

The researchers characterized the health and economic burden of EV71-associated HFMD in China using the national surveillance database, HFMD laboratory test results, and information on household costs and health utility loss associated with HFMD cases (health utility is a number that is assigned to a state of health; perfect health and death have utility values of 1 and 0, respectively) collected in a caregiver survey. They then used a mathematical model to estimate the effective vaccine cost (EVC; vaccine cost divided by efficacy) below which routine pediatric vaccination would be cost-effective; WHO defines a cost-effective intervention as one in which the incremental cost-effectiveness ratio (the incremental costs of introducing an intervention divided by the incremental benefits accrued by that introduction) is between one and three times the country’s gross domestic product (GDP) per capita. Routine pediatric vaccination was cost-effective in the researchers’ base-case analysis—which assumed a willingness-to-pay threshold of one times GDP per capita—if the EVC was below US$17.9. Increasing the willingness-to-pay threshold to three times GDP per capita increased the EVC below which routine vaccination would be cost-effective by 58%–84%, whereas excluding consideration of the productivity loss of parents/caregivers while caring for a child with HFMD reduced the EVC below which routine vaccination would be cost-effective by 10%–14%.

**What Do These Findings Mean?:**

The validity of these findings depends on the assumptions included in the mathematical model and on the accuracy of the data fed into the model. However, routine pediatric EV71 vaccination remained cost-effective at broadly similar EVCs in sensitivity analyses in which the assumptions built into the model were altered. Overall, these findings suggest that routine pediatric EV71 vaccination would be very cost-effective in China provided the cost of immunization (including the cost of the vaccine and all the logistical and administration costs of vaccination) is below between US$12.0 and US$18.3 per vaccination; because the different vaccines have different efficacies, the exact value depends on which vaccine is used for vaccination. Thus, with 16 million births each year, the annual costs for routine pediatric EV71 vaccination in China should not exceed US$192–US$293 million. Importantly, when combined with the findings of a previous study in which the same researchers showed large geographical variations in the risk of EV71-associated HFMD across China, these findings can help policymakers identify those regions in China where EV71 vaccination is likely to be most cost-effective.

**Additional Information:**

This list of resources contains links that can be accessed when viewing the PDF on a device or via the online version of the article at http://dx.doi.org/10.1371/journal.pmed.1001975.

- A *PLOS Medicine* Research Article by Takahashi et al. provides information about the patterns of HFMD outbreaks in China
- The US Centers for Disease Control and Prevention provides information on hand, foot, and mouth disease (in English and Spanish), including a podcast on the condition
- The UK National Health Service Choices website provides detailed information on hand, foot, and mouth disease
- Further information about hand, foot, and mouth disease is provided by the World Health Organization (including up-to-date HFMD surveillance reports from China), the Nemours Foundation (in English and Spanish), and MedlinePlus (in English and Spanish)
- A WHO guide on cost-effectiveness analysis is available

## Introduction

Since the 1990s, large epidemics of hand, foot, and mouth disease (HFMD) have occurred across the Western Pacific region [1–6]. In China, which accounted for 87% (9.8 million/11.3 million) of all HFMD cases reported to WHO during 2010–2014 [7], HFMD epidemics have been occurring annually since 2007. Between 2008 and 2013, China’s national HFMD surveillance registered around 9 million cases and 2,700 deaths, 90% and 96% of which, respectively, occurred in children under 5 y [6]. In 2012, HFMD ranked first among all notifiable diseases in China by both case count and deaths for children under 5 y [8].

Enterovirus 71 (EV71) is a major causative pathogen of HFMD epidemics. In China, EV71 accounted for more than 90% of laboratory-confirmed fatal HFMD cases between 2008 and 2013 [6]. EV71-associated HFMD (EV71-HFMD) causes substantial morbidity and mortality because effective therapeutic and preventive measures remain elusive. There is no established antiviral treatment for EV71-HFMD [9]. The prevention measures suggested by WHO are hand hygiene and social distancing, the efficacy of which are uncertain [10,11].

Three inactivated monovalent EV71 vaccines (each requiring two doses administered 4 wk apart) manufactured in China were recently shown in phase III trials to be safe and efficacious against EV71-HFMD for children aged 6–71 mo [12–14]. Economic evaluation is important in considering population rollout of any new vaccine [15]. Here we provide such an evaluation using the best available data comprising (i) national HFMD surveillance, (ii) HFMD virological surveillance records from all 31 provinces, and (iii) a nationwide caregiver survey on the economic costs and health utility loss associated with EV71-HFMD.

## Methods

### Ethics Statement

Ethical approval (approval number 201417) was acquired from the institutional review board of the Chinese Center for Disease Control and Prevention. Caregiver survey interviews were conducted only after verbal informed consent was obtained from a parent or caregiver of the pediatric patient. In May 2008, HFMD was added to the list of notifiable diseases in China. According to China’s law on the prevention and treatment of infectious diseases, personal identifiers should be collected for individual cases with diagnosis of a notifiable disease, for the purposes of public health surveillance and response. The National Health and Family Planning Commission of China decided that the collection of individual data for all notifiable diseases, including HFMD, according to the national surveillance protocol was part of an ongoing public health response and was thus exempt from institutional review board assessment.

### Model

We used a static model to perform a cost-effectiveness analysis (CEA) of routine pediatric EV71 vaccination in China (i.e., a model with no indirect protection). In a separate study, we used a time series susceptible–infected–recovered (TSIR) dynamic model to show that transmissibility of EV71-HFMD in China is high (with a national average basic reproductive number of around 27) and that indirect protection from EV71 vaccination is unlikely to be significant [16]. Herd immunity appears only at very high vaccine coverage levels and hence can be ignored at the coverage levels considered in our analysis. As such, we have chosen to use a static model for the CEA because it provides a simple yet very accurate model of the reduction in EV71-HFMD incidence conferred by routine pediatric EV71 vaccination. S1 Fig shows that the long-term incidence reduction predicted by the static model is essentially the same as that predicted by the TSIR dynamic model. See “Indirect Protection Conferred by Vaccination” in S1 Text for a more detailed explanation of this phenomenon.

**Fig 1:**
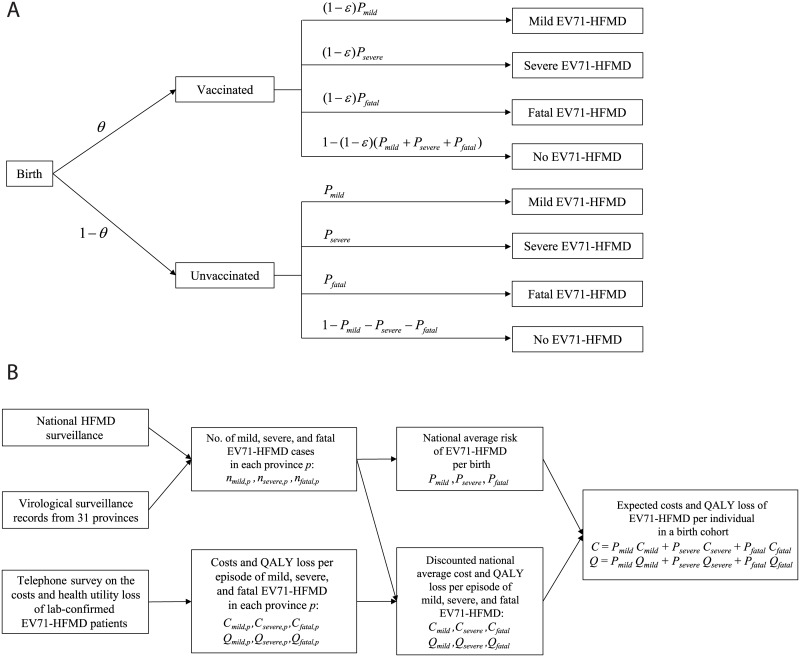
Model structure and parameterization. (A) The static model used in the CEA with vaccine coverage θ and vaccine efficacy ε. (B) A schematic that describes how the data sources were used to parameterize the model. See S1 Text for more details. QALY, quality-adjusted life year.

Fig 1 summarizes the model structure and parameterization, with further details provided in “The Model” in S1 Text. We considered only children aged 6 mo to 5 y (60 mo) because (i) 90%, 94%, and 96% of mild, severe, and fatal HFMD cases, respectively, occur in this age group [6] and (ii) vaccine efficacy has been demonstrated only in those aged 6–71 mo [12–14]. The life expectancy of each birth cohort was assumed to be 75 y [17].

We assumed that (i) children would be vaccinated 6 mo after birth, with immediate and constant efficacy ε against EV71-HFMD for at least 5 y, and (ii) conservatively, if vaccinated children developed EV71-HFMD, the probability of severe outcomes was the same as that for unvaccinated children. Consequently, the risk of EV71-HFMD in vaccinated children was reduced by a proportion ε for all levels of severity.

### Data

We parameterized the model using the following data.

#### National HFMD surveillance dataset during 2010–2013 (S1 Data)

China’s national HFMD surveillance system has been described in our previous report [6]. Briefly, the system classifies HFMD cases as severe if the patient experiences neurological, respiratory, or cardiopulmonary complications; otherwise, cases are classified as mild [6]. Virological surveillance requires all hospitals to collect specimens for serotyping from (i) all severe and fatal cases and (ii) the first five mild cases every month. Serotyping results were classified into four categories: EV71, Coxsackievirus A16 (CA16), other enterovirus (OEV), or test-negative for enteroviruses. The system, however, does not record test-negative results. While some test-negative cases could be misdiagnoses (e.g., chicken pox or measles), others could be *bona fide* EV71-HFMD cases but with negative test results because of late collection of specimens, poor specimen quality, or other reasons. To account for the uncertainty regarding the percentage of test-negative cases that were EV71-HFMD, we acquired internal laboratory records from all 31 provinces (below) to supplement our analysis.

#### Virological surveillance records from all 31 provinces (S1 Data)

**Fig 2:**
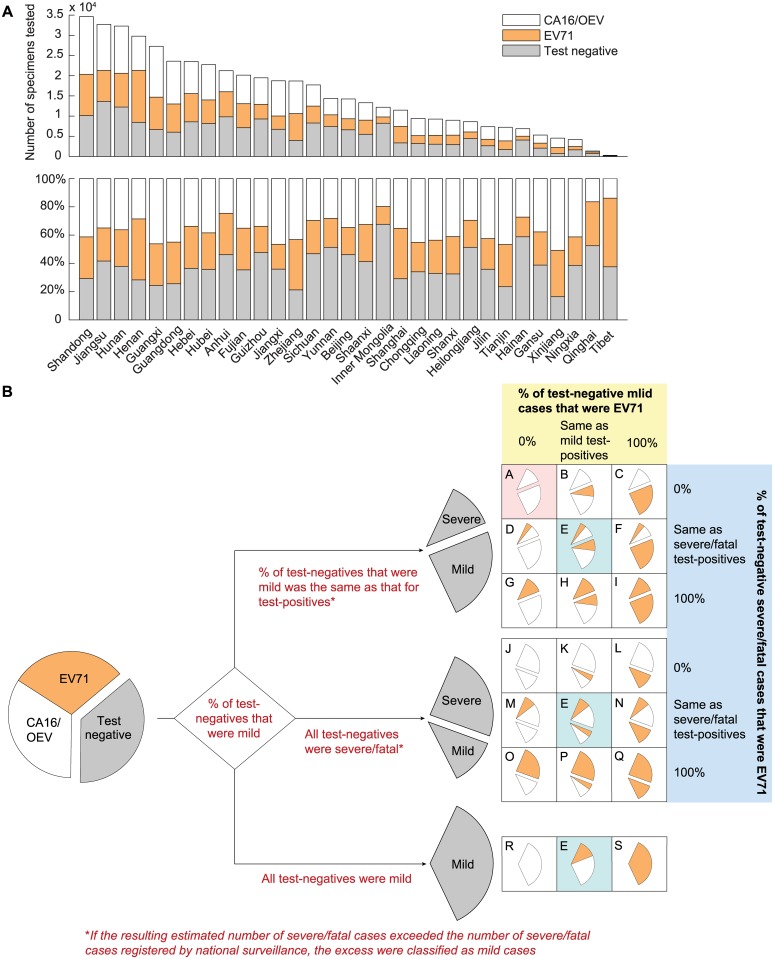
Estimating the percentage of mild and severe HFMD cases attributed to EV71 in China. (A) The number of specimens tested and the distribution of test results in each of the 31 provinces during 2010–2013. (B) Twenty-one scenarios were generated by making assumptions regarding the percentage of test-negative cases that were mild during 2010–2012 (the first branching point; annotated in red text) and the percentage of test-negative mild and severe/fatal cases that were EV71 during 2010–2013 (the second branching point; annotated in yellow and blue shades), respectively. The outcomes in the three scenarios colored in cyan were identical; hence, there were only 19 unique scenarios (labeled A–S). The base case (scenario A) is colored in pink. The sizes of the wedges illustrate the underlying assumptions and are not proportional to the actual percentages (the differences in serotype and severity distribution for severe/fatal cases between scenarios would not be apparent otherwise because the number of mild cases was much larger than that of severe/fatal cases).

This dataset contained the weekly number of EV71, CA16, OEV, and test-negative results in each province from 1 January 2010 to 31 December 2013 (Fig 2A). These records contained no severity information until 31 December 2012. As such, we considered 19 test-negative scenarios (scenarios A–S in Fig 2B) that corresponded to assumptions regarding (i) the percentage of test-negative cases that were mild, (ii) the percentage of test-negative mild cases that were EV71-HFMD, and (iii) the percentage of test-negative severe/fatal cases that were EV71-HFMD. As shown in Fig 2B, there were 21 original scenarios, but three of them were identical, hence only 19 unique scenarios. See “Uncertainty in Test-Negative Cases” in S1 Text for details.

#### Caregiver survey

A telephone survey was conducted of parents or caregivers of children aged 6 mo to 5 y with laboratory-confirmed HFMD diagnosed between 1 January 2012 and 17 December 2013 as registered by national surveillance (S2 and S3 Data). Information about the child’s diagnosis and treatment and household expenses, time off work, and quality of life related to the HFMD episode was obtained from respondents who consented to be interviewed. Health-related quality of life was measured using the EuroQol EQ-5D-3L instrument (http://www.euroqol.org/). Briefly, we contacted 29,810 parents or caregivers of HFMD patients, and 3,500 agreed to participate in our survey. Of these, 1,787 were parents or caregivers of EV71-HFMD patients. The mean total cost of a pediatric EV71-HFMD episode reported in China was US$233, US$1,173, US$3,279, and US$2,738 for mild outpatient, mild inpatient, severe, and fatal cases, respectively. The mean quality-adjusted life year (QALY) loss per episode was 0.0039, 0.0070, 0.0194, and 30.4, respectively. We found little evidence of associations between costs or QALY loss and patients’ age, sex, or urban residence, so we stratified costs and QALY loss by case severity and geographical region only. We estimated the mean cost and QALY loss per episode using the central limit theorem and accounted for the associated parametric uncertainty in probabilistic sensitivity analysis. See “Survey on Household Costs and Quality of Life Detriment Associated with EV71-HFMD” in S1 Text for details.

### Burden of Disease

We assumed that without vaccination, the risk of EV71-HFMD at all levels of severity for future birth cohorts (denoted by *P*_mild,_ *P*_severe_, and *P*_fatal_) would be similar to that registered by national surveillance during 2010–2013. At each level of severity *s*, *P*_*s*_ comprised the risk of EV71-HFMD for the corresponding level of severity in each province (Fig 1). See “The Model” in S1 Text for mathematical details.

### Costs and Health Utility Loss Due to EV71-HFMD

The cost of EV71-HFMD per birth, *C*, was estimated as the weighted sum of the average cost of mild, severe, and fatal cases (*C*_mild,_ *C*_severe_, and *C*_fatal_), where the weights were simply the risk of mild, severe, and fatal EV71-HFMD (*P*_mild,_ *P*_severe_, and *P*_fatal_). The health utility loss attributable to EV71-HFMD, *Q*, was estimated analogously from the health utility loss of mild, severe, and fatal cases (*Q*_mild,_ *Q*_severe_, and *Q*_fatal_). Cost and health utility loss at each level of severity were discounted according to the age distribution of cases for the corresponding level of severity in each province. We did not account for adverse events following immunization because such events in the phase III trials were largely mild and uncommon [12–14]. We excluded productivity loss due to pediatric premature death because of unresolved debate about the way such losses should be costed [18]. See “The Model” in S1 Text for details.

### Cost-Effectiveness

The incremental cost-effectiveness ratio (ICER) of vaccine introduction was calculated as follows:

$$\begin{array}{ll} \text{ICER} & =\frac{\text{Cost with EV71 vaccination}-\text{Cost with no vaccination}}{\text{Health utility with EV71 vaccination}-\text{Health utility with no vaccination}}\\ & =\frac{\text{Vaccine cost}-\text{(Vaccine efficacy)}\times \text{(Cost due to EV71-HFMD per birth)}}{\text{(Vaccine efficacy)}\times \text{(Health utility lost due to EV71-HFMD per birth)}}\\ & =\frac{\text{(Vaccine cost)/(Vaccine efficacy)}-\text{(Cost due to EV71-HFMD per birth)}}{\text{Health utility lost due to EV71-HFMD per birth}}\\ & =\frac{\text{EVC}-C}{Q} \end{array}$$ (1)

where vaccine cost was the cost per child fully vaccinated, which included the procurement, logistical, and administration cost for the necessary number of vaccine doses to confer 5 y of protection. Because the three EV71 vaccines have significantly different efficacy estimates [12–14] and reliable estimates of their costs are not yet available, we used effective vaccine cost (EVC), defined as (vaccine cost)/(vaccine efficacy), which is simply the cost adjusted for efficacy, or the cost of a 100% efficacious vaccine, as the outcome for our CEA. Given a willingness-to-pay threshold, routine pediatric EV71 vaccination would be cost-effective if and only if

$$\begin{array}{l} \text{EVC < (Willingness-to-pay threshold)}\times \text{(Health utility loss due to EV71-HFMD per birth)}\\ \text{+ Cost due to EV71-HFMD per birth} \end{array}$$ (2)

We denote this ceiling by EVC_max_ hereafter. With EVC_max_ as the outcome of our CEA, we can easily obtain the cost-effective vaccine cost ceiling of any given vaccine by multiplying EVC_max_ by the corresponding vaccine efficacy.

### Base Case

We performed the CEA from the societal perspective [19]. Costs and health utility loss were discounted at 3% per annum [20]. We used one times the gross domestic product per capita (GDPpc) (US$6,700 in 2013 for China [21]), which is the WHO-CHOICE criterion for very cost-effective interventions [20], as the societal willingness-to-pay threshold. In terms of assumptions regarding test-negative cases, we used the most conservative assumptions in the base case (colored in pink in Fig 2B) in order to avoid overestimating the cost-effectiveness of vaccination. This corresponded to assuming that (i) the percentage of test-negative cases that were mild was the same as that for test-positive cases (at the first branching point in Fig 2B) and (ii) none of the test-negative cases were EV71 (at the second branching point in Fig 2B).

### Uncertainty Analysis

We considered all possible combinations of the following decision-making uncertainty associated with the choices of CEA parameters: (i) inclusion or exclusion of productivity loss of parents or caregivers due to caring for their sick children (i.e., indirect non-medical costs), (ii) discounting costs and health utility loss at 3% or 6%, and (iii) using one or three times GDPpc as the willingness-to-pay threshold (the latter is the WHO-CHOICE criterion for cost-effective interventions [20]). For each combination of these possibilities (no probability distributions are imposed on these parameters), we calculated EVC_max_ for all 19 test-negative scenarios in Fig 2B.

In addition, we considered the uncertainty associated with under-ascertainment and underreporting [22]. The validity of our CEA substantially depends on the proportion of EV71-HFMD cases not registered by national surveillance. To estimate this proportion, we compared the incidence rate of EV71-HFMD reported in the EV71 vaccine phase III trials (in which intensive active surveillance for HFMD was conducted in one county of Beijing, six counties of Jiangsu, and seven counties of Guangxi) with the incidence rate of EV71-HFMD registered by national surveillance in the counties and time periods of these trials [12–14]. We estimated the EV71-HFMD incidence rate in each trial using the number of participants and EV71-associated diseases in the placebo group. See “Estimation of the Unregistered Proportion” in S1 Text for details. We then repeated the calculation of EVC_max_ in all of the above-mentioned scenarios assuming that the resulting unregistered proportion was generalizable to the whole country and that the expected cost and QALY loss of registered and unregistered cases were similar.

### Probabilistic Sensitivity Analysis

In each scenario, we performed probabilistic sensitivity analysis to account for parametric uncertainty in our estimates of the risk of EV71-HFMD (*P*_mild,_ *P*_severe_, *P*_fatal_) and the expected costs and QALY loss per case due to EV71-HFMD (*C*_mild,_ *C*_severe_, *C*_fatal_, *Q*_mild,_, *Q*_severe_, *Q*_fatal_; see “Survey on Household Costs and Quality of Life Detriment Associated with EV71-HFMD” in S1 Text for details). When considering the unregistered proportion in the uncertainty analysis, we assumed the proportion of EV71-HFMD cases registered by national surveillance followed a beta distribution that corresponded to the mean and 95% CI obtained from the comparison of incidence rate between the EV71 vaccine phase III trials and the national surveillance database. See Table 1 for details on the probability distributions used in the probabilistic sensitivity analysis.

**Table 1:** Parameters for the cost-effectiveness model.

Description	Baseline Value	Values for Uncertainty Analysis	Values for Probabilistic Sensitivity Analysis	Source
**Risk of EV71-HFMD (per 100,000 births)**			Dirichlet distribution with parameters equal to one plus the number of mild, severe, and fatal cases	National HFMD surveillance database and virological surveillance records from 31 provinces during 2010–2013. Values for uncertainty analysis corresponded to the risk estimates across the 19 scenarios in Fig 2B. Values for probabilistic sensitivity analysis corresponded to the conjugate posterior distributions for multinomial likelihood assuming that the priors were a non-informative Dirichlet distribution (all parameters equal to one).
Mild, *P*_mild_	3,088	2,932–7,077		
Severe, *P*_severe_	83	75–107		
Fatal, *P*_fatal_	3.13	3.04–3.53		
**Expected cost due to EV71-HFMD per birth including productivity loss of parents/caregivers**			Expected cost and QALY loss in each severity–region stratum followed bivariate normal distribution with mean and covariance matrix shown in S12 Table	Telephone survey of parents/caregivers of 1,787 laboratory-confirmed EV71-HFMD pediatric cases. Expected cost and QALY loss in each severity–region stratum were estimated using the central limit theorem.
Mild, *C*_mild_	7.86	—		
Severe, *C*_severe_	2.68	—		
Fatal, *C*_fatal_	0.08	—		
**Expected cost due to EV71-HFMD per birth excluding productivity loss of parents/caregivers**				
Mild, *C*_mild_	6.11	—		
Severe, *C*_severe_	2.57	—		
Fatal, *C*_fatal_	0.07	—		
**Expected QALY loss due to EV71-HFMD per birth**				
Mild, *Q*_mild_	11.2 × 10^−5^	—		
Severe, *Q*_severe_	1.24 × 10^−5^	—		
Fatal, *Q*_fatal_	95.3 × 10^−5^	—		
**Discount rate for cost and health utility**	3%	3% or 6%	—	Assumed
**Willingness-to-pay threshold**	1 × GDPpc	1 or 3 × GDPpc	—	Assumed
**Proportion of EV71-HFMD cases registered by national surveillance**	1	1 or 0.74 with parameter uncertainty	Beta distribution with mean 0.74 and standard deviation 0.053	Comparison of the incidence rate of EV71-HFMD in the three vaccine trials with that in the national surveillance

### Comparative Cost-Effectiveness Analysis of the Three Vaccines

The three EV71 vaccines have different efficacy estimates, and reliable estimates of their costs are not yet available. As such, we used EVC_max_ to identify cost regions where each vaccine would be preferred given uncertainty in their efficacies. Specifically, because EVC is simply the cost per unit of vaccine efficacy purchased, it could be used to compare the incremental cost-effectiveness of any two vaccines as follows (no vaccination is just a special case with US$0 cost and no efficacy). If vaccines A and B have efficacies ε_A_ > ε_B_ and their costs are *V*_A_ > *V*_B_, choosing vaccine A over vaccine B would be cost-effective if and only if (*V*_A_ − *V*_B_)/(ε_A_ − ε_B_) < EVC_max_. For each possible combination of costs for the three vaccine candidates, we used this criterion to identify the candidate that has the highest probability of being the optimal vaccine by assuming that 1 − vaccine efficacy for the three vaccines followed log-normal distributions such that the medians and 2.5th and 97.5th percentiles of the associated vaccine efficacies were the same as the point estimates and 95% CIs of the vaccine efficacies reported in the clinical trials [12–14]. Please see “Comparative CEA of the Three Vaccine Candidates” in S1 Text for more details.

## Results

### Base Case

**Fig 3:**
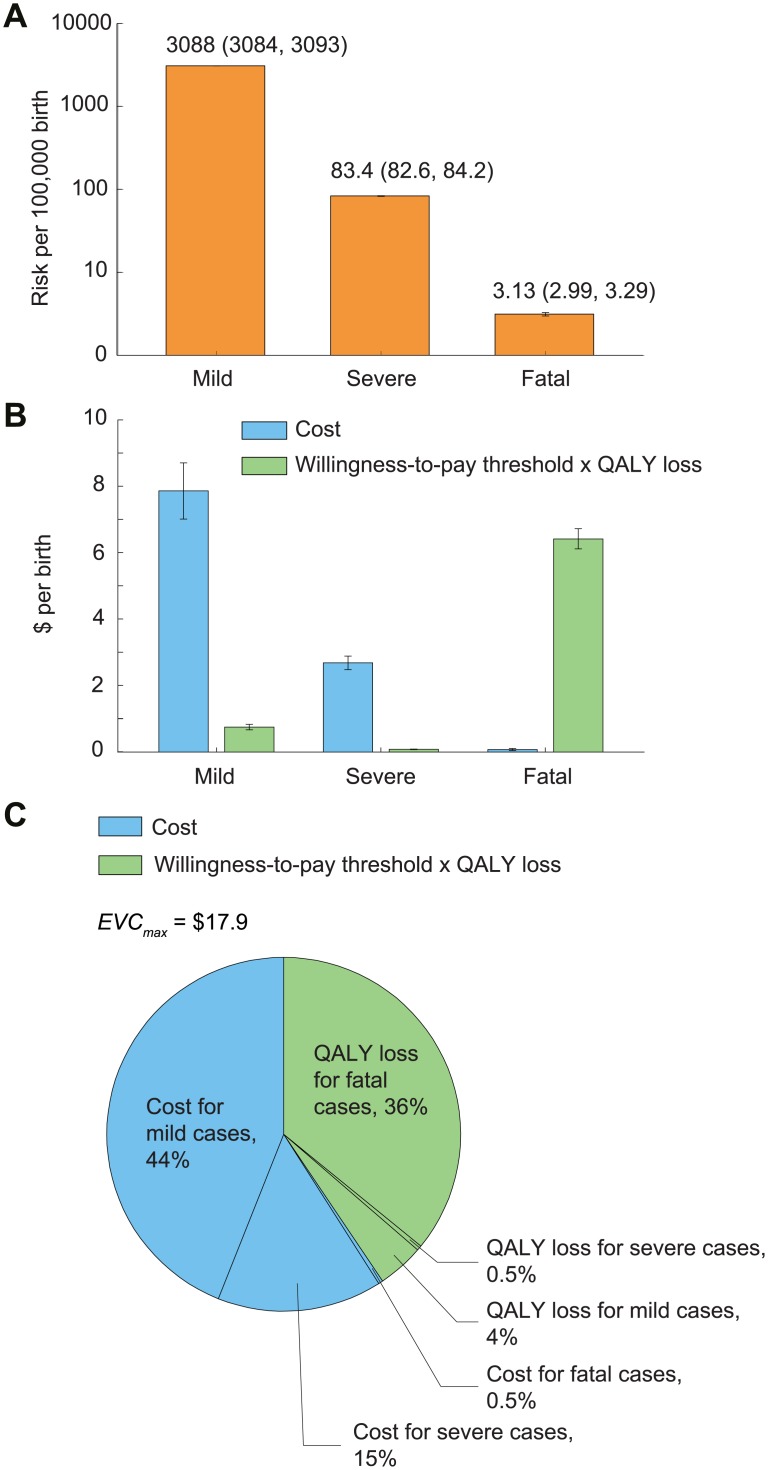
The risk, expected costs, and health utility loss of EV71-HFMD per birth in the base case. Error bars indicate 95% CIs. (A) Risk of mild, severe, and fatal EV71-HFMD. The 95% CIs are annotated because they are not graphically apparent. (B) Expected costs and health utility loss attributable to EV71-HFMD stratified by severity. To indicate the relative contribution of cost and health utility loss in EVC_max_, health utility loss was expressed in monetary terms as the product of QALY loss and willingness-to-pay threshold, which is set at one times GDPpc (US$6,700). (C) Percentage breakdown of EVC_max_.

In the base case, the risk that a child would develop mild, severe, and fatal EV71-HFMD between age 6 mo and 5 y was 3,088 (95% CI 3,084–3,093), 83.4 (95% CI 82.6–84.2), and 3.1 (95% CI 3.0–3.3) per 100,000, respectively (Fig 3A; Table 1). The estimated cost per birth attributed to EV71-HFMD was US$10.6 (95% CI US$9.7–US$11.5); 74%, 25%, and 1% of this cost was incurred by mild, severe, and fatal cases, respectively (Fig 3B). Pediatric EV71 vaccination would be cost-saving if EVC was below this amount.

The expected QALY loss attributed to EV71-HFMD per 10,000 births was 10.8 (10.7–10.9); 89%, 10%, and 1% of this loss was incurred by fatal (almost entirely due to premature death), mild, and severe cases, respectively (Fig 3B). With a willingness-to-pay threshold of one times GDPpc, pediatric EV71 vaccination was cost-effective if EVC was below US$17.9 (US$16.9–US$18.8). The reduction in costs and averted QALY loss (in monetary terms) constituted 60% and 40% of the net monetary benefit of vaccination, respectively (Fig 3C).

### Uncertainty Scenarios

**Fig 4:**
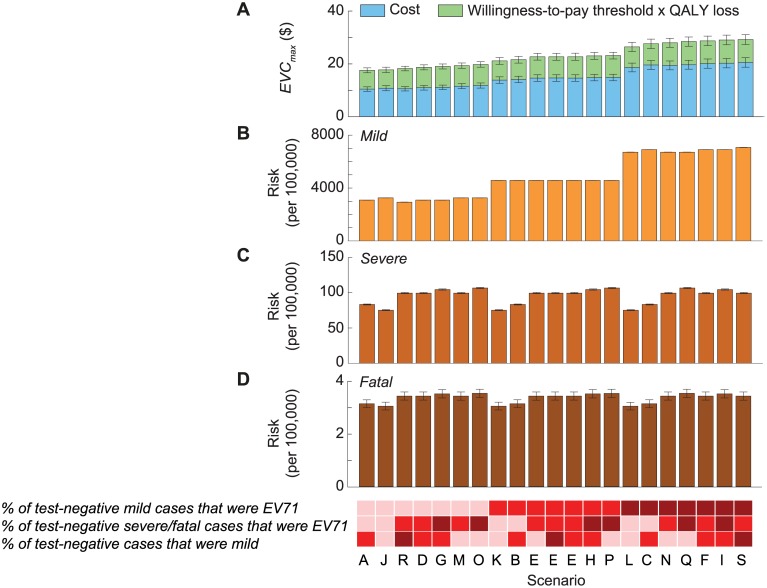
Cost-effectiveness of routine pediatric EV71 vaccination in China with a willingness-to-pay threshold of one times GDPpc. The 21 scenarios from Fig 2 are listed along the *x*-axis in ascending order of EVC_max_. The base case was scenario A, which had the lowest EVC_max_. The color shades at the bottom indicate the assumptions regarding the percentage of test-negative cases that were mild during 2010–2012 (bottom row), the percentage of test-negative severe/fatal cases that were EV71-HFMD (middle row), and the percentage of test-negative mild cases that were EV71-HFMD (top row) in each scenario, where darker shades correspond to higher percentages. As described in Fig 2B, three combinations of these percentages resulted in the same scenario, namely scenario E. The size of the 95% prediction intervals was driven by the parametric uncertainty associated with our estimates of the risk of EV71-HFMD and the mean cost and QALY loss attributed to EV71-HFMD in our probability sensitivity analysis (as in Fig 2B). (A) Pediatric EV71 vaccination would be cost-saving and cost-effective if EVC was below the cost of EV71-HFMD (blue bars) and EVC_max_ (blue plus green bars), respectively. (B–D) The risk of mild, severe, and fatal EV71-HFMD in each scenario. The error bars show the 95% CIs, but in some cases they are not apparent for the risk of mild and severe EV71-HFMD.

Mild cases were a major constituent of EVC_max_ (Fig 3C), and the risk of mild EV71-HFMD, and hence EVC_max_, strongly depended on the percentage of test-negative mild cases that were EV71 (Table 2; Fig 4A and 4B). In contrast, EVC_max_ was relatively insensitive to the percentage of test-negative severe/fatal cases that were EV71 because (i) severe cases were only a minor constituent of EVC_max_ (Fig 3C) and the risk of severe EV71-HFMD varied less across scenarios (Fig 4C), and (ii) although fatal cases were a major constituent of EVC_max_ (Fig 3C), the risk of fatal EV71-HFMD was almost constant across scenarios (Fig 4D). Compared to EVC_max_ in the base case (which was the lowest and hence the most conservative among all scenarios), EVC_max_ increased by (i) 19%–32% if the percentage of test-negative mild cases that were EV71 was the same as that for test-positive mild cases (scenarios K, B, E, H, and P in Tables 2 and S5 and Fig 4A) and (ii) 50%–67% if all test-negative mild cases were EV71 (scenarios L, C, N, Q, F, I, and S in Tables 2 and S5 and Fig 4A).

**Table 2:** Cost-effectiveness ceiling on effective vaccine cost if all EV71-HFMD cases were registered by the national HFMD surveillance.

Scenario	Societal Perspective				Excluding Productivity Loss of Parents/Caregivers			
	3% Discounting		6% Discounting		3% Discounting		6% Discounting	
	WTP 1 × GDPpc	WTP 3 × GDPpc	WTP 1 × GDPpc	WTP 3 × GDPpc	WTP 1 × GDPpc	WTP 3 × GDPpc	WTP 1 × GDPpc	WTP 3 × GDPpc
A (base case)	17.9 (16.9–18.8)	32.4 (31.0–33.7)	14.6 (13.7–15.4)	23.5 (22.5–24.6)	16.0 (15.2–16.8)	30.5 (29.3–31.7)	12.8 (12.1–13.5)	21.7 (20.8–22.7)
B	22.0 (20.6–23.3)	37.2 (35.5–38.8)	18.5 (17.2–19.7)	28.1 (26.7–29.5)	19.3 (18.2–20.4)	34.5 (33.0–36.0)	15.9 (14.9–16.9)	25.5 (24.3–26.7)
C	28.1 (26.2–29.9)	44.4 (42.3–46.6)	24.3 (22.5–26.0)	34.9 (33.0–36.9)	24.1 (22.6–25.6)	40.5 (38.6–42.3)	20.5 (19.1–21.9)	31.2 (29.5–32.8)
D	19.0 (18.1–20.0)	34.7 (33.4–36.1)	15.4 (14.6–16.3)	25.1 (24.0–26.2)	17.1 (16.3–17.9)	32.8 (31.6–34.1)	13.7 (12.9–14.4)	23.3 (22.3–24.3)
E	23.1 (21.8–24.4)	39.5 (37.9–41.2)	19.3 (18.1–20.6)	29.7 (28.2–31.1)	20.4 (19.3–21.5)	36.8 (35.3–38.3)	16.7 (15.7–17.8)	27.1 (25.8–28.3)
F	29.2 (27.4–31.1)	46.8 (44.6–49.0)	25.1 (23.4–26.9)	36.5 (34.6–38.5)	25.2 (23.7–26.8)	42.8 (40.9–44.7)	21.4 (19.9–22.8)	32.7 (31.1–34.4)
G	19.3 (18.4–20.3)	35.4 (34.1–36.8)	15.7 (14.8–16.6)	25.6 (24.5–26.6)	17.5 (16.6–18.3)	33.5 (32.3–34.8)	13.9 (13.2–14.7)	23.8 (22.8–24.7)
H	23.4 (22.1–24.8)	40.2 (38.6–41.9)	19.6 (18.4–20.8)	30.1 (28.7–31.5)	20.7 (19.6–21.8)	37.5 (36.0–39.0)	17.0 (16.0–18.0)	27.5 (26.3–28.8)
I	29.6 (27.7–31.4)	47.5 (45.3–49.7)	25.4 (23.7–27.1)	37.0 (35.1–38.9)	25.6 (24.0–27.1)	43.5 (41.6–45.4)	21.6 (20.2–23.0)	33.2 (31.5–34.8)
J	18.1 (17.0–19.1)	32.3 (30.9–33.6)	14.8 (13.9–15.8)	23.6 (22.5–24.7)	16.1 (15.2–17.0)	30.3 (29.0–31.6)	13.0 (12.2–13.8)	21.8 (20.8–22.8)
K	21.5 (20.2–22.8)	36.3 (34.7–38.0)	18.1 (16.9–19.3)	27.5 (26.1–28.9)	18.8 (17.7–19.9)	33.6 (32.2–35.1)	15.5 (14.5–16.5)	24.9 (23.7–26.1)
L	26.9 (25.1–28.7)	42.7 (40.6–44.8)	23.2 (21.5–24.8)	33.5 (31.7–35.4)	23.0 (21.6–24.5)	38.9 (37.1–40.7)	19.6 (18.2–20.9)	29.9 (28.3–31.5)
M	19.7 (18.6–20.7)	35.5 (34.0–36.9)	16.1 (15.1–17.0)	25.8 (24.7–26.9)	17.7 (16.8–18.6)	33.5 (32.2–34.8)	14.2 (13.4–15.0)	23.9 (22.9–25.0)
N	28.5 (26.7–30.2)	45.9 (43.8–48.0)	24.4 (22.8–26.1)	35.7 (33.8–37.6)	24.6 (23.2–26.0)	42.1 (40.2–43.9)	20.8 (19.4–22.1)	32.1 (30.5–33.6)
O	20.1 (19.1–21.2)	36.4 (34.9–37.8)	16.4 (15.5–17.4)	26.4 (25.3–27.6)	18.1 (17.2–19.0)	34.4 (33.0–35.7)	14.5 (13.7–15.4)	24.5 (23.5–25.6)
P	23.6 (22.2–24.9)	40.4 (38.8–42.1)	19.7 (18.4–20.9)	30.3 (28.8–31.7)	20.8 (19.7–21.9)	37.7 (36.2–39.2)	17.1 (16.0–18.1)	27.7 (26.4–28.9)
Q	28.9 (27.2–30.7)	46.8 (44.7–49.0)	24.8 (23.1–26.4)	36.3 (34.5–38.2)	25.1 (23.6–26.5)	42.9 (41.1–44.8)	21.1 (19.8–22.4)	32.6 (31.1–34.2)
R	18.6 (17.7–19.5)	34.2 (32.9–35.5)	15.0 (14.2–15.8)	24.6 (23.6–25.6)	16.7 (16.0–17.5)	32.4 (31.2–33.6)	13.3 (12.6–14.0)	22.9 (21.9–23.8)
S	29.7 (27.8–31.6)	47.4 (45.1–49.6)	25.6 (23.8–27.4)	37.1 (35.1–39.1)	25.6 (24.1–27.2)	43.3 (41.3–45.2)	21.7 (20.3–23.2)	33.2 (31.5–34.9)

The mean and 95% CI of EVC_max_ are listed for each scenario.

WTP, willingness-to-pay threshold.

When productivity loss of parents/caregivers was excluded, EVC_max_ was 10%–14% lower (Tables 2 and S5; S2 Fig). EVC_max_ was very sensitive to the willingness-to-pay threshold. Increasing the threshold from one to three times GDPpc increased EVC_max_ by 58%–84% (Tables 2 and S5; S2 Fig).

EVC_max_ was sensitive to discount rate because QALY loss due to pediatric premature death—which was the major driver for health utility loss and a major constituent of EVC_max_ (Fig 3B and 3C)—was very sensitive to the discount rate. Increasing the discount rate for cost and health utility from 3% to 6% reduced EVC_max_ by around 14%–19% (Tables 2 and S5; S2 Fig).

We estimated that the aggregated incidence rate of EV71-HFMD in the EV71 vaccine phase III trials and the corresponding rate in national surveillance were 18.3 (95% CI 16.3–20.6) and 13.4 (95% CI 12.4–14.5) per 1,000 person-years, respectively. These estimates suggest that only 74% (95% CI 64%–84%) of EV71-HFMD cases in these areas were registered by national surveillance during the trial periods. If this proportion is generalizable to the whole country and the expected cost and QALY loss of registered and unregistered cases are similar, then EVC_max_ is increased by 36% (95% CI 23%–50%) (Tables 2 and 3; S3 Fig).

**Table 3:** Cost-effectiveness ceiling on effective vaccine cost if the proportion of EV71-HFMD cases registered by national surveillance was the same as that observed in the three EV71 vaccine trials.

Scenario	Societal Perspective				Excluding Productivity Loss of Parents/Caregivers			
	3% Discounting		6% Discounting		3% Discounting		6% Discounting	
	WTP 1 × GDPpc	WTP 3 × GDPpc	WTP 1 × GDPpc	WTP 3 × GDPpc	WTP 1 × GDPpc	WTP 3 × GDPpc	WTP 1 × GDPpc	WTP 3 × GDPpc
A (base case)	24.4 (20.9–28.2)	44.1 (38.1–50.9)	19.9 (17.0–23.1)	32.1 (27.6–37.1)	21.8 (18.8–25.2)	41.6 (35.9–48.0)	17.5 (15.0–20.3)	29.6 (25.6–34.3)
B	29.9 (25.7–34.7)	50.7 (43.7–58.5)	25.2 (21.5–29.3)	38.3 (32.9–44.3)	26.2 (22.5–30.4)	47.0 (40.5–54.2)	21.7 (18.5–25.2)	34.8 (30.0–40.2)
C	38.3 (32.7–44.5)	60.5 (52.1–70.0)	33.1 (28.2–38.6)	47.6 (40.9–55.2)	32.9 (28.1–38.2)	55.1 (47.5–63.7)	28.0 (23.9–32.6)	42.5 (36.5–49.2)
D	25.9 (22.3–30.0)	47.3 (40.9–54.7)	21.1 (18.1–24.4)	34.2 (29.5–39.5)	23.3 (20.1–27.0)	44.8 (38.6–51.6)	18.6 (16.0–21.6)	31.8 (27.4–36.7)
E	31.5 (27.0–36.5)	53.9 (46.4–62.2)	26.3 (22.5–30.6)	40.4 (34.7–46.7)	27.8 (23.8–32.2)	50.2 (43.3–57.9)	22.8 (19.5–26.5)	36.9 (31.7–42.7)
F	39.8 (34.0–46.4)	63.8 (54.8–73.8)	34.2 (29.2–40.0)	49.8 (42.7–57.7)	34.4 (29.5–40.0)	58.3 (50.2–67.4)	29.1 (24.8–33.9)	44.6 (38.3–51.7)
G	26.4 (22.7–30.6)	48.3 (41.6–55.8)	21.4 (18.4–24.8)	34.8 (30.0–40.3)	23.8 (20.5–27.5)	45.7 (39.5–52.8)	19.0 (16.3–22.0)	32.4 (27.9–37.4)
H	32.0 (27.4–37.1)	54.9 (47.2–63.4)	26.7 (22.8–31.1)	41.1 (35.3–47.6)	28.2 (24.2–32.7)	51.2 (44.1–59.1)	23.2 (19.8–26.9)	37.6 (32.3–43.4)
I	40.3 (34.4–46.9)	64.8 (55.7–74.9)	34.6 (29.5–40.4)	50.4 (43.3–58.5)	34.9 (29.9–40.5)	59.3 (51.1–68.6)	29.5 (25.1–34.3)	45.3 (38.9–52.4)
J	24.6 (21.1–28.6)	44.0 (38.0–50.8)	20.2 (17.3–23.5)	32.2 (27.7–37.2)	22.0 (18.9–25.5)	41.3 (35.7–47.7)	17.7 (15.2–20.6)	29.7 (25.6–34.3)
K	29.3 (25.1–34.0)	49.5 (42.7–57.2)	24.6 (21.0–28.7)	37.4 (32.1–43.3)	25.6 (22.0–29.7)	45.8 (39.5–53.0)	21.2 (18.1–24.6)	34.0 (29.2–39.3)
L	36.6 (31.3–42.7)	58.2 (50.1–67.5)	31.6 (27.0–36.9)	45.7 (39.2–53.0)	31.4 (26.9–36.5)	53.0 (45.6–61.3)	26.6 (22.7–31.1)	40.7 (35.0–47.3)
M	26.8 (23.0–31.0)	48.4 (41.7–55.8)	21.9 (18.8–25.4)	35.2 (30.3–40.6)	24.1 (20.7–27.9)	45.7 (39.4–52.7)	19.3 (16.6–22.5)	32.6 (28.1–37.7)
N	38.8 (33.2–45.2)	62.7 (53.9–72.4)	33.3 (28.4–38.8)	48.7 (41.8–56.4)	33.6 (28.7–38.9)	57.4 (49.4–66.2)	28.3 (24.2–32.9)	43.7 (37.6–50.5)
O	27.4 (23.5–31.8)	49.6 (42.8–57.3)	22.4 (19.2–26.0)	36.0 (31.0–41.6)	24.7 (21.3–28.6)	46.9 (40.5–54.1)	19.8 (17.0–23.0)	33.4 (28.8–38.6)
P	32.1 (27.6–37.2)	55.1 (47.5–63.6)	26.8 (23.0–31.2)	41.2 (35.5–47.7)	28.4 (24.3–32.9)	51.4 (44.3–59.4)	23.3 (19.9–27.0)	37.7 (32.4–43.5)
Q	39.5 (33.8–45.8)	63.9 (55.0–73.8)	33.8 (28.9–39.3)	49.5 (42.6–57.4)	34.2 (29.3–39.6)	58.6 (50.5–67.6)	28.8 (24.6–33.4)	44.5 (38.3–51.4)
R	25.3 (21.8–29.3)	46.6 (40.2–53.8)	20.5 (17.6–23.7)	33.5 (28.9–38.7)	22.8 (19.7–26.4)	44.2 (38.1–51.0)	18.1 (15.6–21.0)	31.2 (26.9–36.0)
S	40.5 (34.6–47.1)	64.6 (55.5–74.6)	34.9 (29.7–40.7)	50.6 (43.3–58.6)	35.0 (29.9–40.6)	59.0 (50.8–68.2)	29.6 (25.3–34.5)	45.3 (38.9–52.4)

The mean and 95% CI of EVC_max_ are listed for each scenario.

WTP, willingness-to-pay threshold.

**Fig 5:**
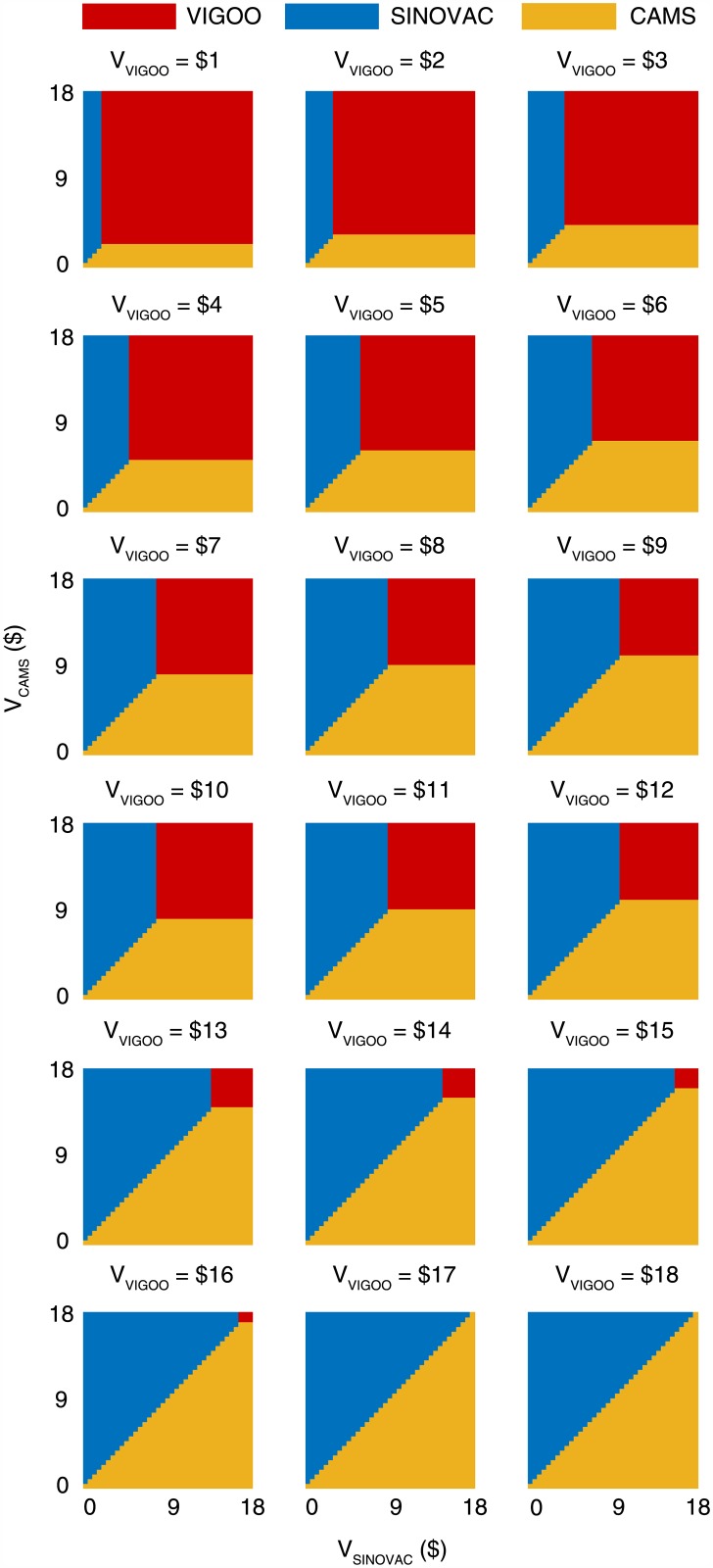
Comparative cost-effectiveness analysis of the three vaccine candidates based on the base-case EVC_max_. The color coding indicates the optimal vaccine given the costs of the three vaccines (*V*_Vigoo_, *V*_Sinovac_, *V*_CAMS_).

The efficacy estimates for the vaccines made by Beijing Vigoo Biological, Sinovac Biotech, and the Chinese Academy of Medical Sciences (CAMS) were 90% (95% CI 67.1%–96.9%), 94.8% (95% CI 87.2%–97.9%), and 97.4% (95% CI 92.9%–99%), respectively [12–14]. Using the base-case EVC_max_, we estimated that EV71 vaccination would be cost-effective in China if the vaccine cost was below US$12.0–US$17.7, US$15.3–US$18.0, and US$16.2– US$18.3 for the Beijing Vigoo Biological, Sinovac Biotech, and CAMS vaccines, respectively, when compared to no vaccination. Fig 5 indicates the optimal vaccine given any combination of the costs for the three vaccines within these ranges. For example, if all three vaccines were priced at the point estimate of EVC_max_ × vaccine efficacy (which corresponds to US$16.1, US$17.0, and US$17.4 for the Beijing Vigoo Biological, Sinovac Biotech, and CAMS vaccine, respectively), then the CAMS vaccine would be optimal.

## Discussion

Our CEA suggests that routine pediatric EV71 vaccination in China would be very cost-effective if the all-inclusive EVC (including all logistical, procurement, and administration costs needed to confer 5 y of vaccine protection) is below US$17.9 (95% CI US$16.9–US$18.8). Compared to no vaccination, routine pediatric EV71 vaccination would be cost-effective in China if the vaccine cost is below US$12.0–US$17.7, US$15.3–US$18.0, and US$16.2–US$18.3 for the Beijing Vigoo Biological, Sinovac Biotech, and CAMS vaccines, respectively. Given any combination of the costs for the three vaccines within these ranges, policymakers can use Fig 5 to determine the optimal vaccine.

Our estimates of cost-effective vaccine cost ceiling are very conservative for the following reasons. First, we have likely underestimated the true disease burden of EV71, and hence EVC_max_, because (i) EV71 can cause diseases other than HFMD [9] and (ii) underreporting and under-ascertainment of EV71-HFMD, as we have demonstrated, might be substantial (Tables 2 and 3; S2 and S3 Figs).

Second, we assumed that EV71 vaccination had no effect in reducing the severity of EV71-HFMD because there were too few severe cases in the phase III trials to provide robust evidence for such efficacy [12–14]. If EV71 vaccines, like many other vaccines such as rotavirus vaccines [23], can reduce disease severity, then we have underestimated EVC_max_.

Third, we used one times GDPpc as the base-case willingness-to-pay threshold, in keeping with the WHO-CHOICE criterion for very cost-effective interventions [20]. The commonly used thresholds in high-income countries, such as the UK (£20,000–£30,000) [24], are close to one times GDPpc. WHO-CHOICE classifies interventions as cost-effective if the ICER is between one and three times GDPpc. This range of willingness-to-pay threshold might also be suitable for China, which is a middle-income country with a rapidly increasing GDPpc. As shown, EVC_max_ would increase by 60%–80% if the threshold was three times GDPpc (S2 Fig). On the other hand, there have been ongoing discussions about the appropriateness of the WHO-CHOICE thresholds, and some preliminary work suggests a real-world threshold of around 0.18–0.71 times GDPpc in middle- and high-income countries [25]. As an illustration, adopting a threshold of 0.5 times GDPpc would reduce EVC_max_ by around 25%.

Finally, we did not account for productivity loss due to pediatric premature death because of unresolved debate about the way such losses should be costed [18]. Including this productivity loss would increase EVC_max_.

Our study has several limitations. First, we have presented our CEA results using EVC, an outcome with which readers may be less familiar compared to other outcomes commonly used in health economic evaluation, such as ICERs. The calculation underlying EVC, however, is the same as that for evaluating the incremental net monetary benefits of vaccination. We used EVC as the outcome because (i) health policymakers in China are currently most interested in the vaccine cost ceiling that would render routine pediatric EV71 vaccination cost-effective in order to prepare for a potential future addition to the Expanded Programme on Immunization and price negotiation with the vaccine manufacturers, and (ii) the reported vaccine efficacy and associated uncertainty for the three EV71 vaccine candidates are significantly different. As such, EVC, which is simply the vaccine cost adjusted for the corresponding vaccine efficacy, provides a succinct way of defining the vaccine cost ceiling without the need to reference the efficacy of any particular vaccine. As we have shown, EVC can be easily used to compare the cost-effectiveness of the three vaccine candidates, which have significantly different vaccine efficacy and hence vaccine cost ceilings.

Second, we have not accounted for the potential impact of an increase in the age of infection caused by herd immunity or limited duration of vaccine protection. Our previous study showed that the severity of EV71-HFMD decreases substantially with age [6]. Therefore, the cost-effectiveness of pediatric vaccination would be only slightly reduced if some of the burden averted by vaccination among children under 5 y is offset by increased infection in older age groups.

Third, we used self-reported hospital charges as a proxy for the opportunity cost of care, because of the difficulty of accessing hospital records across the entire country. Several studies have estimated the economic cost of HFMD treatment in single cities or counties in China using hospital records [26–34]. However, only two of these, one set in Ningbo in Zhejiang and the other in seven cities in Shandong, reported mean costs using hospital records and the same severity stratification as the national surveillance database [29,34]. The Ningbo study reported mean costs of US$161, US$530, and US$1,262, respectively, for mild outpatient, mild inpatient, and severe cases, whereas the estimates in the Shandong study were US$124, US$699, and US$2,076, respectively. In comparison, the corresponding costs in our study for the east region of China (where Ningbo and Shandong are located) were US$239, US$1,011, and US$3,592. The costs for mild cases in these two studies and ours differ by less than 48%, which suggests that our estimate of EVC_max_ should be reasonably robust because mild cases are the main cost driver (Fig 3C) and our CEA assumptions are very conservative.

Fourth, we modified the EuroQol EQ-5D-3L instument to elicit quality of life of children younger than 7 y, as has been done previously in similar studies [35]. Although such modifications have not been validated, the associated uncertainty had little effect on our results because 89% of QALY loss due to EV71-HFMD came from premature death (Fig 3C).

Fifth, we assumed that the cost and health utility loss estimates based on laboratory-confirmed EV71-HFMD cases were representative of all EV71-HFMD cases.

Sixth, we assumed that the long-term average risk of EV71-HFMD in the future would be similar to that registered by national surveillance during 2010–2013. Year-to-year variations in EV71-HFMD burden can be significant, and future EV71-HFMD epidemiology may not be congruent with this assumption. The confidence intervals associated with our estimates of EVC_max_ do not reflect possible deviations of future EV71-HFMD burden from the long-term average that we have assumed. For example, if future long-term EV71-HFMD were more similar to that in 2012 or 2013, which were the years with the highest and lowest EV71-HFMD burden, then EVC_max_ would be increased by 42% and decreased by 47%, respectively.

Finally, our analysis assumed that no alternative preventive interventions are available to be used that could also reduce the burden of EV71-HFMD. If such interventions exist, this may affect the cost-effectiveness of vaccination. We conducted a literature review of the potential effectiveness of alternative interventions to prevent the spread of EV71 (see “Under What Conditions Would We Not Vaccinate Because a More Cost-Effective Alternative Intervention Exists?” in S1 Text for details). The review found only one relevant article, which suggested that the extreme infection control measures imposed during the 2003 SARS outbreak in Hong Kong reduced the incidence of HFMD by 57.2%. However, these reactive control measures, which involved territory-wide school closure, hand and respiratory hygiene awareness campaigns, and disinfection, are unlikely to be reproducible for prolonged periods outside a pandemic or highly urbanized setting. Nevertheless, if such a level of reduction in HFMD incidence is achievable without using vaccination, then EVC_max_ would be reduced by 57.2% to US$7.3.

To our knowledge, this is the first economic evaluation of EV71 vaccination since the EV71 vaccine phase III trial results were published in 2013–2014. In 2010, Lee et al. [36] used the national surveillance data from 2007–2009 and extrapolated US health care cost figures to China to forecast that with a willingness-to-pay to avert a disability-adjusted life year of three times GDPpc, pediatric EV71 vaccination would be cost-effective from a third-party payer perspective if vaccine cost per individual was US$25 and vaccine efficacy was ≥70% [36]. The assumed risk of EV71-HFMD in their study (0.04%–1%) was much lower than in ours (3%–8%; Fig 4B) because the disease burden registered by national surveillance has increased substantially since 2009. In their sensitivity analysis, Lee et al. found that when the risk of EV71-associated diseases was 5% (i.e., similar to that in the intermediate scenarios in Fig 4B), pediatric EV71 vaccination would be cost-saving if vaccine cost was US$50 and vaccine efficacy was 80% (i.e., an EVC of US$63). In contrast, pediatric EV71 vaccination would be cost-saving in the corresponding scenarios of our study only if EVC < US$12.2 (S2 Fig). Therefore, the discrepancies between the two studies are also due to differences in cost estimates.

Our study is also the first to our knowledge to highlight that the current practice of omitting test-negative results generates substantial uncertainty regarding the burden of EV71-HFMD (Fig 4B). Laboratory surveillance is the cornerstone for monitoring diseases caused by multiple strains of pathogens, such as HFMD, pneumococcal diseases, etc. In our base case (in which we assumed that none of the test-negative cases were EV71-HFMD), EV71 accounted for 26.0% and 64.3% of mild and severe/fatal HFMD cases, respectively, in China during 2010–2013. Policymakers can regard this as the maximal proportion of HFMD burden that is preventable by routine pediatric EV71 vaccination. If routine pediatric EV71 vaccination is implemented and vaccine coverage is high, robust laboratory surveillance of HFMD-causing enteroviruses will be critical for monitoring vaccine impact as well as investigating herd immunity, cross-protection, and serotype replacement (e.g., as demonstrated for pneumococcal conjugate vaccination [16,37,38]).

Our current and previous study [6] suggest that there are large geographical variations in the risk of EV71-HFMD (S4 Fig). Such variations are likely due to geographical differences in underlying epidemiologic factors, underreporting, and health care quality. If national, population-wide rollout is infeasible in the initial stages of routine pediatric EV71 vaccination, policymakers could consider prioritizing areas in which EV71 vaccination is most cost-effective.

## Supporting Information

S1 Data HFMD national and virological surveillance data from 31 provinces during 2010–2013. (CSV)

S2 Data Telephone survey data on household costs and quality of life detriments associated with EV71-HFMD. (CSV)

S3 Data Telephone survey questionnaire for caregivers in simplified Chinese. (DOCX)

S4 Data Explanations for variables for S1 and S2 Data. (DOCX)

S1 Fig Comparison of the incidence reduction predicted in the static model and that predicted in the TSIR dynamic model in Takahashi et al. [16]. The TSIR model predictions presented here were generated using the model in Figure 3D of Takahashi et al. [16] for 2, 5, 10, 20, 30, and 50 y after vaccination has begun. Predictions in the static and dynamic models are essentially the same because the basic reproductive number is high (with a national average of 27). Incidence in the TSIR dynamic model was slightly higher than that in the static model when vaccine coverage was near one because the epidemic had not yet completely reached equilibrium.
(EPS)

S2 Fig Cost-effectiveness of routine pediatric EV71 vaccination in China. (A) Base case, i.e., same as Fig 3A. (B–H) All other scenarios considered in the uncertainty analysis by including or excluding productivity loss, discounting cost and health utility at 3% or 6%, and setting the willingness-to-pay threshold at one or three times GDPpc.
(EPS)

S3 Fig Cost-effectiveness of routine pediatric EV71 vaccination in China accounting for the effect of underreporting. Same as S2 Fig but the proportion of EV71-HFMD cases registered by national surveillance was assumed to be the same as that estimated from the three EV71 vaccine trials, i.e., 74%(95% CI 64%–84%). See “Estimation of the Unregistered Proportion” in S1 Text and S15 Table for details.
(EPS)

S4 Fig Geographical variation in the risk and severity of EV71-HFMD among children under the age of 5 y in China. (A–C) Risk of mild, severe, and fatal cases of EV71-HFMD. (D–F) Case severity, case fatality, and severity–fatality risk.
(EPS)

S5 Fig The distributions of costs and QALY loss per episode of EV71-HFMD in the survey stratified by severity and geographical region. (A) Costs. (B) QALY loss during illness. In each box, the central mark is the median, the edges of the box are the 25th and 75th percentiles, the whiskers extend to the extreme data points not considered outliners, and outliners are plotted individually with “+” marks. Data points are considered outliners if they are larger than *q*_75_ + 1.5 × (*q*_75_ − *q*_25_) or smaller than *q*_25_ − 1.5 × (*q*_75_ − *q*_25_), where *q*_25_ and *q*_75_ are the 25th and 75th percentiles.
(EPS)

S6 Fig EVC_max_ as a function of the effectiveness of a more cost-effective alternative (e.g., hand hygiene or social distancing). Solid and dashed lines indicate the mean and 95% CI.
(EPS)

S7 Fig The sex, age, and serotype distribution of survey respondents, survey non-respondents, and laboratory-confirmed cases in the national surveillance in each of the seven geographical regions. (A) Mild outpatient cases. (B) Mild inpatient cases. (C) Severe cases. Sex and age distribution were obtained from laboratory-confirmed EV71-HFMD cases, and serotype distribution was from laboratory-confirmed HFMD cases.
(EPS)

S1 Table The percentage of EV71-HFMD cases among all severe/fatal HFMD cases in each province in each of the 19 test-negative scenarios. (DOCX)

S2 Table The percentage of EV71-HFMD cases among all mild HFMD cases in each province in each of the 19 test-negative scenarios. (DOCX)

S3 Table The percentage of EV71-HFMD cases among severe/fatal test-negative cases in each province in each of the 19 test-negative scenarios. (DOCX)

S4 Table The percentage of EV71-HFMD cases among mild test-negative cases in each province in each of the 19 test-negative scenarios. (DOCX)

S5 Table Changes in EVC_max_ across the scenarios considered in Table 2. In the first column (i.e., societal perspective, 3% discount rate, willingness-to-pay threshold of 1 × GDPpc), the point estimates and 95% confidence intervals of EVC_max_ in scenarios B–S are compared to that in the base case (i.e., scenario A). For the remaining columns, the point estimates and 95% confidence intervals of EVC_max_ in each scenario are compared to their counterparts in the first column, i.e., EVC_max_ in scenario *X* of column *Y* was compared to EVC_max_ in scenario *X* of column 1.
(DOCX)

S6 Table Seven regions in China from which equal representation in the sample of our telephone survey was obtained. (DOCX)

S7 Table Demographic characteristics and geographic distribution of 1,787 EV71-HFMD patients whose parents or caregivers were telephone survey participants. (DOCX)

S8 Table Costs for 1,787 EV71-HFMD patients whose parents or caregivers were telephone survey participants (mean, in US dollars). (DOCX)

S9 Table QALY loss during illness for 1,787 EV71-HFMD patients whose parents or caregivers were telephone survey participants (mean). (DOCX)

S10 Table Average annual income in 2013 (urban income, rural income, and income weighted by urban and rural population) and the percentage of mild HFMD cases that were inpatients in each of the 31 provinces. (DOCX)

S11 Table Association of costs and QALY loss with age, gender, and urban residence status. A Kruskal–Wallis test was performed in each severity–region stratum. Multiple testing of the same hypothesis in the seven different regions was corrected for using false discovery rate control. Associations with *p* < 0.05 are highlighted.
(DOCX)

S12 Table Mean, variance, and covariance of our survey data on cost and QALY loss per mild outpatient, mild inpatient, severe, and fatal case of EV71-HFMD in each region. (DOCX)

S13 Table Expected cost and QALY loss per case of mild, severe, and fatal EV71-HFMD in each of the 31 provinces. (DOCX)

S14 Table Estimated incidence rate of EV71-HFMD in the study areas of the EV71 vaccine phase III trials. (DOCX)

S15 Table Estimated incidence rate of EV71-HFMD in the national surveillance database in the study areas of the EV71 vaccine phase III trials. (DOCX)

S1 Text Model specifications and parameterization. (DOCX)

## Abbreviations

CAMS: Chinese Academy of Medical Sciences
CEA: cost-effectiveness analysis
EV71: enterovirus 71
EV71-HFMD: enterovirus-71-associated hand, foot, and mouth disease
EVC: effective vaccine cost
GDPpc: gross domestic product per capita
HFMD: hand, foot, and mouth disease
ICER: incremental cost-effectiveness ratio
OEV: other enterovirus
QALY: quality-adjusted life year
TSIR: time series susceptible–infected–recovered

## References

[1] Chua KB, Kasri AR. Hand foot and mouth disease due to enterovirus 71 in Malaysia. Virol Sin. 2011;26:221–8. 10.1007/s12250-011-3195-8 21847753 PMC8222466

[2] Ho M, Chen ER, Hsu KH, Twu SJ, Chen KT, Tsai SF, et al An epidemic of enterovirus 71 infection in Taiwan. Taiwan Enterovirus Epidemic Working Group. N Engl J Med. 1999;341:929–935. 10.1056/NEJM199909233411301 10498487

[3] Hosoya M, Kawasaki Y, Sato M, Honzumi K, Kato A, Hiroshima T, et al Genetic diversity of enterovirus 71 associated with hand, foot and mouth disease epidemics in Japan from 1983 to 2003. Pediatr Infect Dis J. 2006;25:691–694. 10.1097/01.inf.0000227959.89339.c3 16874167

[4] Nguyen NT, Pham HV, Hoang CQ, Nguyen TM, Nguyen LT, Phan HC, et al Epidemiological and clinical characteristics of children who died from hand, foot and mouth disease in Vietnam, 2011. BMC Infect Dis. 2014;14:341 10.1186/1471-2334-14-341 24942066 PMC4068316

[5] Wu Y, Yeo A, Phoon MC, Tan EL, Poh CL, Quak SH, et al The largest outbreak of hand; foot and mouth disease in Singapore in 2008: the role of enterovirus 71 and coxsackievirus A strains. Int J Infect Dis. 2010;14:e1076–e1081. 10.1016/j.ijid.2010.07.006 20952237

[6] Xing W, Liao Q, Viboud C, Zhang J, Sun J, Wu JT, et al Hand, foot, and mouth disease in China, 2008–12: an epidemiological study. Lancet Infect Dis. 2014;14:308–318. 10.1016/S1473-3099(13)70342-6 24485991 PMC4035015

[7] World Health Organization Western Pacific Region. Emerging disease surveillance and response—hand, foot and mouth disease (HFMD). [cited 29 Jan 2015]. http://www.wpro.who.int/emerging_diseases/HFMD/en/.

[8] Chinese Center for Disease Control and Prevention. National Population and Health Science Data Sharing Platform. [cited 29 Jan 2015]. http://www.phsciencedata.cn/Share/en/index.jsp.

[9] Ooi MH, Wong SC, Lewthwaite P, Cardosa MJ, Solomon T. Clinical features, diagnosis, and management of enterovirus 71. Lancet Neurol. 2010;9:1097–1105. 10.1016/S1474-4422(10)70209-X 20965438

[10] Solomon T, Lewthwaite P, Perera D, Cardosa MJ, McMinn P, Ooi MH. Virology, epidemiology, pathogenesis, and control of enterovirus 71. Lancet Infect Dis. 2010;10:778–790. 10.1016/S1473-3099(10)70194-8 20961813

[11] World Health Organization Western Pacific Region. A guide to clinical management and public health response for hand, foot and mouth disease (HFMD). Geneva: World Health Organization; 2011.

[12] Li R, Liu L, Mo Z, Wang X, Xia J, Liang Z, et al An inactivated enterovirus 71 vaccine in healthy children. N Engl J Med. 2014;370:829–837. 10.1056/NEJMoa1303224 24571755

[13] Zhu F, Xu W, Xia J, Liang Z, Liu Y, Zhang X, et al Efficacy, safety, and immunogenicity of an enterovirus 71 vaccine in China. N Engl J Med. 2014;370:818–828. 10.1056/NEJMoa1304923 24571754

[14] Zhu FC, Meng FY, Li JX, Li XL, Mao QY, Tao H, et al Efficacy, safety, and immunology of an inactivated alum-adjuvant enterovirus 71 vaccine in children in China: a multicentre, randomised, double-blind, placebo-controlled, phase 3 trial. Lancet. 2013;381:2024–2032. 10.1016/S0140-6736(13)61049-1 23726161

[15] Yu H, Yang W, Varma JK. To save children’s lives, China should adopt an initiative to speed introduction of pneumonia vaccines. Health Aff (Millwood). 2012;31:2545–2553. 10.1377/hlthaff.2011.1272 23129686

[16] Takahashi S, Liao Q, Van Boeckel TP, Xing W, Sun J, Hsiao VY, et al Hand, foot, and mouth disease in China: modelling epidemic dynamics of enterovirus serotypes and implications for vaccination. PLoS Med. 2016 10.1371/journal.pmed.1001958 PMC4755668 26882540

[17] The World Bank. Life expectancy at birth. [cited 29 Jan 2015]. http://data.worldbank.org/indicator/SP.DYN.LE00.IN.

[18] Koopmanschap MA, Rutten FF, van Ineveld BM, van Roijen L. The friction cost method for measuring indirect costs of disease. J Health Econ. 1995;14:171–189. 10154656 10.1016/0167-6296(94)00044-5

[19] Weinstein MC, Siegel JE, Gold MR, Kamlet MS, Russell LB. Recommendations of the Panel on Cost-effectiveness in Health and Medicine. JAMA. 1996;276:1253–1258. 8849754

[20] Tan-Torres Edejer T, Baltussen R, Adam T, Hutubessy R, Acharya A, Evans DB, et al Making choices in health: WHO guide to cost-effectiveness analysis. Geneva: World Health Organization; 2003.

[21] National Bureau of Statistics of China. National data: national accounts. [cited 29 Jan 2015]. http://data.stats.gov.cn/english/easyquery.htm?cn=C01.

[22] Zhu FC, Liang ZL, Meng FY, Zeng Y, Mao QY, Chu K, et al Retrospective study of the incidence of HFMD and seroepidemiology of antibodies against EV71 and CoxA16 in prenatal women and their infants. PLoS ONE. 2012;7:e37206 10.1371/journal.pone.0037206 22662137 PMC3360679

[23] Panozzo CA, Becker-Dreps S, Pate V, Weber DJ, Jonsson Funk M, Sturmer T, et al Direct, indirect, total, and overall effectiveness of the rotavirus vaccines for the prevention of gastroenteritis hospitalizations in privately insured US children, 2007–2010. Am J Epidemiol. 2014;179:895–909. 10.1093/aje/kwu001 24578359 PMC3969536

[24] National Institute for Health and Care Excellence. The guidelines manual: assessing cost effectiveness. 2012 Nov [cited 23 Mar 2015]. https://www.nice.org.uk/article/pmg6/chapter/7-assessing-cost-effectiveness.

[25] Woods B, Revill P, Sculpher M, Claxton K. Country-level cost-effectiveness thresholds: initial estimates and the need for further research. York: University of York Center for Health Economics; 2015. 10.1016/j.jval.2016.02.017 PMC5193154 27987642

[26] Lu Y, Dong Y, Xu Y. Cost-effectiveness analysis of 4 therapeutic schemes for children herpangina. China Pharmaceuticals. 2011;20:48–50.

[27] Tang X, Li W, Wang W. Cost-minimization analysis of three therapeutic schemes for hand-foot-mouth disease. China Mod Med. 2010;33:095.

[28] Yang T. Cost-effectiveness analysis of medication treatment for HFMD in children. Chin J Mod Drug Appl. 2012;6:79–80.

[29] He T-F, Yang T-C, Yi B, Xu G-Z. Economic burden estimation for pandemic hand foot and mouth disease in Ningbo City, 2011. Shanghai J Prev Med. 2012;9:001.

[30] Yang T-C, Yi B, He T-F, Wu Y, Ma Y, Xu G. Economic burden of hand, foot and mouth disease in Ningbo, Zhejiang. Dis Surveill. 2012;27:520–523.

[31] Qin Y, Zhang J, Zhang J, Mou G, Xing Y, Liu J. Investigation on economical burden and status of health care seeking among 1398 hand-foot-mouth disease cases in Yantai. Chin J Epidemiol. 2009;30:1319–1320.

[32] Gan Z-K, Jin H, Li J-X, Yao X-J, Zhou Y, Zhang X-F, et al Disease burden of enterovirus 71 in rural central China: a community-based survey. Hum Vaccin Immunother. 2015;11:2400–2405. 10.1080/21645515.2015.1059980 26158689 PMC4635911

[33] Li W, Li H, Li G-F, Hu Y-H. Survey on economic burden of hand, foot and mouth disease in Huaiyin District of Jinan City 2012. Prev Med Trib. 2014;7:484–487.

[34] Liu T. Research on economic burden of disease and its determinants of hand, foot and mouth disease patients in Shandong Province. Jinan: Shandong University; 2013.

[35] Martin A, Cottrell S, Standaert B. Estimating utility scores in young children with acute rotavirus gastroenteritis in the UK. J Med Econ. 2008;11:471–484. 10.3111/13696990802321047 19450099

[36] Lee BY, Wateska AR, Bailey RR, Tai JH, Bacon KM, Smith KJ. Forecasting the economic value of an enterovirus 71 (EV71) vaccine. Vaccine. 2010;28:7731–7736. 10.1016/j.vaccine.2010.09.065 20923711 PMC2989421

[37] Miller E, Andrews NJ, Waight PA, Slack MP, George RC. Herd immunity and serotype replacement 4 years after seven-valent pneumococcal conjugate vaccination in England and Wales: an observational cohort study. Lancet Infect Dis. 2011;11:760–768. 10.1016/S1473-3099(11)70090-1 21621466

[38] Tabrizi SN, Brotherton JM, Kaldor JM, Skinner SR, Liu B, Bateson D, et al Assessment of herd immunity and cross-protection after a human papillomavirus vaccination programme in Australia: a repeat cross-sectional study. Lancet Infect Dis. 2014;14:958–966. 10.1016/S1473-3099(14)70841-2 25107680

