# Correction: Routine Pediatric Enterovirus 71 Vaccination in China: a Cost-Effectiveness Analysis

**DOI:** 10.1371/journal.pmed.1002013

**Published:** 2016-04-06

**Authors:** Joseph T. Wu, Mark Jit, Yaming Zheng, Kathy Leung, Weijia Xing, Juan Yang, Qiaohong Liao, Benjamin J. Cowling, Bingyi Yang, Eric H. Y. Lau, Saki Takahashi, Jeremy J. Farrar, Bryan T. Grenfell, Gabriel M. Leung, Hongjie Yu

The following information is missing from the Funding section: YZ, WX, JY, QL and HY were supported by the TOTAL foundation (no. 2015–099).
