# Supplementary material for: Routine Pediatric Enterovirus 71 Vaccination in China: a Cost-Effectiveness Analysis
Source: PLoS Med. 2016 Mar 15;13(3):e1001975. doi: 10.1371/journal.pmed.1001975 (PMC4792415; doi:10.1371/journal.pmed.1001975)
Supplement: S3 Data — (DOCX) [file pmed.1001975.s003.docx]

问卷编号

<<涉外社会调查许可证>>批准机关:国家统计局

<<涉外社会调查许可证>>号:国统涉外证字第0389号

备案机关:国家统计局

**中国手足口病患者经济负担与生存质量电话调查表**

**患者家长问卷首页**

| 省份: 520 | | 河北 |  | | 辽宁 | | 江苏 | | | 广西 |
| --- | --- | --- | --- | --- | --- | --- | --- | --- | --- | --- |
|  | | 贵州 |  | | 陕西 | | 河南 | | |  |
|  | |  |  | |  | |  | | |  |
| 被访者**患儿**的类型：(521) | | 轻症（1） | | | 重症（2） | | | 死亡（3） | | |
|  | |  | | | | | | | | |
| 患儿年龄：(522) | 6个月~1岁（1）  4~5岁（5） | | | 1-2岁（2） | | 2-3岁（3） | | | 3~4岁（4） | |
|  | |  | | | | | | | | |

**请在访问结束后收集以下信息**

**调查表编号□□□□□□□□**

**患者姓名：** _______________

**家属姓名：** _______________

**电话号码：** _______________

**居住地址/工作单位：**

**□城市，** _______________**（请注明具体城市） □农村**

***本次采访将严格按照市场调查的行业规范进行***

采访员签字_______________ 采访日期________________采访长度_______________

**访谈者访谈初期情况说明：**

**“您好！请问是***的家长***吗？我们是中国疾病预防控制中心的访问员，我们想了解一下手足口病患者经济负担和生存质量的一些情况，用于国家制定疫苗免疫策略和补偿方案。您所说的话毫无疑问会被保密。您是否愿意在此基础之上继续接受此次访谈？”**

|  | **基本信息确认** | | | | | |  |
| --- | --- | --- | --- | --- | --- | --- | --- |
| 1 | 患者姓名是 吗？是 (620) 否 （名单记录姓名）（添加更正框） (630,639) | | | | | |  |
| 2 | 家属姓名是 吗？是 (640) 否 （名单记录姓名）（添加更正框） (650,659) | | | | | |  |
| 3 | 居住地址/工作单位是_________城市名称/农村名称（名单记录所在城市或农村）（添加更正框） (665,666)_ （城市需要问到具体城市名称，若是农村，则不用再追问具体地址） 是 (660) 否 | | | | | |  |
| 4 | 被访者**患儿**的类型：1）轻症； 2）重症； 3）死亡 (521) | | | | | |  |
| 5 | 患儿年龄：1）6个月~1岁； 2）1-2岁； 3）2-3岁； 4）3~4岁；5）4~5岁（5）(522) | | | | | |  |
| 6 | 采访员签字_______________ 采访日期________________采访长度_______________ | | | | | |  |
| **No.** | **问题及选项** | | | | | | **A** |
| 1 | 患儿患过几次手足口病？ 次(720,721)（0次以上）。(725) 您是否还能回忆起（）年（）月发病时的治疗费用吗？（提示受访者名单所列时间）如回答是则接受访谈，如否，结束访谈） | | | | | |  |
| 2 | 患者**记录当次**手足口病的主要诊断是（请参考病例，可多选）(730,769)/2   1. 单纯手足口病； 2）疱疹性咽峡炎；3）无菌性脑膜炎；4）脑炎；5）急性弛缓性麻痹；6）呼吸道感染；7）心肌炎；8）肺出血；9）肺水肿；10）心肺衰竭   11）其它 | | | | | |  |
| 3 | 孩子**记录当次**患手足口病期除第2题所列诊断选项以外是否同时患有其它基础性疾病？ (775) （不包括第2题选项中的“其他”，如回答是结束访谈） | | | | | |  |
| 4 |  | | | | | |  |
| 5 | 记录当次手足口病患儿患病时间共为几天？ 天(830,835)（0天以上）（保留一位小数点，由于担心患病好几个月，所以可设置为3位数，另外保留一位小数点 | | | | | |  |
| 6 | 记录当次手足口病的治疗类型：(836)  1）仅门诊治疗  2）先门诊治疗后住院治疗  3）门诊直接转住院治疗**（跳转Q9）** | | | | | |  |
| 7 | **记录当次患手足口病**，共去门/急诊的就诊次数为（ 次）(837,838) | | | | | |  |
| 8 | **项目** | **第一次门诊** | **第二次门诊** | | **第三次门诊** | **第四次门诊** |  |
|  | 1）医疗机构名称 | (920,923) | (924,927) | | (928,931) | (932,935) |  |
|  | 2）**这次患者**手足口病**门诊治疗总共花费了多少医药费用**？（元）(先让受访者回忆，回忆不出来给选项提示) （保留一位小数点） | 1）0-100 (940,946) (949)  2）101-300  3）301-500  4）501-800  5）801-1000  6）1K-2K  7) 2K-3K  8) 3K+ | 1）0-100 (950,956) (959)  2）101-300  3）301-500  4）501-800  5）801-1000  6）1K-2K  7) 2K-3K  8) 3K+ | | 1）0-100 (960,966) (969)  2）101-300  3）301-500  4）501-800  5）801-1000  6）1K-2K  7) 2K-3K  8) 3K+ | 1）0-100 (970,976) (979)  2）101-300  3）301-500  4）501-800  5）801-1000  6）1K-2K  7) 2K-3K  8) 3K+ |  |
|  | 其中**自付**了多少钱？(先让受访者回忆，回忆不出来给选项提示) （保留一位小数点） | 1）0-100 (1020,1026) (1029)  2）101-300  3）301-500  4）501-800  5）801-1000  6）1K-2K  7) 2K-3K  8) 3K+ | 1）0-100 (1030,1036) (1039)  2）101-300  3）301-500  4）501-800  5）801-1000  6）1K-2K  7) 2K-3K  8) 3K+ | | 1）0-100 (1040,1046) (1049)  2）101-300  3）301-500  4）501-800  5）801-1000  6）1K-2K  7) 2K-3K  8) 3K+ | 1）0-100 (1050,1056) (1059)  2）101-300  3）301-500  4）501-800  5）801-1000  6）1K-2K  7) 2K-3K  8) 3K+ |  |
|  | 3）**记录当次**患手足口病就诊的**往返交通费**（包括患者及其陪同人员）为多少元？ (先让受访者回忆，回忆不出来给选项提示) （保留一位小数点） | 1) <10 (1060,1066) (1069)  2) 10-30  3) 30-50  4) 50-100  5)100-200  6) 200+ | 1) <10 (1070,1076) (1079)  2) 10-30  3) 30-50  4) 50-100  5)100-200  6) 200+ | | 1) <10 (1120,1126) (1129)  2) 10-30  3) 30-50  4) 50-100  5)100-200  6) 200+ | 1) <10 (1130,1136) (1139)  2) 10-30  3) 30-50  4) 50-100  5)100-200  6) 200+ |  |
|  | 4）本次就诊**陪同人员有几**人？ （无，填写0；有，请填写**人数**） | 1140 人 | 1142 人 | | 1144 人 | 1146 人 |  |
|  | 5）陪同人员是否请假？ 陪护人员**请假天数**为多少天？**（请填写平均值）**（保留一位小数点）由于担心患病好几个月，所以可设置为3位数，另外保留一位小数点 | (1220,1225) 天 | (1230,1235) 天 | | (1240,1245) 天 | (1250,1255) 天 |  |
| 9 | 记录当次因手足口病治疗的住院次数：共计（ 次）(1240) | | | | | |  |
| 10 | **项目** | **第一次住院** | | | **第二次住院** | |  |
|  | 1）医院机构名称 | (1420,1423) | | | (1425,1428) | |  |
|  | 2）**住院天数**（保留一位小数点）由于担心患病好几个月，所以可设置为3位数，另外保留一位小数点 | (1430,1434) 天 | | | (1435,1439) 天 | |  |
|  | 3）**记录当次**患手足口病**总共花费了多少医药费用？**（元）(先让受访者回忆，回忆不出来给选项提示) （保留一位小数点） | 1) <1000 (1440,1447) (1449)  2) 1K-2K  3) 2K-3K  4) 3K-4K  5) 4K-5K  6) 5K-10K  6) 10K-20K  7) 20K-30K  8) 30K+ | | | 1) <1000 (1450,1457) (1459)  2) 1K-2K  3) 2K-3K  4) 3K-4K  5) 4K-5K  6) 5K-10K  6) 10K-20K  7) 20K-30K  8) 30K+ | |  |
|  | 其中**自付**了多少钱？（元）(先让受访者回忆，回忆不出来给选项提示) （保留一位小数点） | 1) <1000 (1460,1467) (1469)  2) 1K-2K  3) 2K-3K  4) 3K-4K  5) 4K-5K  6) 5K-10K  6) 10K-20K  7) 20K-30K  8) 30K+ | | | 1) <1000 (1470,1477) (1479)  2) 1K-2K  3) 2K-3K  4) 3K-4K  5) 4K-5K  6) 5K-10K  6) 10K-20K  7) 20K-30K  8) 30K+ | |  |
|  | 4）陪护患者家属**人数** | (1520,1521) 人 | | | (1525,1526) 人 | |  |
|  | 5）家属**陪护天数**为多少天？（平均值）（保留一位小数点）由于担心患病好几个月，所以可设置为3位数，另外保留一位小数点 | (1530,1534) 天 | | | (1535,1539) 天 | |  |
|  | 6）为照顾患者**家属是否请假？请假天数**为多少天？（无填写0，有填写天数）（保留一位小数点）由于担心患病好几个月，所以可设置为3位数，另外保留一位小数点 | (1540,1544) 天 | | | (1545,1549) 天 | |  |
|  | 7）住院期间陪护家属的**住宿费**为多少**元/天**？（无填写0，有填写金额）（保留一位小数点） | (1620,1626) 元/天 | | | (1630,1636) 元/天 | |  |
|  | 8）住院期间患者的**营养伙食费**为多少**元/天**？（无填写0，有填写金额）（保留一位小数点） | (1640,1646) 元/天 | | | (1650,1656) 元/天 | |  |
|  | 9）住院期间**陪护家属**的**伙食费**为多少**元/天**？（无填写0，有填写金额）（保留一位小数点） | (1660,1666) 元/天 | | | (1670,1676) 元/天 | |  |
|  | 10）住院期间是否雇佣护工？雇佣费用共计多少元？（无填写0，有填写金额）（保留一位小数点） | (1720,1727) 元 | | | (1730,1737) 元 | |  |
|  | 11）住院期间的往返交通费（包括患者及其陪同人员）共计多少元？(先让受访者回忆，回忆不出来给选项提示) （保留一位小数点） | 1) <100 (1820,1826) (1829)  2) 101-200  3) 201-300  4) 301-500  5) 501-1000  6) 1000+ | | | 1) <100 (1830,1836) (1839)  2) 101-200  3) 201-300  4) 301-500  5) 501-1000  6) 1000+ | |  |
| 11 | 本次疾病治疗过程中除门诊和住院外的药品外，是否给患者自行去**药店购买过药品？花费是多少元**？（无，填写0；有，填写金额）(先让受访者回忆，回忆不出来给选项提示) （保留一位小数点） | | | 1) <30 (1840,1846) (1849)  2) 31-50  3) 51-100  4)101-200  5) 201-500  6)501-1000  7) 1000+  8）无，0 | | |  |
| 12 | 本次患儿生病的全部过程中花费在是否给患者购买过**营养品、保健品、零食等费用**为多少元？（无填写0，有填写金额）(先让受访者回忆，回忆不出来给选项提示) （保留一位小数点） | | | 1) <100 (1850,1856) (1859)  2) 101-200  3) 201-300  4) 301-500  5) 501-1000  6) 1000+  7）无，0 | | |  |
|  |  | | |  | | |  |
| 13 | **行动**  患儿在手足口病患病期间，疾病对其行动能力是否有影响？ (2020)   1. 四处走动没有任何困难吗？ ❑ 2. 行动有些不方便吗？ ❑ 3. 不能下床活动吗？ ❑ 4. 发病年龄在1.5岁以下患儿无须作答❑ | | | | | |  |
| 14 | **日常活动**  患儿在手足口患病期间，是否能够进行日常活动诸如游戏，玩耍，画画等 (2021)   1. 进行他（她）的日常活动毫不困难 ❑ 2. 进行他（她）的日常活动有些困难 ❑ 3. 无法进行他（她）的日常活动 ❑ | | | | | |  |
| 15 | **疼痛/不适**  患儿在手足口患病期间，是否有疼痛或不适感觉？ (2022)   1. 没有疼痛或不舒服 ❑ 2. 中等的疼痛或不舒服 ❑ 3. 极其的疼痛或不舒服 ❑ | | | | | |  |
| 16 | **焦虑/沮丧**  患儿在手足口患病期间，是否出现心情低落、不活泼、发脾气、不爱说话、爱哭闹、没精神等心情不佳的表现？(备注：中度的就是有患儿有这种诸如哭闹的现象，但不严重。极其焦虑就是患儿生病期间哭闹等现象持续时间较长，比较难哄)。 (2023)   1. 没有焦虑或沮丧 ❑ 2. 中度焦虑或沮丧 ❑（包括轻度） 3. 极其的焦虑或沮丧 ❑ | | | | | |  |
| 17 | 为了帮助您反映患儿在手足口病患病期间的健康状况的好坏，我想请您在您的脑海中尝试画一个刻度尺，看上去有点像温度计。您能做到吗？刻度尺的顶端是100（一百）分，代表患儿最好的健康状况；刻度尺的底端是0（零）分，代表您患儿最差的健康状况。  现在，请您告诉我：您想在这个刻度尺上对您患儿患手足口病期间的健康状况打多少分？（ 分） (2040,2042) | | | | | |  |
|  | **感谢您抽出时间来回答这些问题。** | | | | | | |
