# Supplementary material for: Routine Pediatric Enterovirus 71 Vaccination in China: a Cost-Effectiveness Analysis
Source: PLoS Med. 2016 Mar 15;13(3):e1001975. doi: 10.1371/journal.pmed.1001975 (PMC4792415; doi:10.1371/journal.pmed.1001975)
Supplement: S4 Data — (DOCX) [file pmed.1001975.s004.docx]

**Explanation for data files:**

1. The file “S1_Data.csv” summarized the annual number of cases among children aged 6 months to 5 years old in both the virological surveillance data and the national surveillance databases. The number of cases were stratified by severity (i.e. mild, severe but not fatal, and fatal), province and serotype results if available (i.e. EV71, CA16, and OEV).

| **Column** | **Explanation** | **Notes** |
| --- | --- | --- |
| province | name of the province |  |
| prov_idx | administrative address code of the province | Chinese version available: <http://www.stats.gov.cn/tjsj/tjbz/xzqhdm/201401/t20140116_501070.html> |
| year | year | For virological surveillance records, each year started from the first Monday of the year. |

| **Virological surveillance data** | |
| --- | --- |
| **Column** | **Explanation** |
| year_negative | number of tests with negative results |
| year_ev71 | number of EV71 positive results |
| year_ca16 | number of CA16 positive results |
| year_other | number of OEV positive results |

| **National surveillance data** | |
| --- | --- |
| **Column** | **Explanation** |
| year_death | number of fatal HFMD cases in the national surveillance data |
| year_death_ev71 | number of EV71 positive fatal HFMD cases |
| year_death_ca16 | number of CA16 positive fatal HFMD cases |
| year_death_other | number of OEV positive fatal HFMD cases |
| year_severe | number of severe HFMD cases |
| year_severe_ev71 | number of EV71 positive severe HFMD cases |
| year_severe_ca16 | number of CA16 positive severe HFMD cases |
| year_severe_other | number of OEV positive severe HFMD cases |
| year_mild | number of mild HFMD cases |
| year_mild_ev71 | number of EV71 positive mild HFMD cases |
| year_mild_ca16 | number of CA16 positive mild HFMD cases |
| year_mild_other | number of OEV positive mild HFMD cases |

1. The file “S2_Data.csv” is the line list of the 1,787 EV71 positive subjects in the telephone survey. The data of the patient in the national surveillance database were combined into the survey linelist (i.e. from column “sex” to column “fbdate”).

| **Column** | **Explanation** | **Notes / Meaning of codes** |
| --- | --- | --- |
| sex |  | 1 = male  2 = female |
| age_by_fbdate | age of the patient upon the date of symptoms onset |  |
| zhongzheng | severe case or not | 1 = severe  0 = not severe |
| death | fatal case or not | 1 = fatal  0 = not fatal |
| diagnosis | lab test result | 0 = no lab test result available (including cases that were not tested and cases that were test negative)  1 = EV71 positive  2 = CA16 positive  3 = OEV positive |
| addcode | administrative address code of the patient’s residence (only at city district or county level) | Chinese version available: <http://www.stats.gov.cn/tjsj/tjbz/xzqhdm/201401/t20140116_501070.html> |
| fbyear | the year of symptoms onset |  |
| dur_disease | reported duration of illness |  |
| out_inpatient | outpatient or inpatient | 1 = outpatient  2 = outpatient first and admitted later  3 = inpatient |
| **Cost data of outpatient visits (max. 4 outpatient visits were recorded)** | | |
| num_outpatient | number of outpatient visits | -999 = no outpatient visit or unknown |
| outpatient_total_1 | the medical cost of the 1^st^ outpatient visit | -999 = no point estimate of the cost, no cost or unknown |
| outpatient_total_2 | the medical cost of the 2^nd^ outpatient visit | -999 = no point estimate of the cost, no cost or unknown |
| outpatient_total_3 | the medical cost of the 3^rd^ outpatient visit | -999 = no point estimate of the cost, no cost or unknown |
| outpatient_total_4 | the medical cost of the 4^th^ outpatient visit | -999 = no point estimate of the cost, no cost or unknown |
| outpat_categ_total_1 | the medical cost of the 1^st^ outpatient visit | 1 = 0-100  2 = 101-300  3 = 301-500  4 = 501-800  5 = 801-1000  6 = 1000-1999  7 = 2000-2999  8 = ≥ 3000  -999 = no cost or unknown |
| outpat_categ_total_2 | the medical cost of the 2^nd^ outpatient visit | 1 = 0-100  2 = 101-300  3 = 301-500  4 = 501-800  5 = 801-1000  6 = 1000-1999  7 = 2000-2999  8 = ≥ 3000  -999 = no cost or unknown |
| outpat_categ_total_3 | the medical cost of the 3^rd^ outpatient visit | 1 = 0-100  2 = 101-300  3 = 301-500  4 = 501-800  5 = 801-1000  6 = 1000-1999  7 = 2000-2999  8 = ≥ 3000  -999 = no cost or unknown |
| outpat_categ_total_4 | the medical cost of the 4^th^ outpatient visit | 1 = 0-100  2 = 101-300  3 = 301-500  4 = 501-800  5 = 801-1000  6 = 1000-1999  7 = 2000-2999  8 = ≥ 3000  -999 = no cost or unknown |
| outpat_trans_1 | the transportation cost of the 1st outpatient visit | -999 = no point estimate of the cost, no cost or unknown |
| outpat_trans_2 | the transportation cost of the 2nd outpatient visit | -999 = no point estimate of the cost, no cost or unknown |
| outpat_trans_3 | the transportation cost of the 3rd outpatient visit | -999 = no point estimate of the cost, no cost or unknown |
| outpat_trans_4 | the transportation cost of the 4th outpatient visit | -999 = no point estimate of the cost, no cost or unknown |
| outpat_categ_trans_1 | the transportation cost of the 1st outpatient visit | 1 = 0-9  2 = 10-30  3 = 30-50  4 = 50-99  5 = 100-199  6 = ≥ 200  -999 = no cost or unknown |
| outpat_categ_trans_2 | the transportation cost of the 2nd outpatient visit | 1 = 0-9  2 = 10-30  3 = 30-50  4 = 50-99  5 = 100-199  6 = ≥ 200  -999 = no cost or unknown |
| outpat_categ_trans_3 | the transportation cost of the 3rd outpatient visit | 1 = 0-9  2 = 10-30  3 = 30-50  4 = 50-99  5 = 100-199  6 = ≥ 200  -999 = no cost or unknown |
| outpat_categ_trans_4 | the transportation cost of the 4th outpatient visit | 1 = 0-9  2 = 10-30  3 = 30-50  4 = 50-99  5 = 100-199  6 = ≥ 200  -999 = no cost or unknown |
| outpat_trans_driving_1 | the driving cost of the 1st outpatient visit | -999 = no cost or unknown |
| outpat_trans_driving_2 | the driving cost of the 2nd outpatient visit | -999 = no cost or unknown |
| outpat_trans_driving_3 | the driving cost of the 3rd outpatient visit | -999 = no cost or unknown |
| outpat_trans_driving_4 | the driving cost of the 4th outpatient visit | -999 = no cost or unknown |
| outpat_num_accompany_1 | the number of accompanying persons of the 1^st^ outpatient visit | -999 = no accompanying person or unknown |
| outpat_num_accompany_2 | the number of accompanying persons of the 2^nd^ outpatient visit | -999 = no accompanying person or unknown |
| outpat_num_accompany_3 | the number of accompanying persons of the 3^rd^ outpatient visit | -999 = no accompanying person or unknown |
| outpat_num_accompany_4 | the number of accompanying persons of the 4^th^ outpatient visit | -999 = no accompanying person or unknown |
| outpat_num_leave_day_1 | the number of work days off of the 1^st^ outpatient visit | -999 = no work days off or unknown |
| outpat_num_leave_day_2 | the number of work days off of the 2^nd^ outpatient visit | -999 = no work days off or unknown |
| outpat_num_leave_day_3 | the number of work days off of the 3^rd^ outpatient visit | -999 = no work days off or unknown |
| outpat_num_leave_day_4 | the number of work days off of the 4^th^ outpatient visit | -999 = no work days off or unknown |
| **Cost data of inpatient visits (max. 2 inpatient visits were recorded)** | | |
| num_inpatient | number of inpatient visits | -999 = no inpatient visit or unknown |
| num_hosp_days_1 | number of days hospitalized of the 1^st^ inpatient visit | -999 = no inpatient visit or unknown |
| num_hosp_days_2 | number of days hospitalized of the 2^nd^ inpatient visit | -999 = no inpatient visit or unknown |
| inpatient_total_1 | the medical cost of the 1^st^ inpatient visit | -999 = no point estimate of the cost, no cost or unknown |
| inpatient_total_2 | the medical cost of the 2^nd^ inpatient visit | -999 = no point estimate of the cost, no cost or unknown |
| inpat_categ_total_1 | the medical cost of the 1^st^ outpatient visit | 1 = <1000  2 = 1001-1999  3 = 2000-2999  4 = 3000-3999  5 = 4000-4999  6 = 5000-9999  7 = 10000-19999  8 = 20000-29999  9 = ≥ 30000  -999 = no cost or unknown |
| inpat_categ_total_2 | the medical cost of the 2^nd^ outpatient visit | 1 = <1000  2 = 1001-1999  3 = 2000-2999  4 = 3000-3999  5 = 4000-4999  6 = 5000-9999  7 = 10000-19999  8 = 20000-29999  9 = ≥ 30000  -999 = no cost or unknown |
| inpat_num_accompany_1 | the number of accompanying persons of the 1^st^ inpatient visit | -999 = no accompanying person or unknown |
| inpat_num_accompany_2 | the number of accompanying persons of the 2^nd^ inpatient visit | -999 = no accompanying person or unknown |
| inpat_num_accompany_day_1 | the average number of accompanying days per person of the 1^st^ inpatient visit | -999 = no accompanying days or unknown |
| inpat_num_accompany_day_2 | the average number of accompanying days per person of the 2^nd^ inpatient visit | -999 = no accompanying days or unknown |
| inpat_num_leave_day_1 | the number of work days off of the 1st inpatient visit | -999 = no work days off or unknown |
| inpat_num_leave_day_2 | the number of work days off of the 2nd inpatient visit | -999 = no work days off or unknown |
| inpat_accommodation_1 | the accommodation cost of accompanying persons per day of the 1^st^ inpatient visit | -999 = no cost or unknown |
| inpat_accommodation_2 | the accommodation cost of accompanying persons per day of the 2^nd^ inpatient visit | -999 = no cost or unknown |
| inpat_nutrition_1 | the nutrition cost of the patient of the 1^st^ inpatient visit per day | -999 = no cost or unknown |
| inpat_nutrition_2 | the nutrition cost of the patient of the 2^nd^ inpatient visit per day | -999 = no cost or unknown |
| inpat_nut_takeaway_1 | the nutrition cost of the patient of the 1st inpatient visit (take-away from home) per day | -999 = no cost or unknown |
| inpat_nut_takeaway_2 | the nutrition cost of the patient of the 2nd inpatient visit (take-away from home) per day | -999 = no cost or unknown |
| inpat_nut_accomp_1 | the food cost of the accompanying persons of the 1st inpatient visit per day | -999 = no cost or unknown |
| inpat_nut_accomp_2 | the food cost of the accompanying persons of the 2nd inpatient visit per day | -999 = no cost or unknown |
| inpat_nut_accomp_takeaway_1 | the food cost of the accompanying persons of the 1st inpatient visit (take-away from home) per day | -999 = no cost or unknown |
| inpat_nut_accomp_takeaway_2 | the food cost of the accompanying persons of the 2nd inpatient visit (take-away from home) per day | -999 = no cost or unknown |
| inpat_helper_1 | the cost of helpers of the 1st inpatient visit | -999 = no cost or unknown |
| inpat_helper_2 | the cost of helpers of the 2nd inpatient visit | -999 = no cost or unknown |
| inpat_trans_1 | the transportation cost of the 1st inpatient visit | -999 = no point estimate of the cost, no cost or unknown |
| inpat_trans_2 | the transportation cost of the 2nd inpatient visit | -999 = no point estimate of the cost, no cost or unknown |
| inpat_categ_trans_1 | the transportation cost of the 1st inpatient visit | 1 = 0-99  2 = 101-200  3 = 201-300  4 = 301-500  5 = 501-1000  6 = ≥ 1000  -999 = no cost or unknown |
| inpat_categ_trans_2 | the transportation cost of the 2nd inpatient visit | 1 = 0-99  2 = 101-200  3 = 201-300  4 = 301-500  5 = 501-1000  6 = ≥ 1000  -999 = no cost or unknown |
| inpat_trans_driving_1 | the driving cost of the 1st inpatient visit | -999 = no cost or unknown |
| inpat_trans_driving_2 | the driving cost of the 2nd inpatient visit | -999 = no cost or unknown |
| inpat_drug | the cost of drugs bought outside the hospital | -999 = no point estimate of the cost, no cost or unknown |
| inpat_drug_categ | the cost of drugs bought outside the hospital | 1 = 0-29  2 = 31-50  3 = 51-100  4 = 101-200  5 = 201-500  6 = 501-1000  7 = ≥ 1000  -999 = no cost or unknown |
| inpat_snacks | the cost of snacks | -999 = no point estimate of the cost, no cost or unknown |
| inpat_snacks_categ | the cost of snacks | 1 = 0-99  2 = 101-200  3 = 201-300  4 = 301-500  5 = 501-1000  6 = ≥ 1000  -999 = no cost or unknown |
| **EQ-5D data** | | |
| motion | EQ-5D mobility dimension | 1 = no problem in walking about  2 = some problem in walking about  3 = confined to bed  4 = N/A for patients under 1.5 years old |
| dailyact | EQ-5D usual activity dimension | 1 = no problem  2 = some problem  3 = unable to perform usual activities |
| physicomfort | EQ-5D pain/discomfort dimension | 1 = no pain or discomfort  2 = moderate pain or discomfort  3 = extreme pain or discomfort |
| psycomfort | EQ-5D anxiety/depression dimension | 1 = not anxious or depressed  2 = moderately anxious or depressed  3 = extremely anxious or depressed |
| overhealth | EQ-5D VAS scale |  |
