# Supplementary material for: Routine Pediatric Enterovirus 71 Vaccination in China: a Cost-Effectiveness Analysis
Source: PLoS Med. 2016 Mar 15;13(3):e1001975. doi: 10.1371/journal.pmed.1001975 (PMC4792415; doi:10.1371/journal.pmed.1001975)
Supplement: S1 Table — (DOCX) [file pmed.1001975.s012.docx]

| **Province** | **Percentage of EV71-HFMD among severe/fatal HFMD cases** | | | | | | | | | | | | | | | | | | |
| --- | --- | --- | --- | --- | --- | --- | --- | --- | --- | --- | --- | --- | --- | --- | --- | --- | --- | --- | --- |
|  | **A** | **B** | **C** | **D** | **E** | **F** | **G** | **H** | **I** | **J** | **K** | **L** | **M** | **N** | **O** | **P** | **Q** | **R** | **S** |
| Anhui | 58% | 58% | 58% | 87% | 87% | 87% | 92% | 92% | 92% | 55% | 55% | 55% | 87% | 87% | 92% | 92% | 92% | 88% | 88% |
| Beijing | 32% | 32% | 32% | 58% | 58% | 58% | 75% | 75% | 75% | 17% | 17% | 17% | 60% | 60% | 86% | 86% | 86% | 56% | 56% |
| Chongqing | 55% | 55% | 55% | 73% | 73% | 73% | 80% | 80% | 80% | 55% | 55% | 55% | 73% | 73% | 80% | 80% | 80% | 73% | 73% |
| Fujian | 52% | 52% | 52% | 76% | 76% | 76% | 83% | 83% | 83% | 50% | 50% | 50% | 77% | 77% | 84% | 84% | 84% | 74% | 74% |
| Gansu | 58% | 58% | 58% | 79% | 79% | 79% | 85% | 85% | 85% | 53% | 53% | 53% | 79% | 79% | 86% | 86% | 86% | 79% | 79% |
| Guangdong | 63% | 63% | 63% | 78% | 78% | 78% | 82% | 82% | 82% | 63% | 63% | 63% | 78% | 78% | 82% | 82% | 82% | 78% | 78% |
| Guangxi | 54% | 54% | 54% | 72% | 72% | 72% | 80% | 80% | 80% | 49% | 49% | 49% | 73% | 73% | 82% | 82% | 82% | 70% | 70% |
| Guizhou | 34% | 34% | 34% | 58% | 58% | 58% | 75% | 75% | 75% | 34% | 34% | 34% | 58% | 58% | 75% | 75% | 75% | 55% | 55% |
| Hainan | 29% | 29% | 29% | 80% | 80% | 80% | 93% | 93% | 93% | 17% | 17% | 17% | 80% | 80% | 96% | 96% | 96% | 78% | 78% |
| Hebei | 62% | 62% | 62% | 81% | 81% | 81% | 86% | 86% | 86% | 62% | 62% | 62% | 81% | 81% | 86% | 86% | 86% | 81% | 81% |
| Heilongjiang | 63% | 63% | 63% | 87% | 87% | 87% | 91% | 91% | 91% | 63% | 63% | 63% | 87% | 87% | 91% | 91% | 91% | 85% | 85% |
| Henan | 60% | 60% | 60% | 82% | 82% | 82% | 87% | 87% | 87% | 42% | 42% | 42% | 83% | 83% | 91% | 91% | 91% | 82% | 82% |
| Hubei | 66% | 66% | 66% | 84% | 84% | 84% | 87% | 87% | 87% | 66% | 66% | 66% | 84% | 84% | 87% | 87% | 87% | 84% | 84% |
| Hunan | 46% | 46% | 46% | 71% | 71% | 71% | 81% | 81% | 81% | 39% | 39% | 39% | 72% | 72% | 84% | 84% | 84% | 70% | 70% |
| Inner Mongolia | 42% | 42% | 42% | 79% | 79% | 79% | 89% | 89% | 89% | 42% | 42% | 42% | 79% | 79% | 89% | 89% | 89% | 79% | 79% |
| Jiangsu | 43% | 43% | 43% | 72% | 72% | 72% | 84% | 84% | 84% | 38% | 38% | 38% | 73% | 73% | 86% | 86% | 86% | 69% | 69% |
| Jiangxi | 58% | 58% | 58% | 81% | 81% | 81% | 87% | 87% | 87% | 53% | 53% | 53% | 81% | 81% | 88% | 88% | 88% | 81% | 81% |
| Jilin | 54% | 54% | 54% | 76% | 76% | 76% | 82% | 82% | 82% | 50% | 50% | 50% | 76% | 76% | 83% | 83% | 83% | 75% | 75% |
| Liaoning | 48% | 48% | 48% | 65% | 65% | 65% | 71% | 71% | 71% | 39% | 39% | 39% | 64% | 64% | 77% | 77% | 77% | 62% | 62% |
| Ningxia | 53% | 53% | 53% | 84% | 84% | 84% | 91% | 91% | 91% | 39% | 39% | 39% | 83% | 83% | 93% | 93% | 93% | 85% | 85% |
| Qinghai | 100% | 100% | 100% | 100% | 100% | 100% | 100% | 100% | 100% | 100% | 100% | 100% | 100% | 100% | 100% | 100% | 100% | 100% | 100% |
| Shaanxi | 45% | 45% | 45% | 69% | 69% | 69% | 79% | 79% | 79% | 39% | 39% | 39% | 70% | 70% | 82% | 82% | 82% | 67% | 67% |
| Shandong | 45% | 45% | 45% | 70% | 70% | 70% | 81% | 81% | 81% | 24% | 24% | 24% | 71% | 71% | 90% | 90% | 90% | 69% | 69% |
| Shanghai | 83% | 83% | 83% | 94% | 94% | 94% | 95% | 95% | 95% | 83% | 83% | 83% | 94% | 94% | 95% | 95% | 95% | 94% | 94% |
| Shanxi | 63% | 63% | 63% | 86% | 86% | 86% | 89% | 89% | 89% | 24% | 24% | 24% | 88% | 88% | 96% | 96% | 96% | 86% | 86% |
| Sichuan | 48% | 48% | 48% | 66% | 66% | 66% | 75% | 75% | 75% | 48% | 48% | 48% | 66% | 66% | 75% | 75% | 75% | 65% | 65% |
| Tianjin | 16% | 16% | 16% | 79% | 79% | 79% | 86% | 86% | 86% | 12% | 12% | 12% | 91% | 91% | 97% | 97% | 97% | 82% | 82% |
| Tibet | 14% | 14% | 14% | 100% | 100% | 100% | 100% | 100% | 100% | 14% | 14% | 14% | 100% | 100% | 100% | 100% | 100% | 100% | 100% |
| Xinjiang | 87% | 87% | 87% | 95% | 95% | 95% | 95% | 95% | 95% | 84% | 84% | 84% | 93% | 93% | 95% | 95% | 95% | 94% | 94% |
| Yunnan | 52% | 52% | 52% | 84% | 84% | 84% | 90% | 90% | 90% | 39% | 39% | 39% | 84% | 84% | 92% | 92% | 92% | 83% | 83% |
| Zhejiang | 80% | 80% | 80% | 83% | 83% | 83% | 85% | 85% | 85% | 80% | 80% | 80% | 83% | 83% | 85% | 85% | 85% | 84% | 84% |

**S1 Table. The percentage of EV71-HFMD cases among all severe/fatal HFMD cases in each province in each of the 19 test-negative scenarios.**
