# Supplementary material for: Routine Pediatric Enterovirus 71 Vaccination in China: a Cost-Effectiveness Analysis
Source: PLoS Med. 2016 Mar 15;13(3):e1001975. doi: 10.1371/journal.pmed.1001975 (PMC4792415; doi:10.1371/journal.pmed.1001975)
Supplement: S2 Table — (DOCX) [file pmed.1001975.s013.docx]

| **Province** | **Percentage of EV71-HFMD among mild HFMD cases** | | | | | | | | | | | | | | | | | | |
| --- | --- | --- | --- | --- | --- | --- | --- | --- | --- | --- | --- | --- | --- | --- | --- | --- | --- | --- | --- |
|  | **A** | **B** | **C** | **D** | **E** | **F** | **G** | **H** | **I** | **J** | **K** | **L** | **M** | **N** | **O** | **P** | **Q** | **R** | **S** |
| Anhui | 27% | 51% | 74% | 27% | 51% | 74% | 27% | 51% | 74% | 27% | 51% | 74% | 27% | 74% | 27% | 51% | 74% | 26% | 75% |
| Beijing | 19% | 36% | 63% | 19% | 36% | 63% | 19% | 36% | 63% | 21% | 35% | 61% | 21% | 61% | 21% | 35% | 61% | 19% | 65% |
| Chongqing | 20% | 30% | 54% | 20% | 30% | 54% | 20% | 30% | 54% | 20% | 30% | 54% | 20% | 54% | 20% | 30% | 54% | 20% | 54% |
| Fujian | 28% | 44% | 63% | 28% | 44% | 63% | 28% | 44% | 63% | 28% | 44% | 63% | 28% | 63% | 28% | 44% | 63% | 27% | 64% |
| Gansu | 22% | 36% | 61% | 22% | 36% | 61% | 22% | 36% | 61% | 22% | 36% | 61% | 22% | 61% | 22% | 36% | 61% | 22% | 62% |
| Guangdong | 21% | 28% | 45% | 21% | 28% | 45% | 21% | 28% | 45% | 21% | 28% | 45% | 21% | 45% | 21% | 28% | 45% | 20% | 46% |
| Guangxi | 19% | 25% | 45% | 19% | 25% | 45% | 19% | 25% | 45% | 20% | 24% | 43% | 20% | 43% | 20% | 24% | 43% | 18% | 48% |
| Guizhou | 16% | 32% | 66% | 16% | 32% | 66% | 16% | 32% | 66% | 16% | 32% | 66% | 16% | 66% | 16% | 32% | 66% | 15% | 68% |
| Hainan | 10% | 23% | 67% | 10% | 23% | 67% | 10% | 23% | 67% | 11% | 22% | 62% | 11% | 62% | 11% | 22% | 62% | 9% | 71% |
| Hebei | 31% | 50% | 69% | 31% | 50% | 69% | 31% | 50% | 69% | 31% | 50% | 69% | 31% | 69% | 31% | 50% | 69% | 31% | 70% |
| Heilongjiang | 25% | 49% | 75% | 25% | 49% | 75% | 25% | 49% | 75% | 25% | 49% | 75% | 25% | 75% | 25% | 49% | 75% | 25% | 75% |
| Henan | 34% | 48% | 63% | 34% | 48% | 63% | 34% | 48% | 63% | 43% | 46% | 53% | 43% | 53% | 43% | 46% | 53% | 30% | 67% |
| Hubei | 50% | 72% | 81% | 50% | 72% | 81% | 50% | 72% | 81% | 50% | 72% | 81% | 50% | 81% | 50% | 72% | 81% | 50% | 81% |
| Hunan | 27% | 43% | 65% | 27% | 43% | 65% | 27% | 43% | 65% | 28% | 42% | 63% | 28% | 63% | 28% | 42% | 63% | 26% | 66% |
| Inner Mongolia | 12% | 40% | 81% | 12% | 40% | 81% | 12% | 40% | 81% | 12% | 40% | 81% | 12% | 81% | 12% | 40% | 81% | 12% | 81% |
| Jiangsu | 22% | 37% | 63% | 22% | 37% | 63% | 22% | 37% | 63% | 22% | 37% | 62% | 22% | 62% | 22% | 37% | 62% | 20% | 65% |
| Jiangxi | 21% | 33% | 58% | 21% | 33% | 58% | 21% | 33% | 58% | 21% | 32% | 58% | 21% | 58% | 21% | 32% | 58% | 20% | 60% |
| Jilin | 19% | 30% | 56% | 19% | 30% | 56% | 19% | 30% | 56% | 19% | 30% | 56% | 19% | 56% | 19% | 30% | 56% | 18% | 57% |
| Liaoning | 18% | 26% | 47% | 18% | 26% | 47% | 18% | 26% | 47% | 19% | 25% | 46% | 19% | 46% | 19% | 25% | 46% | 18% | 48% |
| Ningxia | 19% | 31% | 58% | 19% | 31% | 58% | 19% | 31% | 58% | 19% | 31% | 57% | 19% | 57% | 19% | 31% | 57% | 19% | 58% |
| Qinghai | 30% | 57% | 81% | 30% | 57% | 81% | 30% | 57% | 81% | 30% | 57% | 81% | 30% | 81% | 30% | 57% | 81% | 30% | 81% |
| Shaanxi | 18% | 32% | 62% | 18% | 32% | 62% | 18% | 32% | 62% | 19% | 31% | 60% | 19% | 60% | 19% | 31% | 60% | 16% | 66% |
| Shandong | 27% | 37% | 56% | 27% | 37% | 56% | 27% | 37% | 56% | 29% | 37% | 51% | 29% | 51% | 29% | 37% | 51% | 26% | 57% |
| Shanghai | 26% | 39% | 58% | 26% | 39% | 58% | 26% | 39% | 58% | 26% | 39% | 58% | 26% | 58% | 26% | 39% | 58% | 26% | 59% |
| Shanxi | 23% | 33% | 55% | 23% | 33% | 55% | 23% | 33% | 55% | 25% | 33% | 51% | 25% | 51% | 25% | 33% | 51% | 22% | 56% |
| Sichuan | 21% | 38% | 66% | 21% | 38% | 66% | 21% | 38% | 66% | 21% | 38% | 66% | 21% | 66% | 21% | 38% | 66% | 21% | 67% |
| Tianjin | 29% | 37% | 51% | 29% | 37% | 51% | 29% | 37% | 51% | 29% | 37% | 50% | 29% | 50% | 29% | 37% | 50% | 29% | 51% |
| Tibet | 43% | 65% | 74% | 43% | 65% | 74% | 43% | 65% | 74% | 48% | 66% | 72% | 48% | 72% | 48% | 66% | 72% | 43% | 75% |
| Xinjiang | 32% | 38% | 48% | 32% | 38% | 48% | 32% | 38% | 48% | 32% | 38% | 48% | 32% | 48% | 32% | 38% | 48% | 32% | 48% |
| Yunnan | 23% | 42% | 68% | 23% | 42% | 68% | 23% | 42% | 68% | 24% | 42% | 66% | 24% | 66% | 24% | 42% | 66% | 22% | 69% |
| Zhejiang | 32% | 38% | 52% | 32% | 38% | 52% | 32% | 38% | 52% | 32% | 38% | 52% | 32% | 52% | 32% | 38% | 52% | 32% | 52% |

**S2 Table. The percentage of EV71-HFMD cases among all mild HFMD cases in each province in each of the 19 test-negative scenarios.**
