# Supplementary material for: Routine Pediatric Enterovirus 71 Vaccination in China: a Cost-Effectiveness Analysis
Source: PLoS Med. 2016 Mar 15;13(3):e1001975. doi: 10.1371/journal.pmed.1001975 (PMC4792415; doi:10.1371/journal.pmed.1001975)
Supplement: S3 Table — (DOCX) [file pmed.1001975.s014.docx]

| **Province** | **Percentage of EV71-HFMD among severe/fatal test-negatives** | | | | | | | | | | | | | | | | | | |
| --- | --- | --- | --- | --- | --- | --- | --- | --- | --- | --- | --- | --- | --- | --- | --- | --- | --- | --- | --- |
|  | **A** | **B** | **C** | **D** | **E** | **F** | **G** | **H** | **I** | **J** | **K** | **L** | **M** | **N** | **O** | **P** | **Q** | **R** | **S** |
| Anhui | 0% | 0% | 0% | 86% | 86% | 86% | 100% | 100% | 100% | 0% | 0% | 0% | 85% | 85% | 100% | 100% | 100% | 0% | 0% |
| Beijing | 0% | 0% | 0% | 60% | 60% | 60% | 100% | 100% | 100% | 0% | 0% | 0% | 62% | 62% | 100% | 100% | 100% | 0% | 0% |
| Chongqing | 0% | 0% | 0% | 71% | 71% | 71% | 100% | 100% | 100% | 0% | 0% | 0% | 71% | 71% | 100% | 100% | 100% | 0% | 0% |
| Fujian | 0% | 0% | 0% | 77% | 77% | 77% | 100% | 100% | 100% | 0% | 0% | 0% | 78% | 78% | 100% | 100% | 100% | 0% | 0% |
| Gansu | 0% | 0% | 0% | 79% | 79% | 79% | 100% | 100% | 100% | 0% | 0% | 0% | 79% | 79% | 100% | 100% | 100% | 0% | 0% |
| Guangdong | 0% | 0% | 0% | 79% | 79% | 79% | 100% | 100% | 100% | 0% | 0% | 0% | 79% | 79% | 100% | 100% | 100% | 0% | 0% |
| Guangxi | 0% | 0% | 0% | 68% | 68% | 68% | 100% | 100% | 100% | 0% | 0% | 0% | 73% | 73% | 100% | 100% | 100% | 0% | 0% |
| Guizhou | 0% | 0% | 0% | 58% | 58% | 58% | 100% | 100% | 100% | 0% | 0% | 0% | 58% | 58% | 100% | 100% | 100% | 0% | 0% |
| Hainan | 0% | 0% | 0% | 79% | 79% | 79% | 100% | 100% | 100% | 0% | 0% | 0% | 80% | 80% | 100% | 100% | 100% | 0% | 0% |
| Hebei | 0% | 0% | 0% | 80% | 80% | 80% | 100% | 100% | 100% | 0% | 0% | 0% | 80% | 80% | 100% | 100% | 100% | 0% | 0% |
| Heilongjiang | 0% | 0% | 0% | 86% | 86% | 86% | 100% | 100% | 100% | 0% | 0% | 0% | 86% | 86% | 100% | 100% | 100% | 0% | 0% |
| Henan | 0% | 0% | 0% | 83% | 83% | 83% | 100% | 100% | 100% | 0% | 0% | 0% | 84% | 84% | 100% | 100% | 100% | 0% | 0% |
| Hubei | 0% | 0% | 0% | 89% | 89% | 89% | 100% | 100% | 100% | 0% | 0% | 0% | 89% | 89% | 100% | 100% | 100% | 0% | 0% |
| Hunan | 0% | 0% | 0% | 72% | 72% | 72% | 100% | 100% | 100% | 0% | 0% | 0% | 73% | 73% | 100% | 100% | 100% | 0% | 0% |
| Inner Mongolia | 0% | 0% | 0% | 79% | 79% | 79% | 100% | 100% | 100% | 0% | 0% | 0% | 79% | 79% | 100% | 100% | 100% | 0% | 0% |
| Jiangsu | 0% | 0% | 0% | 73% | 73% | 73% | 100% | 100% | 100% | 0% | 0% | 0% | 74% | 74% | 100% | 100% | 100% | 0% | 0% |
| Jiangxi | 0% | 0% | 0% | 82% | 82% | 82% | 100% | 100% | 100% | 0% | 0% | 0% | 82% | 82% | 100% | 100% | 100% | 0% | 0% |
| Jilin | 0% | 0% | 0% | 78% | 78% | 78% | 100% | 100% | 100% | 0% | 0% | 0% | 78% | 78% | 100% | 100% | 100% | 0% | 0% |
| Liaoning | 0% | 0% | 0% | 70% | 70% | 70% | 100% | 100% | 100% | 0% | 0% | 0% | 65% | 65% | 100% | 100% | 100% | 0% | 0% |
| Ningxia | 0% | 0% | 0% | 85% | 85% | 85% | 100% | 100% | 100% | 0% | 0% | 0% | 82% | 82% | 100% | 100% | 100% | 0% | 0% |
| Qinghai | 0% | 0% | 0% | 0% | 0% | 0% | 100% | 100% | 100% | 0% | 0% | 0% | 0% | 0% | 100% | 100% | 100% | 0% | 0% |
| Shaanxi | 0% | 0% | 0% | 72% | 72% | 72% | 100% | 100% | 100% | 0% | 0% | 0% | 72% | 72% | 100% | 100% | 100% | 0% | 0% |
| Shandong | 0% | 0% | 0% | 69% | 69% | 69% | 100% | 100% | 100% | 0% | 0% | 0% | 71% | 71% | 100% | 100% | 100% | 0% | 0% |
| Shanghai | 0% | 0% | 0% | 95% | 95% | 95% | 100% | 100% | 100% | 0% | 0% | 0% | 95% | 95% | 100% | 100% | 100% | 0% | 0% |
| Shanxi | 0% | 0% | 0% | 86% | 86% | 86% | 100% | 100% | 100% | 0% | 0% | 0% | 89% | 89% | 100% | 100% | 100% | 0% | 0% |
| Sichuan | 0% | 0% | 0% | 67% | 67% | 67% | 100% | 100% | 100% | 0% | 0% | 0% | 67% | 67% | 100% | 100% | 100% | 0% | 0% |
| Tianjin | 0% | 0% | 0% | 75% | 75% | 75% | 100% | 100% | 100% | 0% | 0% | 0% | 93% | 93% | 100% | 100% | 100% | 0% | 0% |
| Tibet | 0% | 0% | 0% | 100% | 100% | 100% | 100% | 100% | 100% | 0% | 0% | 0% | 100% | 100% | 100% | 100% | 100% | 0% | 0% |
| Xinjiang | 0% | 0% | 0% | 94% | 94% | 94% | 100% | 100% | 100% | 0% | 0% | 0% | 84% | 84% | 100% | 100% | 100% | 0% | 0% |
| Yunnan | 0% | 0% | 0% | 85% | 85% | 85% | 100% | 100% | 100% | 0% | 0% | 0% | 84% | 84% | 100% | 100% | 100% | 0% | 0% |
| Zhejiang | 0% | 0% | 0% | 61% | 61% | 61% | 100% | 100% | 100% | 0% | 0% | 0% | 61% | 61% | 100% | 100% | 100% | 0% | 0% |

**S3 Table. The percentage of EV71-HFMD cases among severe/fatal test-negative cases in each province in each of the 19 test-negative scenarios.**
