# Supplementary material for: Routine Pediatric Enterovirus 71 Vaccination in China: a Cost-Effectiveness Analysis
Source: PLoS Med. 2016 Mar 15;13(3):e1001975. doi: 10.1371/journal.pmed.1001975 (PMC4792415; doi:10.1371/journal.pmed.1001975)
Supplement: S5 Table — In the first column (i.e., societal perspective, 3% discount rate, willingness-to-pay threshold of 1 × GDPpc), the point estimates and 95% confidence intervals of EVCmax in scenarios B–S are compared to that in the base case (i.e., scenario A). For the remaining columns, the point estimates and 95% confidence intervals of EVCmax in each scenario are compared to their counterparts in the first column, i.e., EVCmax in scenario X of column Y was compared to EVCmax in scenario X of column 1. (DOCX) [file pmed.1001975.s016.docx]

|  | Societal perspective | | | | Excluding productivity loss of parents/caregivers | | | |
| --- | --- | --- | --- | --- | --- | --- | --- | --- |
|  | 3% discounting | | 6% discounting | | 3% discounting | | 6% discounting | |
|  | WTP 1 GDPpc | WTP 3 GDPpc | WTP 1 GDPpc | WTP 3 GDPpc | WTP 1 GDPpc | WTP 3 GDPpc | WTP 1 GDPpc | WTP 3 GDPpc |
| A  (Base case) | 0% [0%, 0%] | 81% [83%, 79%] | -18% [-19%, -18%] | 32% [33%, 31%] | -10% [-10%, -11%] | 71% [73%, 69%] | -28% [-29%, -28%] | 22% [23%, 21%] |
| B | 23% [22%, 24%] | 69% [72%, 67%] | -16% [-17%, -15%] | 28% [29%, 27%] | -12% [-12%, -13%] | 57% [60%, 54%] | -28% [-28%, -27%] | 16% [18%, 15%] |
| C | 57% [55%, 59%] | 58% [61%, 56%] | -14% [-14%, -13%] | 24% [26%, 23%] | -14% [-14%, -14%] | 44% [47%, 41%] | -27% [-27%, -27%] | 11% [13%, 10%] |
| D | 6% [7%, 6%] | 83% [85%, 81%] | -19% [-19%, -18%] | 32% [33%, 31%] | -10% [-10%, -10%] | 73% [75%, 71%] | -28% [-28%, -28%] | 23% [24%, 22%] |
| E | 29% [29%, 30%] | 71% [74%, 69%] | -16% [-17%, -16%] | 28% [30%, 27%] | -12% [-12%, -12%] | 59% [62%, 57%] | -28% [-28%, -27%] | 17% [19%, 16%] |
| F | 63% [62%, 65%] | 60% [63%, 58%] | -14% [-14%, -13%] | 25% [26%, 24%] | -14% [-13%, -14%] | 46% [49%, 44%] | -27% [-27%, -27%] | 12% [14%, 11%] |
| G | 8% [9%, 8%] | 83% [85%, 81%] | -19% [-19%, -18%] | 32% [33%, 31%] | -10% [-10%, -10%] | 73% [75%, 71%] | -28% [-28%, -28%] | 23% [24%, 22%] |
| H | 31% [31%, 32%] | 72% [74%, 69%] | -16% [-17%, -16%] | 29% [30%, 27%] | -12% [-11%, -12%] | 60% [63%, 58%] | -28% [-28%, -27%] | 17% [19%, 16%] |
| I | 65% [64%, 67%] | 61% [63%, 58%] | -14% [-15%, -14%] | 25% [27%, 24%] | -14% [-13%, -14%] | 47% [50%, 44%] | -27% [-27%, -27%] | 12% [14%, 11%] |
| J | 1% [1%, 1%] | 79% [81%, 76%] | -18% [-18%, -17%] | 31% [32%, 30%] | -11% [-11%, -11%] | 68% [71%, 66%] | -28% [-28%, -28%] | 21% [22%, 19%] |
| K | 20% [19%, 21%] | 69% [72%, 67%] | -16% [-16%, -15%] | 28% [29%, 27%] | -13% [-12%, -13%] | 56% [59%, 54%] | -28% [-28%, -27%] | 16% [17%, 15%] |
| L | 50% [48%, 52%] | 59% [62%, 57%] | -14% [-14%, -13%] | 25% [26%, 24%] | -14% [-14%, -15%] | 45% [48%, 42%] | -27% [-28%, -27%] | 11% [13%, 10%] |
| M | 10% [10%, 10%] | 80% [83%, 78%] | -18% [-19%, -18%] | 31% [33%, 30%] | -10% [-10%, -10%] | 70% [73%, 68%] | -28% [-28%, -28%] | 22% [23%, 20%] |
| N | 59% [58%, 61%] | 61% [64%, 59%] | -14% [-15%, -14%] | 25% [27%, 24%] | -14% [-13%, -14%] | 48% [51%, 45%] | -27% [-27%, -27%] | 13% [14%, 11%] |
| O | 13% [13%, 12%] | 81% [83%, 79%] | -18% [-19%, -18%] | 31% [33%, 30%] | -10% [-10%, -10%] | 71% [73%, 69%] | -28% [-28%, -27%] | 22% [23%, 21%] |
| P | 32% [31%, 32%] | 72% [74%, 69%] | -16% [-17%, -16%] | 28% [30%, 27%] | -12% [-11%, -12%] | 60% [63%, 58%] | -27% [-28%, -27%] | 17% [19%, 16%] |
| Q | 62% [60%, 63%] | 62% [65%, 59%] | -14% [-15%, -14%] | 26% [27%, 24%] | -13% [-13%, -14%] | 48% [51%, 46%] | -27% [-27%, -27%] | 13% [14%, 11%] |
| R | 4% [4%, 3%] | 84% [86%, 82%] | -19% [-20%, -19%] | 33% [34%, 32%] | -10% [-9%, -10%] | 75% [77%, 73%] | -28% [-29%, -28%] | 23% [25%, 22%] |
| S | 66% [64%, 68%] | 59% [62%, 57%] | -14% [-14%, -13%] | 25% [26%, 24%] | -14% [-13%, -14%] | 46% [49%, 43%] | -27% [-27%, -27%] | 12% [13%, 10%] |

**S5 Table. Changes in** *EVC_max_* **across the scenarios considered in Table 2.** In the first column (i.e. societal perspective, 3% discount rate, willingness-to-pay threshold of 1 × GDPpc), the point estimates and 95% confidence intervals of *EVC_max_* in scenarios B-S were compared to that in the base case (i.e. scenario A). For the remaining columns, the point estimates and 95% confidence intervals of *EVC_max_* in each scenario were compared to their counterparts in the first column, i.e. *EVC_max_* in scenario *X* of column *Y* was compared to *EVC_max_* in scenario *X* of column 1.
