# Supplementary material for: Routine Pediatric Enterovirus 71 Vaccination in China: a Cost-Effectiveness Analysis
Source: PLoS Med. 2016 Mar 15;13(3):e1001975. doi: 10.1371/journal.pmed.1001975 (PMC4792415; doi:10.1371/journal.pmed.1001975)
Supplement: S6 Table — (DOCX) [file pmed.1001975.s017.docx]

| **Region** | **Province composition** |
| --- | --- |
| Northeast | Heilongjiang, Jilin, Liaoning |
| East | Shandong, Jiangsu, Anhui, Jiangxi, Zhejiang, Fujian, Shanghai |
| South | Guangxi, Guangdong, Hainan |
| Central | Hubei, Hunan, Henan |
| North | Beijing, Tianjin, Hebei, Shanxi, Inner Mongolia |
| Northwest | Ningxia, Xinjiang, Qinghai, Shanxi, Gansu |
| Southwest | Sichuan, Yunnan, Guizhou, Xizang, Chongqing |

**S6 Table. Seven regions in China from which equal representation in the sample of our telephone survey was obtained.**
