# Supplementary material for: Routine Pediatric Enterovirus 71 Vaccination in China: a Cost-Effectiveness Analysis
Source: PLoS Med. 2016 Mar 15;13(3):e1001975. doi: 10.1371/journal.pmed.1001975 (PMC4792415; doi:10.1371/journal.pmed.1001975)
Supplement: S7 Table — (DOCX) [file pmed.1001975.s018.docx]

|  | | **Mild outpatient** | **Mild inpatient** | **Severe** | **Fatal** |
| --- | --- | --- | --- | --- | --- |
| **Total** | | 451 | 484 | 798 | 54 |
| **Gender** | **Male** | 297 (66%) | 318 (66%) | 536 (67%) | 30 (63%) |
|  | **Female** | 154 (34%) | 166 (34%) | 262 (33%) | 24 (37%) |
| **Urban/rural** | **Urban** | 186 (41%) | 292 (60%) | 443 (56%) | 26 (48%) |
|  | **Rural** | 265 (59%) | 192 (40%) | 355 (44%) | 28 (52%) |
| **Median age (2.5% - 97.5%)** | | 2.77 (0.88 – 4.87) | 2.39 (0.82 – 4.99) | 2.21 (0.98 – 4.58) | 2.03 (0.55 – 4.99) |
| **Duration of illness (2.5% - 97.5%)** | | 7.0 (2.0 – 15.0) | 7.0 (4.0 – 20.0) | 10.0 (5.0 – 30.0) | 6.0 (1.0 – 21.6) |
| **Geographical region** | **Northeast** | 65 (14%) | 45 (9%) | 140 (18%) | 3 (6%) |
|  | **East** | 69 (15%) | 89 (18%) | 11 (1%) | 10 (19%) |
|  | **South** | 57 (13%) | 126 (26%) | 61 (8%) | 2 (4%) |
|  | **Central** | 70 (16%) | 64 (13%) | 119 (15%) | 7 (13%) |
|  | **North** | 65 (14%) | 55 (11%) | 159 (20%) | 5 (9%) |
|  | **Northwest** | 64 (14%) | 54 (11%) | 164 (21%) | 16 (30%) |
|  | **Southwest** | 61 (14%) | 51 (11%) | 144 (18%) | 11 (20%) |

**S7 Table. Demographic characteristic and geographic distribution of 1,787 EV71-HFMD patients whose parents or caregivers were telephone survey participants**
