# Supplementary material for: Routine Pediatric Enterovirus 71 Vaccination in China: a Cost-Effectiveness Analysis
Source: PLoS Med. 2016 Mar 15;13(3):e1001975. doi: 10.1371/journal.pmed.1001975 (PMC4792415; doi:10.1371/journal.pmed.1001975)
Supplement: S8 Table — (DOCX) [file pmed.1001975.s019.docx]

|  | | **Mild outpatient** | **Mild inpatient** | **Severe** | **Fatal** |
| --- | --- | --- | --- | --- | --- |
| **Overall** | | 185 | 1400 | 3170 | 2738 |
| **Gender** | **Male** | 190 | 1417 | 3126 | 3326 |
|  | **Female** | 173 | 1369 | 3259 | 2003 |
| **Urban/rural** | **Urban** | 196 | 1284 | 3097 | 2338 |
|  | **Rural** | 168 | 1476 | 3228 | 3168 |
| **Age group** | **6 mo – 1 yr** | 208 | 1207 | 3133 | 3758 |
|  | **1 – 2 yrs** | 204 | 1517 | 3221 | 3359 |
|  | **2 – 3 yrs** | 193 | 1532 | 3318 | 2311 |
|  | **3 – 4 yrs** | 164 | 1211 | 2919 | 2000 |
|  | **4 – 5 yrs** | 154 | 1264 | 2985 | 1805 |
| **Geographical region** | **Northeast** | 143 | 1380 | 2999 | Not stratified |
|  | **East** | 239 | 1011 | 3592 |  |
|  | **South** | 200 | 2168 | 3085 |  |
|  | **Central** | 260 | 1145 | 3122 |  |
|  | **North** | 141 | 1669 | 4077 |  |
|  | **Northwest** | 143 | 1081 | 2655 |  |
|  | **Southwest** | 156 | 569 | 2964 |  |

**S8 Table. Costs for 1,787 EV71-HFMD patients whose parents or caregivers were telephone survey participants (mean, in US dollars)**
