# Supplementary material for: Routine Pediatric Enterovirus 71 Vaccination in China: a Cost-Effectiveness Analysis
Source: PLoS Med. 2016 Mar 15;13(3):e1001975. doi: 10.1371/journal.pmed.1001975 (PMC4792415; doi:10.1371/journal.pmed.1001975)
Supplement: S9 Table — (DOCX) [file pmed.1001975.s020.docx]

|  | | **Mild outpatient** | **Mild inpatient** | **Severe** | **Fatal** |
| --- | --- | --- | --- | --- | --- |
| **Overall** | | 0.0036 | 0.0082 | 0.0149 | 0.0111 |
| **Gender** | **Male** | 0.0033 | 0.0082 | 0.0143 | 0.0092 |
|  | **Female** | 0.0040 | 0.0082 | 0.0162 | 0.0136 |
| **Urban/rural** | **Urban** | 0.0034 | 0.0075 | 0.0134 | 0.0117 |
|  | **Rural** | 0.0038 | 0.0087 | 0.0162 | 0.0105 |
| **Age group** | **6 mo – 1 yr** | 0.0034 | 0.0064 | 0.0120 | 0.0114 |
|  | **1 – 2 yrs** | 0.0038 | 0.0081 | 0.0137 | 0.0078 |
|  | **2 – 3 yrs** | 0.0040 | 0.0087 | 0.0165 | 0.0093 |
|  | **3 – 4 yrs** | 0.0029 | 0.0087 | 0.0152 | 0.0176 |
|  | **4 – 5 yrs** | 0.0031 | 0.0078 | 0.0150 | 0.0155 |
| **Geographical region** | **Northeast** | 0.0034 | 0.0092 | 0.0141 | Not stratified |
|  | **East** | 0.0043 | 0.0064 | 0.0332 |  |
|  | **South** | 0.0031 | 0.0105 | 0.0135 |  |
|  | **Central** | 0.0038 | 0.0064 | 0.0143 |  |
|  | **North** | 0.0032 | 0.0100 | 0.0168 |  |
|  | **Northwest** | 0.0036 | 0.0079 | 0.0146 |  |
|  | **Southwest** | 0.0035 | 0.0055 | 0.0138 |  |

**S9 Table. QALY loss during illness for 1,787 EV71-HFMD patients whose parents or caregivers were telephone survey participants (mean)**
