# Supplementary material for: Routine Pediatric Enterovirus 71 Vaccination in China: a Cost-Effectiveness Analysis
Source: PLoS Med. 2016 Mar 15;13(3):e1001975. doi: 10.1371/journal.pmed.1001975 (PMC4792415; doi:10.1371/journal.pmed.1001975)
Supplement: S10 Table — (DOCX) [file pmed.1001975.s021.docx]

| **Province** | **Urban average annual income in 2013 (USD)** | **Rural average annual income in 2013 (USD)** | **Weighted average annual income in 2013 (USD)** | **Percentage of mild cases hospitalized** |
| --- | --- | --- | --- | --- |
| Beijing | 7265 | 2943 | 6668 | 0.4% |
| Tianjin | 5722 | 2542 | 5134 | 0.4% |
| Hebei | 3874 | 1461 | 2590 | 6.1% |
| Shanxi | 3854 | 1148 | 2535 | 0.8% |
| Inner Mongolia | 4329 | 1379 | 3083 | 3.7% |
| Liaoning | 4478 | 1689 | 3520 | 0.3% |
| Jilin | 3778 | 1544 | 2744 | 2.9% |
| Heilongjiang | 3394 | 1546 | 2598 | 3.2% |
| Shanghai | 7844 | 3144 | 7340 | 1.4% |
| Jiangsu | 5638 | 2182 | 4359 | 2.3% |
| Zhejiang | 6618 | 2585 | 5133 | 1.9% |
| Anhui | 4013 | 1300 | 2561 | 7.0% |
| Fujian | 5357 | 1795 | 3918 | 2.8% |
| Jiangxi | 3683 | 1409 | 2489 | 13.1% |
| Shandong | 4915 | 1704 | 3388 | 39.2% |
| Henan | 3801 | 1360 | 2396 | 34.9% |
| Hubei | 4041 | 1423 | 2824 | 5.5% |
| Hunan | 3955 | 1343 | 2561 | 2.5% |
| Guangdong | 5858 | 1873 | 4559 | 1.0% |
| Guangxi | 4016 | 1090 | 2364 | 4.0% |
| Hainan | 3999 | 1339 | 2711 | 2.2% |
| Chongqing | 4309 | 1337 | 3030 | 0.7% |
| Sichuan | 3834 | 1267 | 2385 | 5.4% |
| Guizhou | 3436 | 872 | 1806 | 2.9% |
| Yunnan | 3963 | 985 | 2156 | 2.3% |
| Tibet | 3620 | 1056 | 1639 | 1.8% |
| Shaanxi | 3869 | 1044 | 2457 | 6.9% |
| Gansu | 3233 | 820 | 1755 | 1.5% |
| Qinghai | 3551 | 994 | 2208 | 0.0% |
| Ningxia | 3814 | 1112 | 2482 | 0.1% |
| Xinjiang | 3593 | 1171 | 2236 | 1.6% |

**S10 Table. Average annual income in 2013 (urban income, rural income and income weighted by urban and rural population) and the percentage of mild HFMD cases that were inpatients in each of the 31 provinces.**
