# Supplementary material for: Routine Pediatric Enterovirus 71 Vaccination in China: a Cost-Effectiveness Analysis
Source: PLoS Med. 2016 Mar 15;13(3):e1001975. doi: 10.1371/journal.pmed.1001975 (PMC4792415; doi:10.1371/journal.pmed.1001975)
Supplement: S11 Table — A Kruskal–Wallis test was performed in each severity–region stratum. Multiple testing of the same hypothesis in the seven different regions was corrected for using false discovery rate control. Associations with p < 0.05 are highlighted. (DOCX) [file pmed.1001975.s022.docx]

| **Variable** | **Severity** | **Region** | **Cost p-value** | **QALY loss p-value** | **Adjusted cost p-value** | **Adjusted QALY loss p-value** |
| --- | --- | --- | --- | --- | --- | --- |
| Gender | Mild outpatient | Northeast | 0.962 | 0.887 | 0.962 | 0.977 |
|  |  | East | 0.224 | 0.922 | 0.523 | 0.977 |
|  |  | South | 0.219 | 0.411 | 0.523 | 0.873 |
|  |  | Central | 0.560 | 0.499 | 0.785 | 0.873 |
|  |  | North | 0.320 | 0.144 | 0.559 | 0.873 |
|  |  | Northwest | 0.774 | 0.977 | 0.903 | 0.977 |
|  |  | Southwest | 0.082 | 0.446 | 0.523 | 0.873 |
|  | Mild inpatient | Northeast | 0.009 | 0.073 | 0.062 | 0.512 |
|  |  | East | 0.868 | 0.709 | 0.868 | 0.832 |
|  |  | South | 0.150 | 0.836 | 0.325 | 0.836 |
|  |  | Central | 0.592 | 0.589 | 0.690 | 0.832 |
|  |  | North | 0.097 | 0.232 | 0.325 | 0.541 |
|  |  | Northwest | 0.186 | 0.223 | 0.325 | 0.541 |
|  |  | Southwest | 0.529 | 0.713 | 0.690 | 0.832 |
|  | Severe | Northeast | 0.051 | 0.127 | 0.177 | 0.320 |
|  |  | East | 0.100 | 0.714 | 0.192 | 0.823 |
|  |  | South | 0.452 | 0.823 | 0.501 | 0.823 |
|  |  | Central | 0.501 | 0.050 | 0.501 | 0.320 |
|  |  | North | 0.011 | 0.200 | 0.079 | 0.349 |
|  |  | Northwest | 0.110 | 0.137 | 0.192 | 0.320 |
|  |  | Southwest | 0.162 | 0.548 | 0.227 | 0.768 |
|  | Fatal | Nationwide | 0.076 | 0.116 | 0.076 | 0.116 |
| Age | Mild outpatient | Northeast | 0.572 | 0.210 | 0.672 | 0.446 |
|  |  | East | 0.445 | 0.415 | 0.672 | 0.582 |
|  |  | South | 0.072 | 0.076 | 0.271 | 0.276 |
|  |  | Central | 0.124 | 0.754 | 0.290 | 0.880 |
|  |  | North | 0.077 | 0.255 | 0.271 | 0.446 |
|  |  | Northwest | 0.868 | 0.079 | 0.868 | 0.276 |
|  |  | Southwest | 0.576 | 0.916 | 0.672 | 0.916 |
|  | Mild inpatient | Northeast | 0.925 | 0.574 | 0.925 | 0.850 |
|  |  | East | 0.108 | 0.752 | 0.318 | 0.850 |
|  |  | South | 0.437 | 0.386 | 0.612 | 0.850 |
|  |  | Central | 0.018 | 0.451 | 0.129 | 0.850 |
|  |  | North | 0.866 | 0.817 | 0.925 | 0.850 |
|  |  | Northwest | 0.277 | 0.850 | 0.485 | 0.850 |
|  |  | Southwest | 0.136 | 0.099 | 0.318 | 0.693 |
|  | Severe | Northeast | 0.462 | 0.025 | 0.992 | 0.178 |
|  |  | East | 0.604 | 0.204 | 0.992 | 0.475 |
|  |  | South | 0.156 | 0.633 | 0.545 | 0.948 |
|  |  | Central | 0.992 | 0.753 | 0.992 | 0.948 |
|  |  | North | 0.968 | 0.830 | 0.992 | 0.948 |
|  |  | Northwest | 0.131 | 0.948 | 0.545 | 0.948 |
|  |  | Southwest | 0.760 | 0.070 | 0.992 | 0.244 |
|  | Fatal | Nationwide | 0.855 | 0.674 | 0.855 | 0.674 |
| Urban residence status | Mild outpatient | Northeast | 0.133 | 0.058 | 0.464 | 0.175 |
|  |  | East | 0.757 | 0.079 | 0.922 | 0.175 |
|  |  | South | 0.909 | 0.100 | 0.922 | 0.175 |
|  |  | Central | 0.554 | 0.016 | 0.922 | 0.110 |
|  |  | North | 0.922 | 0.284 | 0.922 | 0.331 |
|  |  | Northwest | 0.109 | 0.284 | 0.464 | 0.331 |
|  |  | Southwest | 0.302 | 0.611 | 0.704 | 0.611 |
|  | Mild inpatient | Northeast | 0.007 | 0.852 | 0.050 | 0.852 |
|  |  | East | 0.625 | 0.348 | 0.724 | 0.590 |
|  |  | South | 0.177 | 0.652 | 0.457 | 0.761 |
|  |  | Central | 0.361 | 0.155 | 0.505 | 0.362 |
|  |  | North | 0.262 | 0.144 | 0.459 | 0.362 |
|  |  | Northwest | 0.724 | 0.421 | 0.724 | 0.590 |
|  |  | Southwest | 0.196 | 0.060 | 0.457 | 0.362 |
|  | Severe | Northeast | 0.642 | 0.820 | 0.642 | 0.952 |
|  |  | East | 0.100 | 0.271 | 0.234 | 0.949 |
|  |  | South | 0.558 | 0.918 | 0.642 | 0.952 |
|  |  | Central | 0.599 | 0.653 | 0.642 | 0.952 |
|  |  | North | 0.062 | 0.033 | 0.217 | 0.233 |
|  |  | Northwest | 0.016 | 0.952 | 0.114 | 0.952 |
|  |  | Southwest | 0.300 | 0.835 | 0.524 | 0.952 |
|  | Fatal | Nationwide | 0.213 | 0.294 | 0.213 | 0.294 |

**S11 Table. Association ofcosts and QALY loss with age, gender and urban residence status.** A Kruskal–Wallis test was performed in each severity-region stratum. Multiple testing of the same hypothesis in the 7 different regions was corrected for using false discovery rate control. Associations with *p* < 0.05 are highlighted.
