# Supplementary material for: Routine Pediatric Enterovirus 71 Vaccination in China: a Cost-Effectiveness Analysis
Source: PLoS Med. 2016 Mar 15;13(3):e1001975. doi: 10.1371/journal.pmed.1001975 (PMC4792415; doi:10.1371/journal.pmed.1001975)
Supplement: S12 Table — (DOCX) [file pmed.1001975.s023.docx]

|  | **Region** | ***M_Q_s,d_***  (10^-3^) | ***V_Q_s,d_* (**10^-7^) | **Societal perspective** | | | **Excluding productivity loss** | | |
| --- | --- | --- | --- | --- | --- | --- | --- | --- | --- |
|  |  |  |  | ***M_C_s,d_*** | ***V_C_s,d_* (**10^4^) | ***CV_C_s,d__Q_s,d_*** | ***M_C_s,d_*** | ***V_C_s,d_* (**10^4^) | ***CV_C_s,d__Q_s,d_*** |
| **Mild outpatients** | Northeast | 4.04 | 6.14 | 223 | 0.080 | 0.011 | 158 | 0.048 | 0.011 |
|  | East | 3.55 | 0.94 | 244 | 0.100 | 0.002 | 164 | 0.046 | 0.001 |
|  | South | 3.38 | 4.06 | 136 | 0.017 | 0.004 | 105 | 0.009 | 0.003 |
|  | Central | 3.02 | 1.57 | 134 | 0.029 | 0.001 | 86 | 0.016 | 0.000 |
|  | North | 2.92 | 1.83 | 188 | 0.107 | 0.000 | 131 | 0.059 | 0.000 |
|  | Northwest | 3.21 | 1.70 | 134 | 0.016 | 0.002 | 102 | 0.008 | 0.001 |
|  | Southwest | 3.27 | 2.34 | 147 | 0.053 | 0.002 | 121 | 0.042 | 0.002 |
| **Mild inpatients­­** | Northeast | 6.00 | 2.51 | 941 | 0.755 | 0.015 | 874 | 0.699 | 0.014 |
|  | East | 6.13 | 7.01 | 1088 | 1.294 | 0.038 | 953 | 1.186 | 0.036 |
|  | South | 7.56 | 11.27 | 1032 | 5.334 | 0.143 | 957 | 4.499 | 0.131 |
|  | Central | 9.56 | 31.10 | 1596 | 11.004 | 0.301 | 1509 | 10.519 | 0.290 |
|  | North | 9.97 | 6.00 | 2063 | 1.690 | 0.044 | 1955 | 1.615 | 0.044 |
|  | Northwest | 8.69 | 56.68 | 1304 | 7.354 | 0.106 | 1235 | 7.329 | 0.099 |
|  | Southwest | 5.18 | 2.26 | 538 | 0.315 | 0.014 | 517 | 0.289 | 0.013 |
| **Severe** | Northeast | 31.45 | 3604.7 | 3389 | 73.880 | 13.587 | 3278 | 70.423 | 13.611 |
|  | East | 13.67 | 11.47 | 2978 | 1.695 | 0.053 | 2771 | 1.516 | 0.047 |
|  | South | 13.98 | 16.07 | 2537 | 4.286 | 0.109 | 2426 | 4.121 | 0.100 |
|  | Central | 16.18 | 10.30 | 3932 | 10.882 | 0.139 | 3815 | 10.526 | 0.134 |
|  | North | 12.95 | 14.86 | 2957 | 5.199 | 0.105 | 2837 | 4.983 | 0.100 |
|  | Northwest | 13.44 | 8.43 | 2851 | 2.090 | 0.053 | 2725 | 2.010 | 0.051 |
|  | Southwest | 13.12 | 10.32 | 2823 | 2.146 | 0.072 | 2761 | 2.124 | 0.070 |
| **Fatal** | Nationwide | 30424 | 41.62 | 2449 | 25.859 | 0.292 | 2228 | 24.713 | 0.247 |

**S12 Table. Mean, variance and covariance of our survey data on cost and QALY loss per mild outpatient, mild inpatient, severe case and fatal case of EV71-HFMD in each region.**
