# Supplementary material for: Routine Pediatric Enterovirus 71 Vaccination in China: a Cost-Effectiveness Analysis
Source: PLoS Med. 2016 Mar 15;13(3):e1001975. doi: 10.1371/journal.pmed.1001975 (PMC4792415; doi:10.1371/journal.pmed.1001975)
Supplement: S13 Table — (DOCX) [file pmed.1001975.s024.docx]

| **Province** | **Region** | **Expected cost (USD)**  **Societal perspective** | | | **Expected cost (USD)**  **Excluding productivity loss** | | | **Expected QALY loss** | | |
| --- | --- | --- | --- | --- | --- | --- | --- | --- | --- | --- |
|  |  | **Fatal** | **Severe** | **Mild** | **Fatal** | **Severe** | **Mild** | **Fatal** | **Severe** | **Mild** |
| Beijing | North | 2449 | 2957 | 196 | 2228 | 2837 | 138 | 30.42 | 0.0130 | 0.0029 |
| Tianjin | North | 2449 | 2957 | 196 | 2228 | 2837 | 138 | 30.42 | 0.0130 | 0.0029 |
| Hebei | North | 2449 | 2957 | 304 | 2228 | 2837 | 243 | 30.42 | 0.0130 | 0.0034 |
| Shanxi | North | 2449 | 2957 | 204 | 2228 | 2837 | 147 | 30.42 | 0.0130 | 0.0030 |
| Inner Mongolia | North | 2449 | 2957 | 258 | 2228 | 2837 | 199 | 30.42 | 0.0130 | 0.0032 |
| Liaoning | Northeast | 2449 | 3389 | 225 | 2228 | 3278 | 160 | 30.42 | 0.0315 | 0.0040 |
| Jilin | Northeast | 2449 | 3389 | 243 | 2228 | 3278 | 178 | 30.42 | 0.0315 | 0.0041 |
| Heilongjiang | Northeast | 2449 | 3389 | 246 | 2228 | 3278 | 181 | 30.42 | 0.0315 | 0.0041 |
| Shanghai | East | 2449 | 2978 | 256 | 2228 | 2771 | 175 | 30.42 | 0.0137 | 0.0036 |
| Jiangsu | East | 2449 | 2978 | 263 | 2228 | 2771 | 182 | 30.42 | 0.0137 | 0.0036 |
| Zhejiang | East | 2449 | 2978 | 260 | 2228 | 2771 | 179 | 30.42 | 0.0137 | 0.0036 |
| Anhui | East | 2449 | 2978 | 303 | 2228 | 2771 | 219 | 30.42 | 0.0137 | 0.0037 |
| Fujian | East | 2449 | 2978 | 268 | 2228 | 2771 | 186 | 30.42 | 0.0137 | 0.0036 |
| Jiangxi | East | 2449 | 2978 | 355 | 2228 | 2771 | 268 | 30.42 | 0.0137 | 0.0039 |
| Shandong | East | 2449 | 2978 | 574 | 2228 | 2771 | 473 | 30.42 | 0.0137 | 0.0046 |
| Henan | Central | 2449 | 3932 | 643 | 2228 | 3815 | 583 | 30.42 | 0.0162 | 0.0053 |
| Hubei | Central | 2449 | 3932 | 214 | 2228 | 3815 | 164 | 30.42 | 0.0162 | 0.0034 |
| Hunan | Central | 2449 | 3932 | 170 | 2228 | 3815 | 121 | 30.42 | 0.0162 | 0.0032 |
| Guangdong | South | 2449 | 2537 | 144 | 2228 | 2426 | 114 | 30.42 | 0.0140 | 0.0034 |
| Guangxi | South | 2449 | 2537 | 171 | 2228 | 2426 | 139 | 30.42 | 0.0140 | 0.0035 |
| Hainan | South | 2449 | 2537 | 156 | 2228 | 2426 | 124 | 30.42 | 0.0140 | 0.0035 |
| Chongqing | Southwest | 2449 | 2823 | 150 | 2228 | 2761 | 124 | 30.42 | 0.0131 | 0.0033 |
| Sichuan | Southwest | 2449 | 2823 | 168 | 2228 | 2761 | 143 | 30.42 | 0.0131 | 0.0034 |
| Guizhou | Southwest | 2449 | 2823 | 159 | 2228 | 2761 | 133 | 30.42 | 0.0131 | 0.0033 |
| Yunnan | Southwest | 2449 | 2823 | 156 | 2228 | 2761 | 130 | 30.42 | 0.0131 | 0.0033 |
| Tibet | Southwest | 2449 | 2823 | 154 | 2228 | 2761 | 129 | 30.42 | 0.0131 | 0.0033 |
| Shaanxi | Northwest | 2449 | 2851 | 215 | 2228 | 2725 | 179 | 30.42 | 0.0134 | 0.0036 |
| Gansu | Northwest | 2449 | 2851 | 152 | 2228 | 2725 | 119 | 30.42 | 0.0134 | 0.0033 |
| Qinghai | Northwest | 2449 | 2851 | 134 | 2228 | 2725 | 102 | 30.42 | 0.0134 | 0.0032 |
| Ningxia | Northwest | 2449 | 2851 | 135 | 2228 | 2725 | 103 | 30.42 | 0.0134 | 0.0032 |
| Xinjiang | Northwest | 2449 | 2851 | 154 | 2228 | 2725 | 120 | 30.42 | 0.0134 | 0.0033 |

**S13 Table. Expected cost and QALY loss per case of mild, severe and fatal EV71-HFMD in each of the 31 provinces.**
