# Supplementary material for: Routine Pediatric Enterovirus 71 Vaccination in China: a Cost-Effectiveness Analysis
Source: PLoS Med. 2016 Mar 15;13(3):e1001975. doi: 10.1371/journal.pmed.1001975 (PMC4792415; doi:10.1371/journal.pmed.1001975)
Supplement: S14 Table — (DOCX) [file pmed.1001975.s025.docx]

| **County** | **Study period** | **Age range** | **No. of subjects in placebo group** | **No. of EV71-HFMD in placebo group (per-protocol; EV71 associated diseases)** | **EV71-HFMD incidence rate**  **(per 1000 person-year)** |
| --- | --- | --- | --- | --- | --- |
| Donghai, Jiangsu  [ Lancet 2013 ] | Jan 2012 – Mar 2013 | 6 – 35 months | 1438 (Appendix 6) | 5 (Appendix 12) | 3.5 (1.1-8.1) |
| Pizhou, Jiangsu  [ Lancet 2013 ] | Jan 2012 – Mar 2013 | 6 – 35 months | 2227 (Appendix 6) | 3 (Appendix 12) | 1.3 (0.3-3.9) |
| Baoying, Jiangsu  [ Lancet 2013 ] | Jan 2012 – Mar 2013 | 6 – 35 months | 1046 (Appendix 6) | 28 (Appendix 12) | 26.8 (17.9-38.5) |
| Chaoyang, Beijing  [ Lancet 2013 ] | Jan 2012 – Mar 2013 | 6 – 35 months | 414 (Appendix 6) | 5 (Appendix 12) | 12.1(3.9-28.0) |
| Ganyu, Jiangsu  [ Zhu NEJM 2014 ] | Jan 2012 – Mar 2013 | 6 – 35 months | 2323 (S7 Table) | 19 (S11 Table) | 8.2 (4.9-12.7) |
| Sheyang Jiangsu  [Zhu NEJM 2014 ] | Jan 2012 – Mar 2013 | 6 – 35 months | 1675 (S7 Table) | 63 (S11 Table) | 37.6 (29.0-47.9) |
| Taixing, Jiangsu  [ Zhu NEJM 2014 ] | Jan 2012 – Mar 2013 | 6 – 35 months | 1035 (S7 Table) | 19 (S11 Table) | 18.4 (11.1-28.5) |
| 7 contiguous counties, Guangxi  [ Li NEJM 2014 ] | Mar 2012 – Feb 2013 | 6 – 71 months | 5499 (S2 Table) | 145 (S2 Table) | 26.4 (22.3-31.0) |
| Aggregated | Jan 2012 –Mar 2013 | 6 – 71 months | 15657 | 287 | 18.3 (16.3-20.6) |

**S14 Table. Estimated incidence rate of EV71-HFMD in the study areas of the EV71 vaccine phase III trials.**
