# Supplementary material for: Routine Pediatric Enterovirus 71 Vaccination in China: a Cost-Effectiveness Analysis
Source: PLoS Med. 2016 Mar 15;13(3):e1001975. doi: 10.1371/journal.pmed.1001975 (PMC4792415; doi:10.1371/journal.pmed.1001975)
Supplement: S15 Table — (DOCX) [file pmed.1001975.s026.docx]

| **County** | **Population size (2010 census)** | **No. of HFMD cases, *K_X_*_,_*_T_*** | **No. of test-positives** | **No. of test-positives that were EV71** | **Percentage of HFMD cases that were EV71, *R_X,T_*** | **EV71-HFMD**  **incidence rate**  **(per 1000 person-year)** | **EV71-HFMD incidence rate in national surveillance/ EV71-HFMD incidence rate in vaccine trials** |
| --- | --- | --- | --- | --- | --- | --- | --- |
| Donghai, Jiangsu | 97273 | 281 | 21 | 5 | 0.24 (0.08-0.47) | 0.7 (0.3-1.3) | 0.20 (0.05-0.99) |
| Pizhou, Jiangsu | 140184 | 273 | 3 | 1 | 0.33 (0.01-0.91) | 0.6 (0.0-1.8) | 0.48 (0.00-10.0) |
| Baoying, Jiangsu | 30721 | 568 | 41 | 11 | 0.27 (0.14-0.43) | 5.0 (2.8-7.7) | 0.19 (0.09-0.33) |
| Chaoyang, Beijing | 118625 | 2504 | 123 | 40 | 0.33 (0.24-0.42) | 6.9 (5.2-8.7) | 0.58 (0.27-2.77) |
| Ganyu, Jiangsu | 78769 | 1629 | 35 | 4 | 0.11 (0.03-0.27) | 2.4 (0.7-5.5) | 0.28 (0.06-0.69) |
| Sheyang Jiangsu | 47424 | 402 | 21 | 16 | 0.76 (0.53-0.92) | 6.5 (4.6-7.7) | 0.17 (0.12-0.24) |
| Taixing, Jiangsu | 41092 | 550 | 26 | 4 | 0.15 (0.04-0.35) | 2.1 (0.7-4.5) | 0.11 (0.03-0.26) |
| 7 contiguous counties, Guangxi | 207696 | 17415 | 496 | 251 | 0.51 (0.46-0.55) | 42.4 (38.8-46.1) | 1.61 (1.35-1.95) |
| Aggregated | 761784 | 23622 | 766 | 332 | 0.43 (0.40-0.47) | 13.4 (12.4-14.5) | 0.74 (0.64-0.84) |

**S15 Table. Estimated incidence rate of EV71-HFMD cases in the national surveillance database in the study areas of the EV71 vaccine phase III trials**.
