# Supplementary material for: Routine Pediatric Enterovirus 71 Vaccination in China: a Cost-Effectiveness Analysis
Source: PLoS Med. 2016 Mar 15;13(3):e1001975. doi: 10.1371/journal.pmed.1001975 (PMC4792415; doi:10.1371/journal.pmed.1001975)
Supplement: S1 Text — (DOCX) [file pmed.1001975.s027.docx]

**Cost-effectiveness of routine pediatric enterovirus 71 vaccination in China**

Joseph T Wu, PhD^1†^, Mark Jit, PhD^2,3†^, Yaming Zheng, PhD^4†^, Kathy Leung, MPhil^1^, Weijia Xing, PhD^4^, Juan Yang, PhD^4^, Qiaohong Liao, MD^4^, Benjamin J Cowling, PhD^1^, Bingyi Yang, MSc^1^, Eric H Y Lau, PhD^1^, Saki Takahashi, ScM^5^, Jeremy J. Farrar, PhD^7^, Bryan T Grenfell, PhD^5,6^, Gabriel M. Leung, MD^1*^, Hongjie Yu, PhD^4*^

S1 Text: Model specifications and parameterization

[The model 2](#_Toc441336391)

[Uncertainty in test-negative cases 4](#_Toc441336392)

[Survey on household costs and quality of life detriment associated with EV71-HFMD 5](#_Toc441336393)

[Estimation of the unregistered proportion 8](#_Toc441336394)

[Indirect protection conferred by vaccination 9](#_Toc441336395)

[Under what conditions would we not vaccinate because a more cost-effective alternative intervention exists? 10](#_Toc441336396)

[Comparative CEA of the three vaccine candidates 12](#_Toc441336397)

[References 36](#_Toc441336398)

### The model

Let *Z_mild,p,t_*, *Z_severe,p,t_* and *Z_fatal,p,t_* be the number of mild, severe and fatal HFMD cases aged between 6 months and 5 years (60 months) registered by national HFMD surveillance in province *p* in year *t* = 2010, 2011, 2012, 2013. Let *R_mild,p,t_*, *R_severe,p,t_* and *R_fatal,p,t_* be the corresponding percentage of laboratory test results that were EV71. The risk of EV71-HFMD between age 6 months and 5 years in the model was estimated as

$$\begin{matrix} P_{mild}=\frac{\sum_{t=2010}^{2013} \sum_{p=1}^{31} Z_{mild,p,t}R_{mild,p,t}}{W} \\ P_{severe}=\frac{\sum_{t=2010}^{2013} \sum_{p=1}^{31} Z_{severe,p,t}R_{severe,p,t}}{W} \\ P_{fatal}=\frac{\sum_{t=2010}^{2013} \sum_{p=1}^{31} Z_{fatal,p,t}R_{fatal,p,t}}{W} \end{matrix}$$

where *W* was the total number of births in China during 2010-2013. We estimated *R_mild,p,t_*, *R_severe,p,t_* and *R_fatal,p,t_* from the provincial CDC internal laboratory records for each of the 19 scenarios in Fig 2B. See “Uncertainty in test-negative cases” below for details.

Let *C_s_*, *_p_* and *Q_s_*, *_p_* be the expected cost and QALY loss per episode of EV71-HFMD with severity *s* in province *p*, *n_s_*,*_p_* be the number of EV71-HFMD cases with severity *s* in province *p* and $h_{s,p}=n_{s,p}/\sum_{p=1}^{31} n_{s,p}$ be the probability that a given EV71-HFMD case with severity *s* in the birth cohort was from province *p*. Let *f_s,p,a_* be the age distribution (*a* = 0, 1, 2, 3, 4 years) of EV71-HFMD cases with severity *s* in province *p* (based on lab-confirmed EV71-HFMD cases in the national HFMD surveillance database from 2010-2013). The discounted expected cost and QALY loss due to EV71-HFMD of severity *s* per individual of a birth cohort were:

$$\begin{matrix} C_{s}=\sum_{p=1}^{31} h_{s,p}d_{s,p}(r_{C})C_{s,p}, \\ Q_{s}=\left\{\begin{matrix} \sum_{p=1}^{31} h_{s,p}d_{s,p}(r_{Q})Q_{s,p} & \text{if }s\text{ = }mild\text{ or }severe\text{,} \\ \sum_{p=1}^{31} \left[h_{s,p}(d_{s,p}(r_{Q})Q_{s,p}+Q_{death,p})\right] & \text{if }s\text{ = }fatal. \end{matrix}\right. \end{matrix}$$

where

- $d_{s,p}(r)=\sum_{a=0}^{4} f_{s,p,a}\left(\frac{1}{1+r}\right)^{a}$was the multiplier that corresponded to discounting costs or QALYs according to the age distribution *f_s,p,a_* at an annual discount rate of *r*.
- $Q_{death,p}=\sum_{a=0}^{4} f_{fatal,p,a}\sum_{b=a}^{M} \left(\frac{1}{1+r_{Q}}\right)^{b}$ was the expected QALY loss due to premature death according to the age distribution of fatal cases in province *p* with life expectancy *M* = 75 years in China [1].

The expected cost and QALY loss for EV71-HFMD per individual of a birth cohort were

$$\begin{matrix} C=P_{mild}C_{mild}+P_{severe}C_{severe}+P_{fatal}C_{fatal}, \\ Q=P_{mild}Q_{mild}+P_{severe}Q_{severe}+P_{fatal}Q_{fatal}. \end{matrix}$$

Uncertainty in test-negative cases

As described in the main text, the national HFMD surveillance database does not record the laboratory results of test-negative cases. To account for the resulting uncertainty, we acquired the internal laboratory serotyping records from all 31 provincial CDCs for 2010-2013. For Zhejiang, test-negative results were not recorded until 2012. As such, we assumed that in Zhejiang, the percentage of test results that were test-negative in 2010 and 2011 was the same as that in 2012. The 19 scenarios (A-S) in Fig 2B corresponded to assumptions regarding (i) the percentage of test-negative cases that were mild during 2010-2012; (ii) the percentage of test-negative mild cases that were EV71 during 2010-2013; and (iii) the percentage of test-negative severe/fatal cases that were EV71 during 2010-2013. On (i), we considered three possibilities:

1. The percentage of test-negative cases that were mild was the same as that for test-positive cases unless the resulting number of severe/fatal cases exceeded the number of severe/fatal cases recorded in the national database, in which case the excess were classified as mild cases.
2. Test-negative cases comprised as many severe/fatal cases as possible, i.e. all test-negative cases were severe/fatal unless the resulting number of severe/fatal cases exceeded the number of severe/fatal cases recorded in the national database, in which case the excess was classified as mild cases.
3. All test-negative cases were mild.

On (ii), we considered the following three possibilities:

1. None of the test-negative mild cases were EV71.
2. The percentage of test-negative mild cases that were EV71 was the same as that for test-positive mild cases.
3. All test-negative mild cases were EV71.

Similarly, on (iii), we considered the following three possibilities:

1. None of the test-negative severe/fatal cases were EV71.
2. The percentage of test-negative severe/fatal cases that were EV71 was the same as that for test-positive severe/fatal cases.
3. All test-negative severe/fatal cases were EV71.

S1-4 Table show the percentage of EV71 among all HFMD cases and test-negative cases in each province, respectively, in each test-negative scenario.

### Survey on household costs and quality of life detriment associated with EV71-HFMD

A telephone survey was conducted of parents or caregivers of children aged 6 months to 5 years with laboratory-confirmed EV71-HFMD occurring between 1 Jan 2012 and 17 Dec 2013 in the national HFMD enhanced surveillance database. Information about children’s diagnosis, treatment, household expenses, time off work and quality of life related to the HFMD episode was obtained from respondents who consented to be interviewed.

Health-related quality of life was measured using the EuroQol EQ-5D-3L instrument in simplified Chinese (China) ([www.euroqol.org](http://www.euroqol.org)). Since there was no EQ-5D version designed for use in children under 7 years of age, we adapted the adult proxy version by removing the questions on self-care and mobility, and conservatively assigning them a rating of “no problems”. Since patients with underlying health conditions were excluded from the study, we assumed that they would have been in perfect health if they had not been infected with EV71-HMFD. Ratings on the dimensions were converted into utility score *U* using the Chinese tariff [2], and QALY loss for that episode of EV71-HFMD was estimated as (1 − *U*) × (reported duration of illness).

Costs were collected on: (i) direct medical costs included payments for the first four outpatient and emergency attendances, the first two hospitalizations related to the EV71-HFMD episode, and payments for drugs and treatment outside of clinics and hospitals; (ii) direct non-medical costs included transport, food, accommodation and nursery care for patients and their caregivers; (iii) indirect costs included only the income loss of parents or caregivers, calculated by multiplying reported days off work by provincial urban and rural average income per capita in China. Productivity loss due to premature death was not included.

Initially, 52,838 records of patients with HFMD were identified. Of these, 29,810 had their parents or caregivers contacted in order to be interviewed. Quota sampling was employed in order to obtain equal representation from seven regions in China (see S6 Table). 19,873 unique telephone numbers were dialed successfully and 3,500 led to successful interviews. Of these, 1,787 were parents or caregivers of EV71-HFMD patients and hence their responses were used in this analysis. S7 Table shows the demographic characteristics of the sample obtained, while S8-9 Table show the costs and quality of life weights associated with EV71-HFMD. We used the non-parametric Kruskal–Wallis test to check whether the age and sex distributions of EV71-HFMD patients were significantly different between the respondents and non-respondents within each severity-region stratum (S7 Fig). After correcting for multiple testing of the same hypothesis in 7 different regions using false discovery rate control, we only detected significant difference in age in the South region (*p* < 0.05). Similarly, we found no evidence that the serotype distribution was different between respondents and non-respondents among the HFMD patients that we contacted in the survey.

We examined the robustness of our conclusions to changes in the methodology for analyzing the survey data:

1. *Cost and QALY loss for fatal cases.* Because the number of fatal cases was too low to accurately pinpoint inter-region differences, we assumed that the expected costs and QALY losses not due to life years lost were the same for all EV71-HFMD fatal cases (i.e. no geographical dependence). Our results were robust against this assumption because (i) fatal cases accounted for <1% of the expected cost of EV71-HFMD (Fig 3C) and (ii) almost all of the QALY loss incurred by fatal cases was due to life years loss from pediatric premature death (Fig 3C) which depended only on age.
2. When providing the estimated costs associated with HFMD in our survey, the parent or caregiver of each subject was first asked to give a numerical point estimate. If he/she could not provide a point estimate, he/she was asked to choose one of the pre-specified ranges. For those who provided a given cost range [*L*, *U*], we assumed that their expected cost was the lower limit of this range, i.e. *L*. Our results were robust against this assumption because *EVC_max_* changed by <8% if we assumed that their expected cost was the midpoint ((*L*+*U*)/2) or upper limit (*U*) of the range instead.

In the survey, we estimated health utility using a questionnaire based on EQ-5D-3L in which the self-care domain was omitted because children under the age of 5 are generally not capable of self-care. For children under the age of 18 months, we also omitted the mobility domain. We assigned a rating of 1 (meaning “no impairment”) to the self-care and mobility domains where missing. Our CEA results were robust against this assumption; *EVC_max_* increased by <8% if we adopted the opposite extreme and assigned a rating of 3 (meaning “severe impairment”, the extreme opposite of “no impairment” in EQ-5D-3L) to these domains. We used the EQ-5D-3L value set for China [2] (model 8 in Table 3 therein) to translate the EQ-5D-3L data into quality-of-life (QoL) during HFMD illness for each subject. For each level of severity (mild outpatients, mild inpatients, severe cases and fatal cases), we estimated the QALY loss for each EV71-HFMD subject in the survey by multiplying his/her QoL loss by his/her self-reported duration of HFMD illness (plus life years loss due to premature death for fatal cases). When calculating the indirect costs, we used the average income in each province in 2013 [3] to translate the number of workdays lost (among parents or guardians of the pediatric patients) into monetary figures (S10 Table).

Cost was a priori assumed to be associated with disease severity and geographical region. We performed univirate hypothesis testing to see if costs or QALY loss were statistically associated with age, sex or urban residence. Since the distributions of costs were highly right skewed in all severity-region strata (S5 Fig), we analyzed the cost and QALY loss data with the non-parametric Kruskal–Wallis test. The resulting p-values in the base case are shown in S11 Table. After correcting for multiple testing of the same hypothesis in the seven regions (using false discovery rate control method), we found that none of the demographic factors was significantly associated with costs or QALY loss in more than one region. Consequently, we stratified our calculation of expected costs and QALY loss by disease severity and geographical region only.

Let *C_s_*, *_d_* and *Q_s_*, *_d_* be the expected cost and QALY loss per episode of EV71-HFMD with severity *s* in region *d*. Let *M_C_s_*, *_d_* and *M_Q_s_*, *_d_* be their sample mean, *V_C_s_*, *_d_* and *V_Q_s_*, *_d_* their sample variance and *CV_C_s_*,*d* *_Q_s_*, *_d_* their sample covariance in our survey data. Let *Y_s_*, *_d_* be the corresponding sample covariance matrix. By the Central Limit Theorem, our estimates of *C_s_*, *_d_* and *Q_s_*, *_d_* followed (approximately) a bivariate normal distribution with mean (*M_C_s_*, *_d_*, *M_Q_s_*, *_d_*) and covariance matrix *Y_s_*, *_d_* / *n_s_*, *_d_* where *n_s_*, *_d_* was the number of subjects with severity *s* in region *d* (S12 Table). We have performed a sensitivity analysis using bootstrapping to estimate the expected costs and QALY loss and the resulting mean and 95% CIs of *EVC_max_* were almost the same as that obtained using the Central Limit Theorem (results not shown).

Mild outpatients and mild inpatients had significantly different costs and QALY loss (S8-S9 Table). As such, to estimate the overall expected costs and QALY loss for mild cases, we accounted for the percentage of mild HFMD cases that were inpatients in each province. S10 Table shows that the percentage of mild HFMD cases that were inpatients varied substantially among provinces. The expected costs and QALY loss of mild cases were simply the corresponding weighted average among mild outpatient and inpatients. S13 Table shows the resulting expected QALY loss, expected cost for mild, severe and fatal EV71-HFMD in each province.

### Estimation of the unregistered proportion

To assess the proportion of EV71-HFMD cases not captured by national HFMD surveillance, we compared the incidence rate of EV71-HFMD reported in the three EV71 vaccine phase III trials with the spatiotemporally matched incidence rate of EV71-HFMD in the national surveillance database [4-6]. We estimated the EV71-HFMD incidence rate in each phase III trial using the number of subjects and the number of EV71-associated diseases in the placebo group of each trial (S14 Table).

Let *K_X_*,*_T_* and *U_X_*,*_T_* be the number of HFMD and EV71-HFMD cases registered by national surveillance for county *X* during the study period of vaccine trial *T*. We assumed that *U_X_*,*_T_* = *K_X_*,*_T_R_X_*,*_T_* where *R_X_*,*_T_* was the proportion of HFMD cases that were attributed to EV71 (spatiotemporally matched). We assumed that *R_X_*,*_T_* was the same as the proportion of test-positive cases that were EV71 because test-negative results were only available at the provincial level but not at the county level (see data source 2 in the main text). S14-15 Table show our estimates of *R_X_*,*_T_*, the corresponding incidence rate of EV71-HFMD and the ratio of EV71-HFMD incidence rate in national surveillance compared to that in the phase III trials.

### Indirect protection conferred by vaccination

The average age of EV71-HFMD cases in the national surveillance was around 3 years. Assuming that EV71-HFMD is endemic (with the yearly boom-bust epidemic cycles attributed to seasonality), a rough estimate of the basic reproductive number *R*_0_ for EV71-HFMD is (life expectancy)/(average age of cases) = 25 assuming a life expectancy of 75 years. In a separate study, we performed a comprehensive modeling study of HFMD epidemic dynamics in China to show that the national average *R*_0_ is around 27 [7]. Indirect protection conferred by mass vaccination is likely to have a minor impact at such high values of *R*_0_.

Here, assuming that *R*_0_ is above 25, we used the simple SIR model to show that the static model used in the main text captured more than 96% of the reduction in risk of infection conferred by vaccination when herd immunity was explicitly accounted for. As a simple illustration, we assumed that the disease was at endemic equilibrium and the population size was constant (i.e. the birth rate was equal to the death rate). Let *R*_0_ be the basic reproductive number, *B* be the birth rate per capita and *D* be the average disease duration. The SIR model with routine vaccination of birth cohort at coverage *X* was described by the differential equations

$$\frac{dS}{dt}=B\left( 1-X \right)-\frac{R_{0}}{D}SI-BS$$

$$\frac{dI}{dt}=\frac{R_{0}}{D}SI-\frac{I}{D}-BI$$

where *S* and *I* were the proportion of population who were susceptible and infectious, respectively. If the vaccine coverage *X* was above the critical threshold $1-\frac{1+BD}{R_{0}}$, the reproductive number was less than 1 and the disease was eradicated. At endemic equilibrium (i.e. $\frac{dS}{dt}=0, \frac{dI}{dt}=0$ with $X<1-\frac{1+BD}{R_{0}}$), the disease prevalence was $\frac{BD(R_{0}\left( 1-X \right)-1-BD)}{R_{0}(1+BD)}$. Therefore, the relative reduction in the risk of infection was (i) *X* in the static model and (ii) $X\left( \frac{R_{0}}{R_{0}-1-BD} \right)$ if $X<1-\frac{1+BD}{R_{0}}$ and 1 otherwise in the SIR model. The birth rate per capita *B* is around 0.012 per year for China while the average disease duration *D* is less than 2 weeks for HFMD. As such, for all realistic values of *B* and *D*, 1 + *BD* ≈ 1. Hence, the proportion of risk reduction in the SIR model that is captured by the static model is essentially $1-\frac{1}{R_{0}}$ if $X<1-\frac{1}{R_{0}}$ and $X$ otherwise. This shows that the static model captured more than 96% of the risk reduction in the SIR model if *R*_0_ > 25. This conclusion remained valid when seasonality and age-structure were present (results not shown).

### Under what conditions would we not vaccinate because a more cost-effective alternative intervention exists?

Suppose the average burden of EV-71 disease in China is *C* in costs and *Q* in QALY losses over the lifetime of an individual. A vaccine exists which costs *c*_1_ per fully vaccinated individual and which reduces that person’s risk of disease by *ε*_1_ over his or her lifetime. Hence the effective vaccine cost is *c*_1_/*ε*_1_. However, an alternative preventive measure (sanitation, hand hygiene, social distancing) exists which costs *c*_2_ per individual to which it is applied (in terms of its present value at birth) and reduces that person’s risk of disease by *ε*_2_ over his or her lifetime. Hence it has an effective cost of *c*_2_/*ε*_2_.

If the alternative measure is more cost-effective than vaccination (i.e. *c*_2_/*ε*_2_ < *c*_1_/*ε*_1_), then under what conditions would we choose not to vaccinate?

In the absence of the alternative, the vaccine is cost-effective if its ICER is less than our willingness-to-pay threshold (*T*), i.e.

$$\frac{c_{1}-C\varepsilon_{1}}{Q\varepsilon_{1}}<T$$

or

$$\frac{c_{1}}{\varepsilon_{1}}<TQ+C$$

Because the alternative is more cost-effective, we will choose to fund the alternative before considering vaccination. We will only add on vaccination if the ICER of adding vaccination to the alternative is less than *T*, i.e.

$$\frac{c_{1}-C(1-\varepsilon_{2})\varepsilon_{1}}{Q(1-\varepsilon_{2})\varepsilon_{1}}<T$$

This is because the alternative has reduced the burden of disease to *C*(1 − *ε*_2_) in costs and *Q*(1 − *ε*_2_) in QALYs lost. This equation reduces to:

$$\frac{c_{1}}{\varepsilon_{1}}<(TQ+C)(1-\varepsilon_{2})$$

Note that if *ε*_2_ = 0 (the alternative measure is completely useless), then we need *c*_1_/*ε*_1_> *TQ* + *C* as before without the alternative. From our data, we know that *C* = $10.6 (95% CI 9.8 – 11.5), *Q* = 10.8 (10.7-10.9) per 10,000 and *T* = $6,700. S6 Fig shows the threshold for effective vaccine cost as the effectiveness of the alternative increases.

In order to determine the potential effectiveness of alternative interventions to prevent the spread of EV71, we conducted a rapid review by searching OVID Medline(R) using the search terms below:

1. (Enterovirus/ or Enterovirus Infections/ or "Hand, Foot and Mouth Disease"/ or Enterovirus A, Human/) … 9815 results
2. (Hand Hygiene/ or Infection Control/ or Hand Disinfection/ or Communicable Disease Control/ or Sanitation/) … 49339 results\
3. 1 and 2 … 49 results

We then scanned the titles and abstracts of the 49 articles and found 4 that were potentially relevant [8-11]. We obtained the full text of the four articles and found that only two of them [10, 11] had potentially relevant information.

Ruan et al. conducted a case-control study of hand hygiene in Yuhang district, southeast China [11]. They reported that very good handwashing habits in pre-school children and their parents reduced the risk of acquiring EV-71 by more than 95% compared to those children and parents with poor handwashing habits. However, this was an observational rather than interventional study so we were not able to obtain any information about a population-level intervention that may achieve this level of handwashing adherence among a large enough portion of the population over the long-term.

Ma et al. conducted a time-series analysis of HMFD cases in Hong Kong from January 2001 and June 2001 [10]. They found a 57.2% reduction in HFMD incidence during the SARS outbreak in 2003, and a 26.7% reduction in HFMD incidence during the H1N1 influenza pandemic in 2009. The SARS outbreak in particular was associated in Hong Kong with extensive territory-wide infection control measures including school closure, public awareness campaigns on hand and respiratory hygiene and disinfection. Hence this might represent the largest reduction possible from a population-wide intervention. If 57.2% reduction in HFMD incidence was achieved, *EVC_max_* would fall to $7.34.

### Comparative CEA of the three vaccine candidates

If vaccines A and B have efficacies $\varepsilon_{A}>\varepsilon_{B}$ and their costs are $V_{A}>V_{B}$, choosing vaccine A over vaccine B is cost-effective if and only if the corresponding incremental cost-effective ratio (ICER) is lower than the willingness-to-pay threshold *T*. As in the main text, we use *C* and *Q* to denote the expected cost and health utility loss due to EV71-HFMD per birth, respectively. The ICER for choosing vaccine A over vaccine B is simply

$\frac{\left(V_{A}-\varepsilon_{A}C\right)-\left(V_{B}-\varepsilon_{B}C\right)}{\varepsilon_{A}Q-\varepsilon_{B}Q}=\frac{\left(V_{A}-V_{B}\right)/\left(\varepsilon_{A}-\varepsilon_{B}\right)-C}{Q}$.

Hence, choosing vaccine A over vaccine B is cost-effective if and only if $\frac{V_{A}-V_{B}}{\varepsilon_{A}-\varepsilon_{B}}<EVC_{\max}$ where $EVC_{\max}=C+QT$ is the cost-effectiveness ceiling for effective vaccine cost (as in the main text).

Let *V_Vigoo_*, *V_Sinovac_* and *V_CAMS_*, be the costs for the Vigoo, Sinovac and CAMS vaccine and *ε_Vigoo_*, *ε_Sinovac_* and *ε_CAMS_* their efficacies. For any given combination of *V_Vigoo_*, *V_Sinovac_* and *V_CAMS_*, we determined the optimal vaccine candidate as follows:

1. 100,000 scenarios of , *ε_Vigoo_*, *ε_Sinovac_* and *ε_CAMS_* were randomly generated where (i) followed a normal distribution with mean $17.9, 2.5^th^ percentile $16.9 and 97.5^th^ percentile $18.8 (the base case in the main text) and (ii) 1−*ε_Vigoo_*, 1−*ε_Sinovac_* and 1−*ε_CAMS_* followed lognormal distributions in which the median, 2.5th and 97.5th percentiles were the same as the point estimate and 95% CI of the vaccine efficacy reported in their clinical trials.
2. In each scenario, the most efficacious vaccine with an ICER below the willingness-to-pay threshold when compared to the next most efficacious nondominated vaccine was the most cost-effective.
3. The optimal vaccine (for the given combination of *V_Vigoo_*, *V_Sinovac_* and *V_CAMS_*) was the one that had the highest probability of being the most cost-effective among the 100,000 scenarios.


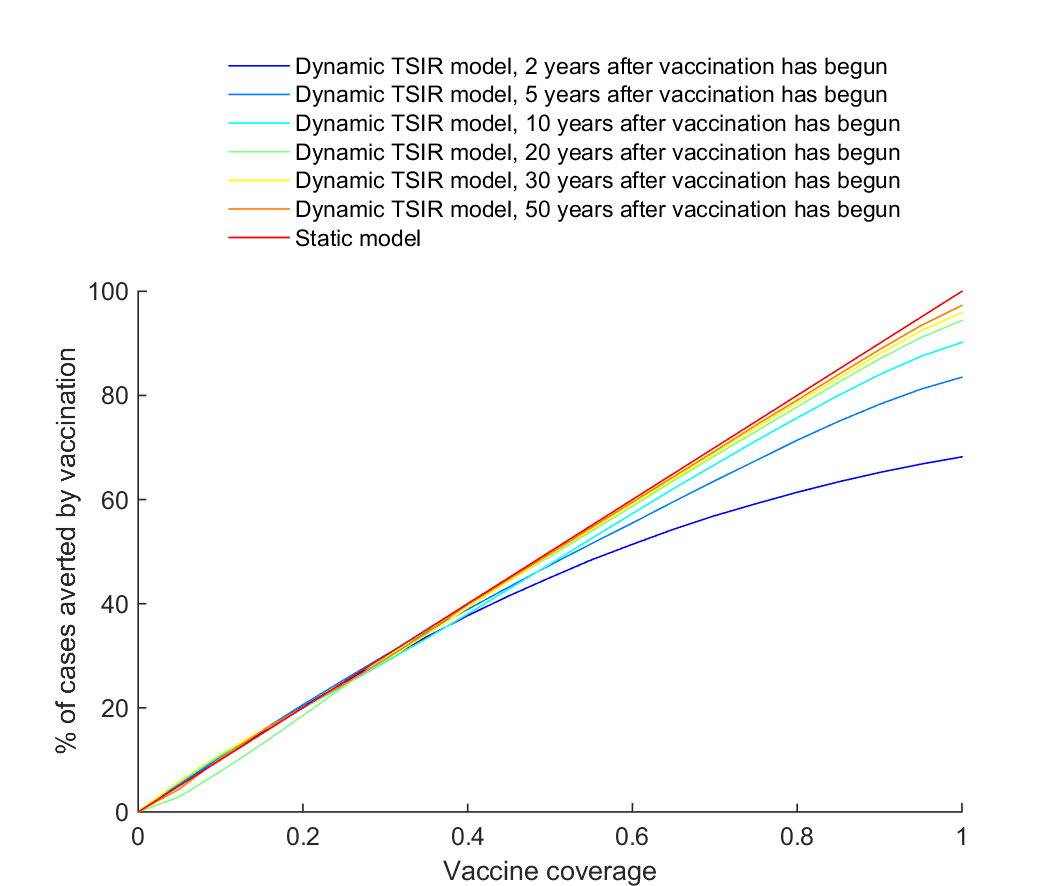


**S1 Fig.** Comparison of the incidence reduction predicted in the static model and that predicted in the TSIR dynamic model in Takahashi et al [7]. The TSIR model predictions presented here were generated using the model in Figure 3D of Takahashi et al [7] for 2, 5, 10, 20, 30 and 50 years after vaccination has begun. Predictions in the static and dynamic models are essentially the same because the basic reproductive number is high (with a national average of 27). Incidence in the TSIR dynamic model was slightly higher than that in the static model when vaccine coverage was near 1 because the epidemic had not yet completely reached equilibrium.


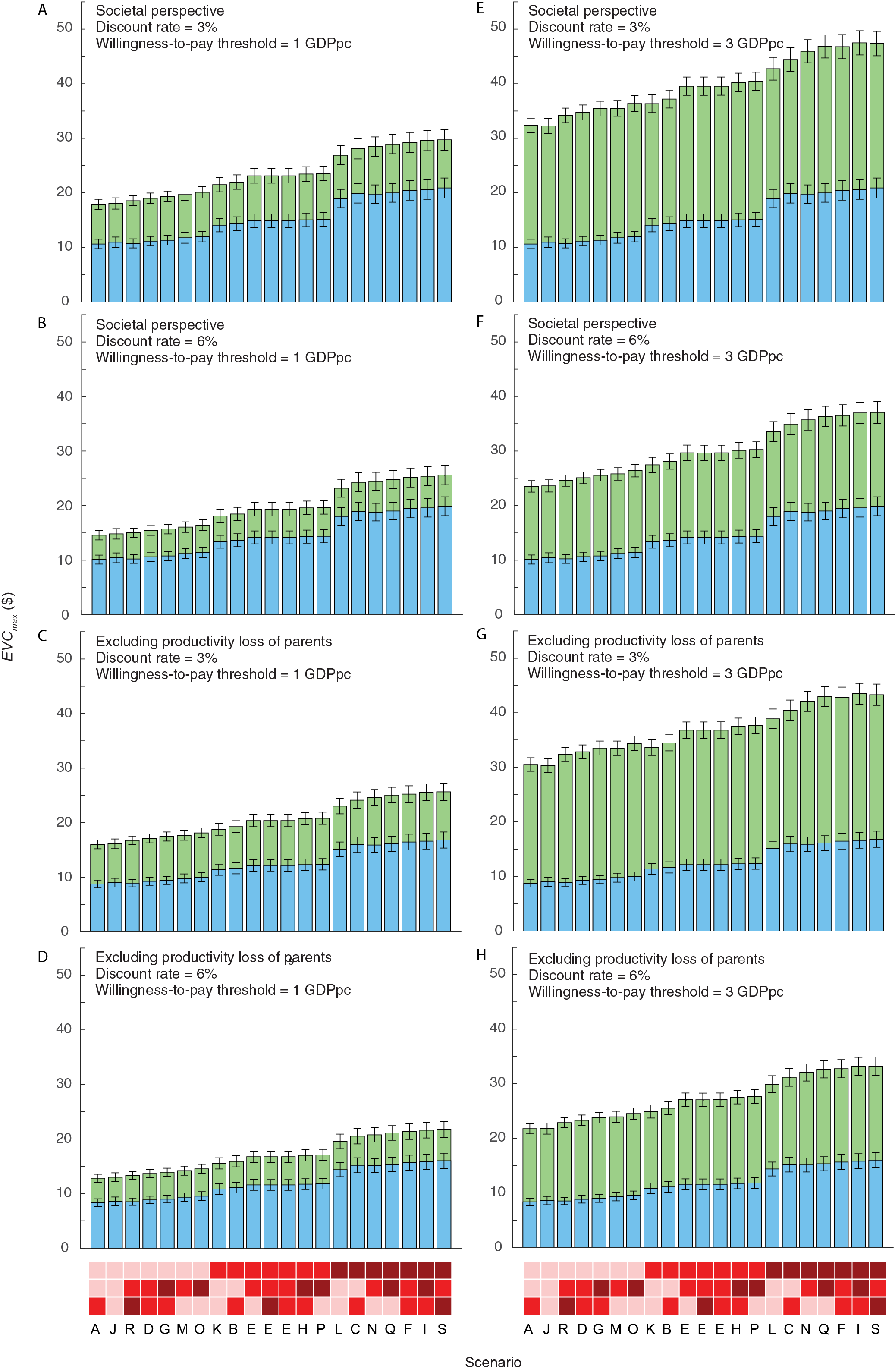


**S2 Fig. Cost-effectiveness of routine pediatric EV71 vaccination in China. A.** Base case, i.e. same as Figure 3A. **B-H** All other scenarios considered in the uncertainty analysis by including or excluding productivity loss, discounting cost and health utility at 3% or 6%, and setting the willingness-to-pay threshold at one or three times GDPpc.


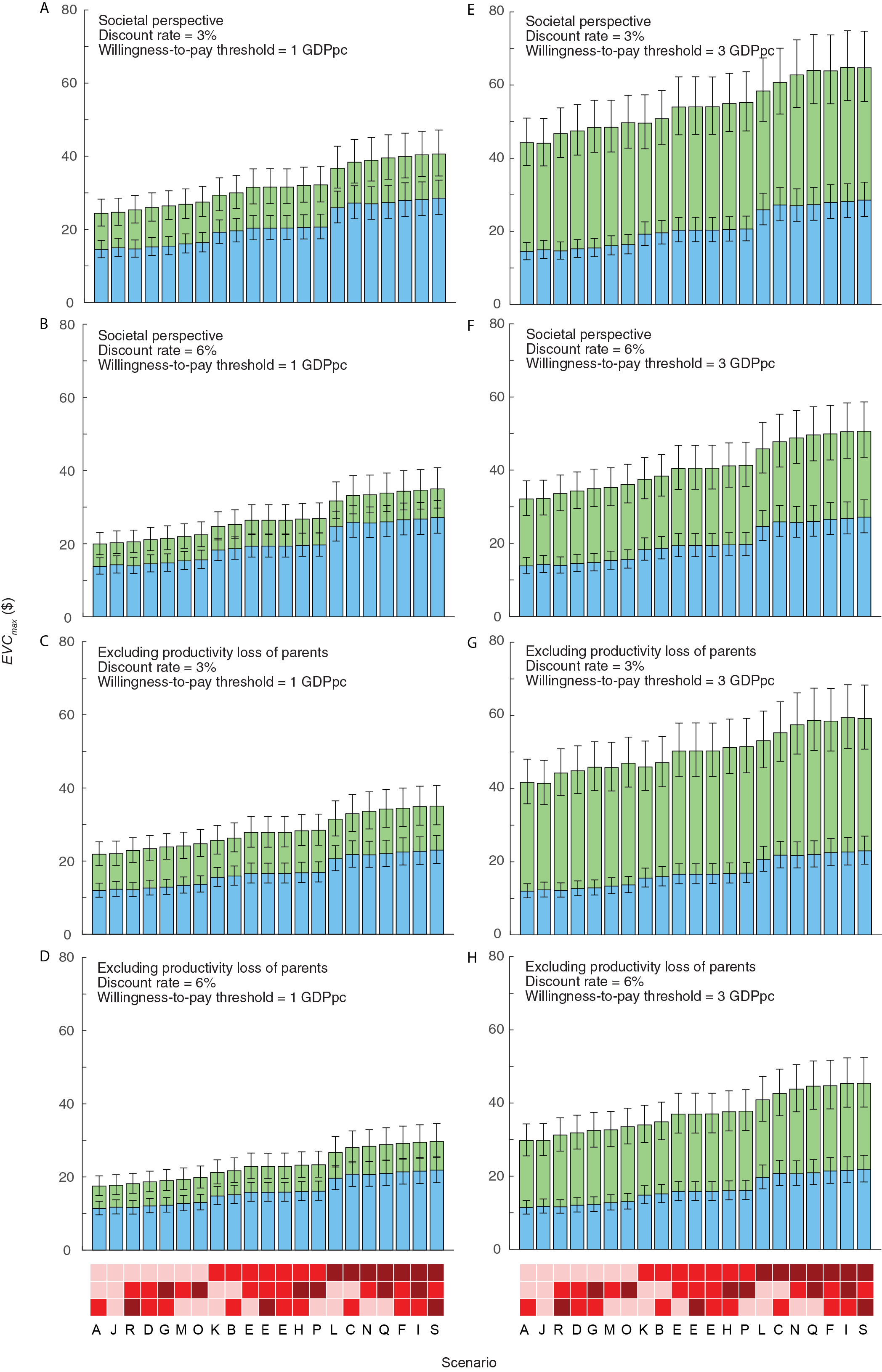


**S3 Fig. Cost-effectiveness of routine pediatric EV71 vaccination in China accounting for the effect of underreporting.** Same as S2 Fig but the proportion of EV71-HFMD cases registered by national surveillance was assumed to be the same as that estimated from the three EV71 vaccine trials, i.e. 74% (95% CI 64%-84%). See “Estimation of the unregistered proportion” in S1 Text and S15 Table for details.

**
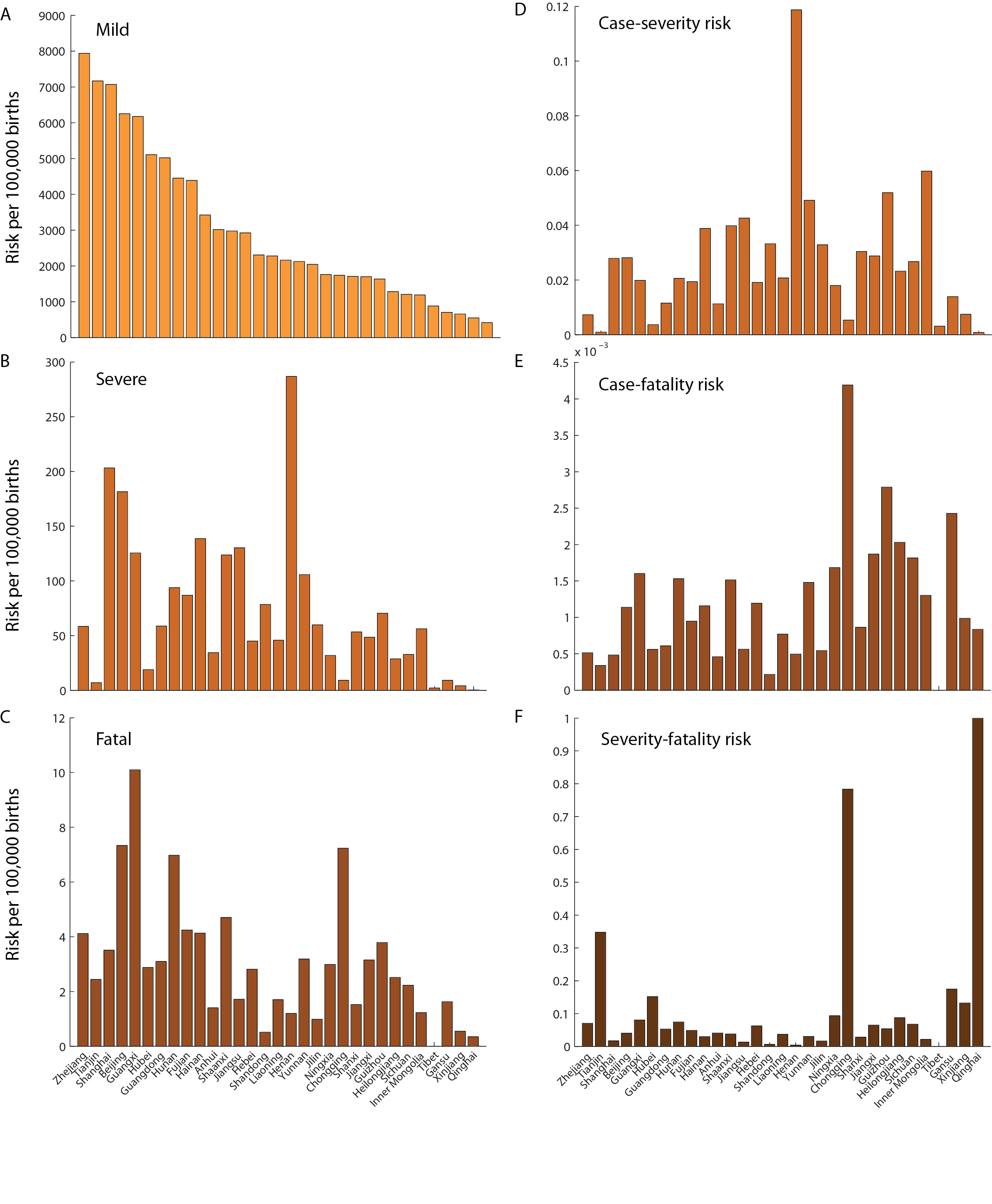
**

**S4 Fig. Geographical variation in the risk and severity of EV71-HFMD among children under the age of 5 in China.** **A-C** Risk of mild, severe and fatal cases of EV71-HFMD. **D-F** Case-severity, case-fatality and severity-fatality risk.


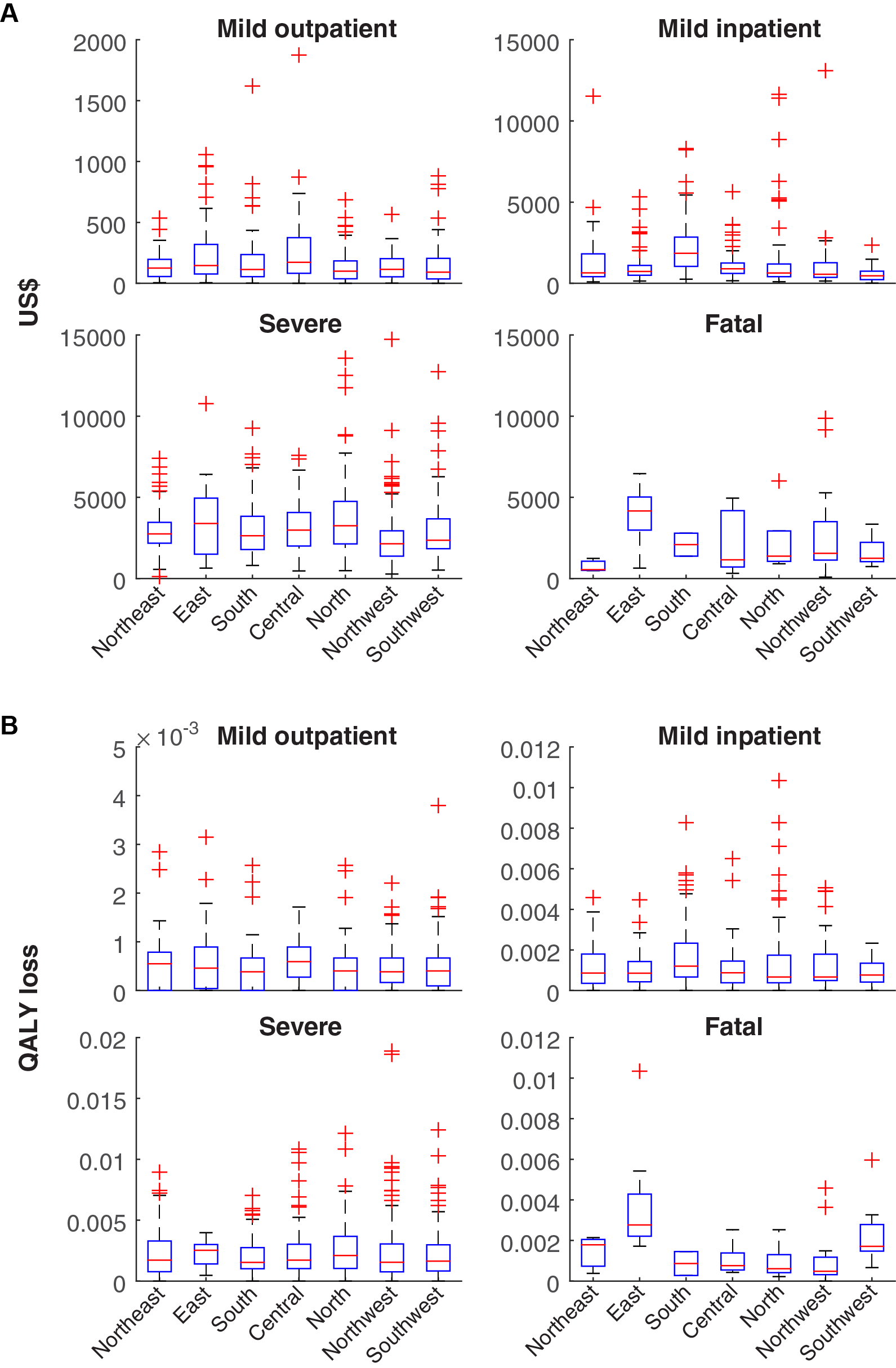


**S5 Fig. The distributions of costs and QALY loss per episode of EV71-HFMD in the survey stratified by severity and geographical region. A** Costs. **B** QALY loss during illness. In each box, the central mark is the median; the edges of the box are the 25^th^ and 75^th^ percentiles; the whiskers extend to the extreme data points not considered as outliners; and outliners are plotted individually with “+” marks. Data points are considered as outliners if they are larger than *q*_75_ + 1.5 × (*q*_75_ – *q*_25_) or smaller than *q*_25_ -1.5 × (*q*_75_ – *q*_25_) where *q*_25_ and *q*_75_ are the 25^th^ and 75^th^ percentiles.


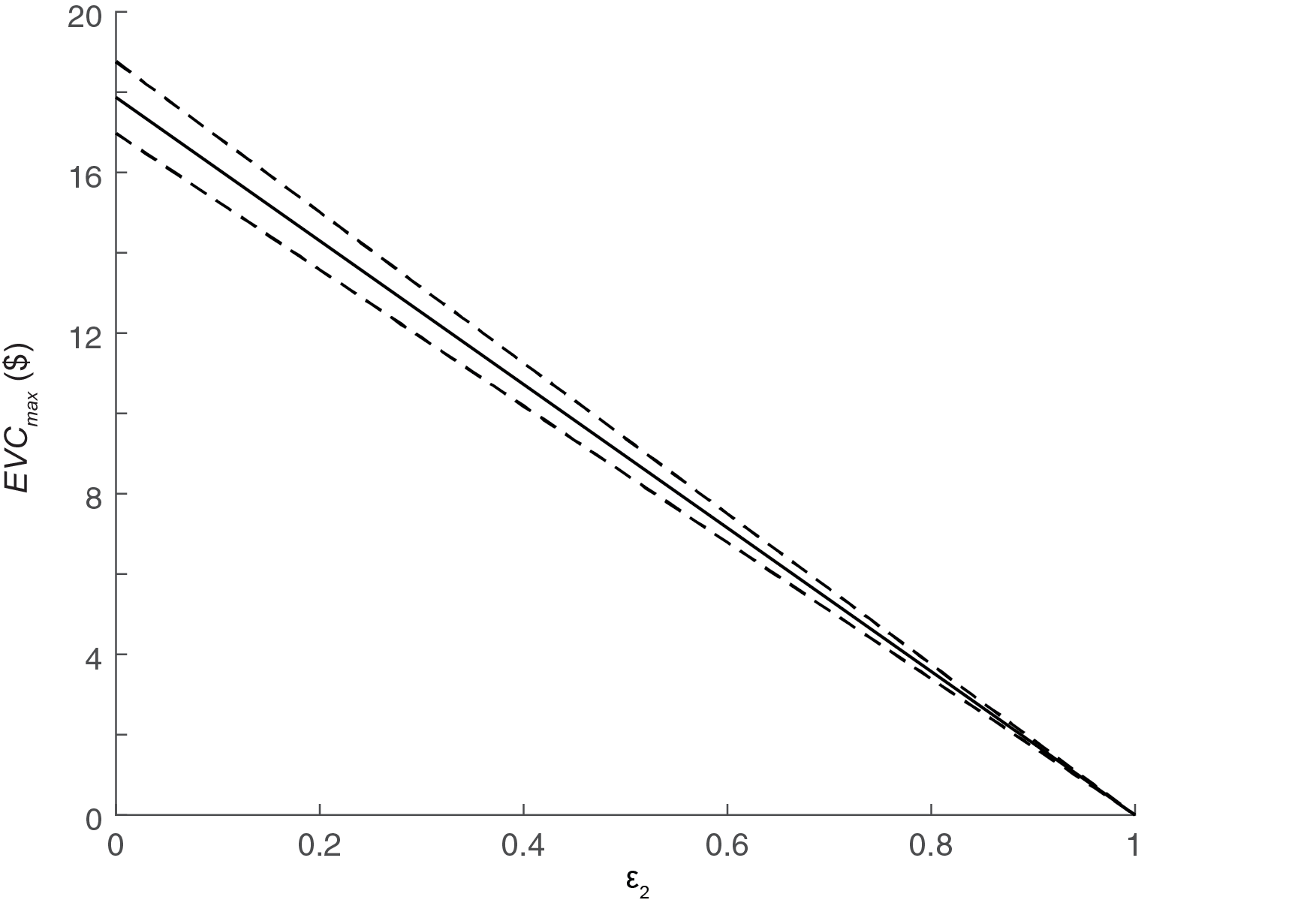


**S6 Fig. *EVC_max_* as a function of the effectiveness of a more cost-effective alternative (e.g. hand hygiene or social distancing).** Solid and dashed lines indicate the mean and 95% CI.


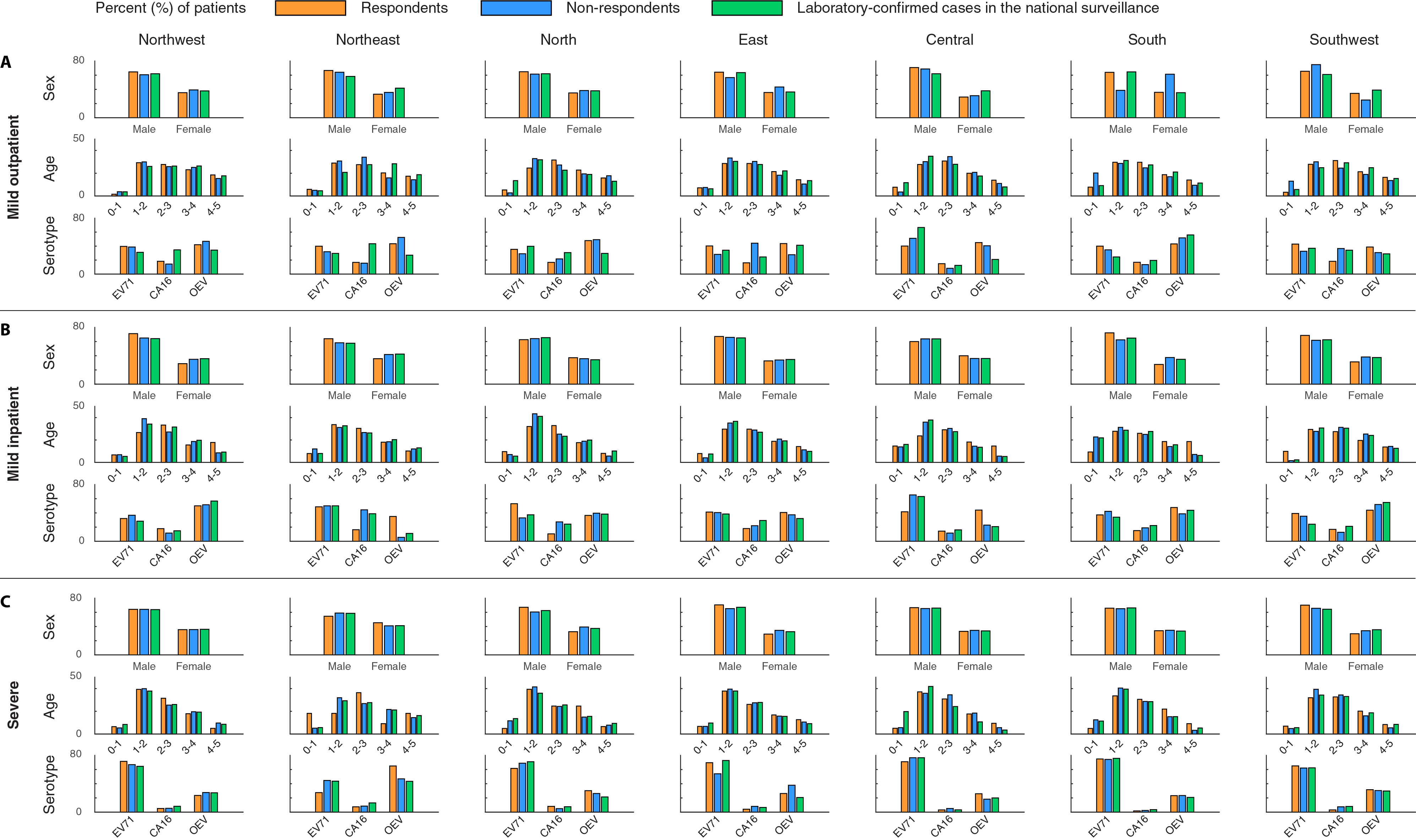


**S7 Fig**. **The sex, age and serotype distribution of survey respondents, survey non-respondents, and the laboratory-confirmed cases in the national surveillance in each of the 7 geographical regions**. **A** mild outpatient cases. **B** mild inpatient cases and **C** severe cases. Sex and age distribution were obtained from laboratory-confirmed EV71 HFMD cases, and serotype distribution was from laboratory-confirmed HFMD cases.

| **Province** | **Percentage of EV71-HFMD among severe/fatal HFMD cases** | | | | | | | | | | | | | | | | | | |
| --- | --- | --- | --- | --- | --- | --- | --- | --- | --- | --- | --- | --- | --- | --- | --- | --- | --- | --- | --- |
|  | **A** | **B** | **C** | **D** | **E** | **F** | **G** | **H** | **I** | **J** | **K** | **L** | **M** | **N** | **O** | **P** | **Q** | **R** | **S** |
| Anhui | 58% | 58% | 58% | 87% | 87% | 87% | 92% | 92% | 92% | 55% | 55% | 55% | 87% | 87% | 92% | 92% | 92% | 88% | 88% |
| Beijing | 32% | 32% | 32% | 58% | 58% | 58% | 75% | 75% | 75% | 17% | 17% | 17% | 60% | 60% | 86% | 86% | 86% | 56% | 56% |
| Chongqing | 55% | 55% | 55% | 73% | 73% | 73% | 80% | 80% | 80% | 55% | 55% | 55% | 73% | 73% | 80% | 80% | 80% | 73% | 73% |
| Fujian | 52% | 52% | 52% | 76% | 76% | 76% | 83% | 83% | 83% | 50% | 50% | 50% | 77% | 77% | 84% | 84% | 84% | 74% | 74% |
| Gansu | 58% | 58% | 58% | 79% | 79% | 79% | 85% | 85% | 85% | 53% | 53% | 53% | 79% | 79% | 86% | 86% | 86% | 79% | 79% |
| Guangdong | 63% | 63% | 63% | 78% | 78% | 78% | 82% | 82% | 82% | 63% | 63% | 63% | 78% | 78% | 82% | 82% | 82% | 78% | 78% |
| Guangxi | 54% | 54% | 54% | 72% | 72% | 72% | 80% | 80% | 80% | 49% | 49% | 49% | 73% | 73% | 82% | 82% | 82% | 70% | 70% |
| Guizhou | 34% | 34% | 34% | 58% | 58% | 58% | 75% | 75% | 75% | 34% | 34% | 34% | 58% | 58% | 75% | 75% | 75% | 55% | 55% |
| Hainan | 29% | 29% | 29% | 80% | 80% | 80% | 93% | 93% | 93% | 17% | 17% | 17% | 80% | 80% | 96% | 96% | 96% | 78% | 78% |
| Hebei | 62% | 62% | 62% | 81% | 81% | 81% | 86% | 86% | 86% | 62% | 62% | 62% | 81% | 81% | 86% | 86% | 86% | 81% | 81% |
| Heilongjiang | 63% | 63% | 63% | 87% | 87% | 87% | 91% | 91% | 91% | 63% | 63% | 63% | 87% | 87% | 91% | 91% | 91% | 85% | 85% |
| Henan | 60% | 60% | 60% | 82% | 82% | 82% | 87% | 87% | 87% | 42% | 42% | 42% | 83% | 83% | 91% | 91% | 91% | 82% | 82% |
| Hubei | 66% | 66% | 66% | 84% | 84% | 84% | 87% | 87% | 87% | 66% | 66% | 66% | 84% | 84% | 87% | 87% | 87% | 84% | 84% |
| Hunan | 46% | 46% | 46% | 71% | 71% | 71% | 81% | 81% | 81% | 39% | 39% | 39% | 72% | 72% | 84% | 84% | 84% | 70% | 70% |
| Inner Mongolia | 42% | 42% | 42% | 79% | 79% | 79% | 89% | 89% | 89% | 42% | 42% | 42% | 79% | 79% | 89% | 89% | 89% | 79% | 79% |
| Jiangsu | 43% | 43% | 43% | 72% | 72% | 72% | 84% | 84% | 84% | 38% | 38% | 38% | 73% | 73% | 86% | 86% | 86% | 69% | 69% |
| Jiangxi | 58% | 58% | 58% | 81% | 81% | 81% | 87% | 87% | 87% | 53% | 53% | 53% | 81% | 81% | 88% | 88% | 88% | 81% | 81% |
| Jilin | 54% | 54% | 54% | 76% | 76% | 76% | 82% | 82% | 82% | 50% | 50% | 50% | 76% | 76% | 83% | 83% | 83% | 75% | 75% |
| Liaoning | 48% | 48% | 48% | 65% | 65% | 65% | 71% | 71% | 71% | 39% | 39% | 39% | 64% | 64% | 77% | 77% | 77% | 62% | 62% |
| Ningxia | 53% | 53% | 53% | 84% | 84% | 84% | 91% | 91% | 91% | 39% | 39% | 39% | 83% | 83% | 93% | 93% | 93% | 85% | 85% |
| Qinghai | 100% | 100% | 100% | 100% | 100% | 100% | 100% | 100% | 100% | 100% | 100% | 100% | 100% | 100% | 100% | 100% | 100% | 100% | 100% |
| Shaanxi | 45% | 45% | 45% | 69% | 69% | 69% | 79% | 79% | 79% | 39% | 39% | 39% | 70% | 70% | 82% | 82% | 82% | 67% | 67% |
| Shandong | 45% | 45% | 45% | 70% | 70% | 70% | 81% | 81% | 81% | 24% | 24% | 24% | 71% | 71% | 90% | 90% | 90% | 69% | 69% |
| Shanghai | 83% | 83% | 83% | 94% | 94% | 94% | 95% | 95% | 95% | 83% | 83% | 83% | 94% | 94% | 95% | 95% | 95% | 94% | 94% |
| Shanxi | 63% | 63% | 63% | 86% | 86% | 86% | 89% | 89% | 89% | 24% | 24% | 24% | 88% | 88% | 96% | 96% | 96% | 86% | 86% |
| Sichuan | 48% | 48% | 48% | 66% | 66% | 66% | 75% | 75% | 75% | 48% | 48% | 48% | 66% | 66% | 75% | 75% | 75% | 65% | 65% |
| Tianjin | 16% | 16% | 16% | 79% | 79% | 79% | 86% | 86% | 86% | 12% | 12% | 12% | 91% | 91% | 97% | 97% | 97% | 82% | 82% |
| Tibet | 14% | 14% | 14% | 100% | 100% | 100% | 100% | 100% | 100% | 14% | 14% | 14% | 100% | 100% | 100% | 100% | 100% | 100% | 100% |
| Xinjiang | 87% | 87% | 87% | 95% | 95% | 95% | 95% | 95% | 95% | 84% | 84% | 84% | 93% | 93% | 95% | 95% | 95% | 94% | 94% |
| Yunnan | 52% | 52% | 52% | 84% | 84% | 84% | 90% | 90% | 90% | 39% | 39% | 39% | 84% | 84% | 92% | 92% | 92% | 83% | 83% |
| Zhejiang | 80% | 80% | 80% | 83% | 83% | 83% | 85% | 85% | 85% | 80% | 80% | 80% | 83% | 83% | 85% | 85% | 85% | 84% | 84% |

**S1 Table. The percentage of EV71-HFMD cases among all severe/fatal HFMD cases in each province in each of the 19 test-negative scenarios.**

| **Province** | **Percentage of EV71-HFMD among mild HFMD cases** | | | | | | | | | | | | | | | | | | |
| --- | --- | --- | --- | --- | --- | --- | --- | --- | --- | --- | --- | --- | --- | --- | --- | --- | --- | --- | --- |
|  | **A** | **B** | **C** | **D** | **E** | **F** | **G** | **H** | **I** | **J** | **K** | **L** | **M** | **N** | **O** | **P** | **Q** | **R** | **S** |
| Anhui | 27% | 51% | 74% | 27% | 51% | 74% | 27% | 51% | 74% | 27% | 51% | 74% | 27% | 74% | 27% | 51% | 74% | 26% | 75% |
| Beijing | 19% | 36% | 63% | 19% | 36% | 63% | 19% | 36% | 63% | 21% | 35% | 61% | 21% | 61% | 21% | 35% | 61% | 19% | 65% |
| Chongqing | 20% | 30% | 54% | 20% | 30% | 54% | 20% | 30% | 54% | 20% | 30% | 54% | 20% | 54% | 20% | 30% | 54% | 20% | 54% |
| Fujian | 28% | 44% | 63% | 28% | 44% | 63% | 28% | 44% | 63% | 28% | 44% | 63% | 28% | 63% | 28% | 44% | 63% | 27% | 64% |
| Gansu | 22% | 36% | 61% | 22% | 36% | 61% | 22% | 36% | 61% | 22% | 36% | 61% | 22% | 61% | 22% | 36% | 61% | 22% | 62% |
| Guangdong | 21% | 28% | 45% | 21% | 28% | 45% | 21% | 28% | 45% | 21% | 28% | 45% | 21% | 45% | 21% | 28% | 45% | 20% | 46% |
| Guangxi | 19% | 25% | 45% | 19% | 25% | 45% | 19% | 25% | 45% | 20% | 24% | 43% | 20% | 43% | 20% | 24% | 43% | 18% | 48% |
| Guizhou | 16% | 32% | 66% | 16% | 32% | 66% | 16% | 32% | 66% | 16% | 32% | 66% | 16% | 66% | 16% | 32% | 66% | 15% | 68% |
| Hainan | 10% | 23% | 67% | 10% | 23% | 67% | 10% | 23% | 67% | 11% | 22% | 62% | 11% | 62% | 11% | 22% | 62% | 9% | 71% |
| Hebei | 31% | 50% | 69% | 31% | 50% | 69% | 31% | 50% | 69% | 31% | 50% | 69% | 31% | 69% | 31% | 50% | 69% | 31% | 70% |
| Heilongjiang | 25% | 49% | 75% | 25% | 49% | 75% | 25% | 49% | 75% | 25% | 49% | 75% | 25% | 75% | 25% | 49% | 75% | 25% | 75% |
| Henan | 34% | 48% | 63% | 34% | 48% | 63% | 34% | 48% | 63% | 43% | 46% | 53% | 43% | 53% | 43% | 46% | 53% | 30% | 67% |
| Hubei | 50% | 72% | 81% | 50% | 72% | 81% | 50% | 72% | 81% | 50% | 72% | 81% | 50% | 81% | 50% | 72% | 81% | 50% | 81% |
| Hunan | 27% | 43% | 65% | 27% | 43% | 65% | 27% | 43% | 65% | 28% | 42% | 63% | 28% | 63% | 28% | 42% | 63% | 26% | 66% |
| Inner Mongolia | 12% | 40% | 81% | 12% | 40% | 81% | 12% | 40% | 81% | 12% | 40% | 81% | 12% | 81% | 12% | 40% | 81% | 12% | 81% |
| Jiangsu | 22% | 37% | 63% | 22% | 37% | 63% | 22% | 37% | 63% | 22% | 37% | 62% | 22% | 62% | 22% | 37% | 62% | 20% | 65% |
| Jiangxi | 21% | 33% | 58% | 21% | 33% | 58% | 21% | 33% | 58% | 21% | 32% | 58% | 21% | 58% | 21% | 32% | 58% | 20% | 60% |
| Jilin | 19% | 30% | 56% | 19% | 30% | 56% | 19% | 30% | 56% | 19% | 30% | 56% | 19% | 56% | 19% | 30% | 56% | 18% | 57% |
| Liaoning | 18% | 26% | 47% | 18% | 26% | 47% | 18% | 26% | 47% | 19% | 25% | 46% | 19% | 46% | 19% | 25% | 46% | 18% | 48% |
| Ningxia | 19% | 31% | 58% | 19% | 31% | 58% | 19% | 31% | 58% | 19% | 31% | 57% | 19% | 57% | 19% | 31% | 57% | 19% | 58% |
| Qinghai | 30% | 57% | 81% | 30% | 57% | 81% | 30% | 57% | 81% | 30% | 57% | 81% | 30% | 81% | 30% | 57% | 81% | 30% | 81% |
| Shaanxi | 18% | 32% | 62% | 18% | 32% | 62% | 18% | 32% | 62% | 19% | 31% | 60% | 19% | 60% | 19% | 31% | 60% | 16% | 66% |
| Shandong | 27% | 37% | 56% | 27% | 37% | 56% | 27% | 37% | 56% | 29% | 37% | 51% | 29% | 51% | 29% | 37% | 51% | 26% | 57% |
| Shanghai | 26% | 39% | 58% | 26% | 39% | 58% | 26% | 39% | 58% | 26% | 39% | 58% | 26% | 58% | 26% | 39% | 58% | 26% | 59% |
| Shanxi | 23% | 33% | 55% | 23% | 33% | 55% | 23% | 33% | 55% | 25% | 33% | 51% | 25% | 51% | 25% | 33% | 51% | 22% | 56% |
| Sichuan | 21% | 38% | 66% | 21% | 38% | 66% | 21% | 38% | 66% | 21% | 38% | 66% | 21% | 66% | 21% | 38% | 66% | 21% | 67% |
| Tianjin | 29% | 37% | 51% | 29% | 37% | 51% | 29% | 37% | 51% | 29% | 37% | 50% | 29% | 50% | 29% | 37% | 50% | 29% | 51% |
| Tibet | 43% | 65% | 74% | 43% | 65% | 74% | 43% | 65% | 74% | 48% | 66% | 72% | 48% | 72% | 48% | 66% | 72% | 43% | 75% |
| Xinjiang | 32% | 38% | 48% | 32% | 38% | 48% | 32% | 38% | 48% | 32% | 38% | 48% | 32% | 48% | 32% | 38% | 48% | 32% | 48% |
| Yunnan | 23% | 42% | 68% | 23% | 42% | 68% | 23% | 42% | 68% | 24% | 42% | 66% | 24% | 66% | 24% | 42% | 66% | 22% | 69% |
| Zhejiang | 32% | 38% | 52% | 32% | 38% | 52% | 32% | 38% | 52% | 32% | 38% | 52% | 32% | 52% | 32% | 38% | 52% | 32% | 52% |

**S2 Table. The percentage of EV71-HFMD cases among all mild HFMD cases in each province in each of the 19 test-negative scenarios.**

| **Province** | **Percentage of EV71-HFMD among severe/fatal test-negatives** | | | | | | | | | | | | | | | | | | |
| --- | --- | --- | --- | --- | --- | --- | --- | --- | --- | --- | --- | --- | --- | --- | --- | --- | --- | --- | --- |
|  | **A** | **B** | **C** | **D** | **E** | **F** | **G** | **H** | **I** | **J** | **K** | **L** | **M** | **N** | **O** | **P** | **Q** | **R** | **S** |
| Anhui | 0% | 0% | 0% | 86% | 86% | 86% | 100% | 100% | 100% | 0% | 0% | 0% | 85% | 85% | 100% | 100% | 100% | 0% | 0% |
| Beijing | 0% | 0% | 0% | 60% | 60% | 60% | 100% | 100% | 100% | 0% | 0% | 0% | 62% | 62% | 100% | 100% | 100% | 0% | 0% |
| Chongqing | 0% | 0% | 0% | 71% | 71% | 71% | 100% | 100% | 100% | 0% | 0% | 0% | 71% | 71% | 100% | 100% | 100% | 0% | 0% |
| Fujian | 0% | 0% | 0% | 77% | 77% | 77% | 100% | 100% | 100% | 0% | 0% | 0% | 78% | 78% | 100% | 100% | 100% | 0% | 0% |
| Gansu | 0% | 0% | 0% | 79% | 79% | 79% | 100% | 100% | 100% | 0% | 0% | 0% | 79% | 79% | 100% | 100% | 100% | 0% | 0% |
| Guangdong | 0% | 0% | 0% | 79% | 79% | 79% | 100% | 100% | 100% | 0% | 0% | 0% | 79% | 79% | 100% | 100% | 100% | 0% | 0% |
| Guangxi | 0% | 0% | 0% | 68% | 68% | 68% | 100% | 100% | 100% | 0% | 0% | 0% | 73% | 73% | 100% | 100% | 100% | 0% | 0% |
| Guizhou | 0% | 0% | 0% | 58% | 58% | 58% | 100% | 100% | 100% | 0% | 0% | 0% | 58% | 58% | 100% | 100% | 100% | 0% | 0% |
| Hainan | 0% | 0% | 0% | 79% | 79% | 79% | 100% | 100% | 100% | 0% | 0% | 0% | 80% | 80% | 100% | 100% | 100% | 0% | 0% |
| Hebei | 0% | 0% | 0% | 80% | 80% | 80% | 100% | 100% | 100% | 0% | 0% | 0% | 80% | 80% | 100% | 100% | 100% | 0% | 0% |
| Heilongjiang | 0% | 0% | 0% | 86% | 86% | 86% | 100% | 100% | 100% | 0% | 0% | 0% | 86% | 86% | 100% | 100% | 100% | 0% | 0% |
| Henan | 0% | 0% | 0% | 83% | 83% | 83% | 100% | 100% | 100% | 0% | 0% | 0% | 84% | 84% | 100% | 100% | 100% | 0% | 0% |
| Hubei | 0% | 0% | 0% | 89% | 89% | 89% | 100% | 100% | 100% | 0% | 0% | 0% | 89% | 89% | 100% | 100% | 100% | 0% | 0% |
| Hunan | 0% | 0% | 0% | 72% | 72% | 72% | 100% | 100% | 100% | 0% | 0% | 0% | 73% | 73% | 100% | 100% | 100% | 0% | 0% |
| Inner Mongolia | 0% | 0% | 0% | 79% | 79% | 79% | 100% | 100% | 100% | 0% | 0% | 0% | 79% | 79% | 100% | 100% | 100% | 0% | 0% |
| Jiangsu | 0% | 0% | 0% | 73% | 73% | 73% | 100% | 100% | 100% | 0% | 0% | 0% | 74% | 74% | 100% | 100% | 100% | 0% | 0% |
| Jiangxi | 0% | 0% | 0% | 82% | 82% | 82% | 100% | 100% | 100% | 0% | 0% | 0% | 82% | 82% | 100% | 100% | 100% | 0% | 0% |
| Jilin | 0% | 0% | 0% | 78% | 78% | 78% | 100% | 100% | 100% | 0% | 0% | 0% | 78% | 78% | 100% | 100% | 100% | 0% | 0% |
| Liaoning | 0% | 0% | 0% | 70% | 70% | 70% | 100% | 100% | 100% | 0% | 0% | 0% | 65% | 65% | 100% | 100% | 100% | 0% | 0% |
| Ningxia | 0% | 0% | 0% | 85% | 85% | 85% | 100% | 100% | 100% | 0% | 0% | 0% | 82% | 82% | 100% | 100% | 100% | 0% | 0% |
| Qinghai | 0% | 0% | 0% | 0% | 0% | 0% | 100% | 100% | 100% | 0% | 0% | 0% | 0% | 0% | 100% | 100% | 100% | 0% | 0% |
| Shaanxi | 0% | 0% | 0% | 72% | 72% | 72% | 100% | 100% | 100% | 0% | 0% | 0% | 72% | 72% | 100% | 100% | 100% | 0% | 0% |
| Shandong | 0% | 0% | 0% | 69% | 69% | 69% | 100% | 100% | 100% | 0% | 0% | 0% | 71% | 71% | 100% | 100% | 100% | 0% | 0% |
| Shanghai | 0% | 0% | 0% | 95% | 95% | 95% | 100% | 100% | 100% | 0% | 0% | 0% | 95% | 95% | 100% | 100% | 100% | 0% | 0% |
| Shanxi | 0% | 0% | 0% | 86% | 86% | 86% | 100% | 100% | 100% | 0% | 0% | 0% | 89% | 89% | 100% | 100% | 100% | 0% | 0% |
| Sichuan | 0% | 0% | 0% | 67% | 67% | 67% | 100% | 100% | 100% | 0% | 0% | 0% | 67% | 67% | 100% | 100% | 100% | 0% | 0% |
| Tianjin | 0% | 0% | 0% | 75% | 75% | 75% | 100% | 100% | 100% | 0% | 0% | 0% | 93% | 93% | 100% | 100% | 100% | 0% | 0% |
| Tibet | 0% | 0% | 0% | 100% | 100% | 100% | 100% | 100% | 100% | 0% | 0% | 0% | 100% | 100% | 100% | 100% | 100% | 0% | 0% |
| Xinjiang | 0% | 0% | 0% | 94% | 94% | 94% | 100% | 100% | 100% | 0% | 0% | 0% | 84% | 84% | 100% | 100% | 100% | 0% | 0% |
| Yunnan | 0% | 0% | 0% | 85% | 85% | 85% | 100% | 100% | 100% | 0% | 0% | 0% | 84% | 84% | 100% | 100% | 100% | 0% | 0% |
| Zhejiang | 0% | 0% | 0% | 61% | 61% | 61% | 100% | 100% | 100% | 0% | 0% | 0% | 61% | 61% | 100% | 100% | 100% | 0% | 0% |

**S3 Table. The percentage of EV71-HFMD cases among severe/fatal test-negative cases in each province in each of the 19 test-negative scenarios.**

| **Province** | **Percentage of EV71-HFMD among mild test-negative cases** | | | | | | | | | | | | | | | | | | |
| --- | --- | --- | --- | --- | --- | --- | --- | --- | --- | --- | --- | --- | --- | --- | --- | --- | --- | --- | --- |
|  | **A** | **B** | **C** | **D** | **E** | **F** | **G** | **H** | **I** | **J** | **K** | **L** | **M** | **N** | **O** | **P** | **Q** | **R** | **S** |
| Anhui | 0% | 51% | 100% | 0% | 51% | 100% | 0% | 51% | 100% | 0% | 52% | 100% | 0% | 100% | 0% | 52% | 100% | 0% | 100% |
| Beijing | 0% | 37% | 100% | 0% | 37% | 100% | 0% | 37% | 100% | 0% | 36% | 100% | 0% | 100% | 0% | 36% | 100% | 0% | 100% |
| Chongqing | 0% | 31% | 100% | 0% | 31% | 100% | 0% | 31% | 100% | 0% | 31% | 100% | 0% | 100% | 0% | 31% | 100% | 0% | 100% |
| Fujian | 0% | 46% | 100% | 0% | 46% | 100% | 0% | 46% | 100% | 0% | 46% | 100% | 0% | 100% | 0% | 46% | 100% | 0% | 100% |
| Gansu | 0% | 36% | 100% | 0% | 36% | 100% | 0% | 36% | 100% | 0% | 36% | 100% | 0% | 100% | 0% | 36% | 100% | 0% | 100% |
| Guangdong | 0% | 31% | 100% | 0% | 31% | 100% | 0% | 31% | 100% | 0% | 31% | 100% | 0% | 100% | 0% | 31% | 100% | 0% | 100% |
| Guangxi | 0% | 23% | 100% | 0% | 23% | 100% | 0% | 23% | 100% | 0% | 18% | 100% | 0% | 100% | 0% | 18% | 100% | 0% | 100% |
| Guizhou | 0% | 32% | 100% | 0% | 32% | 100% | 0% | 32% | 100% | 0% | 32% | 100% | 0% | 100% | 0% | 32% | 100% | 0% | 100% |
| Hainan | 0% | 23% | 100% | 0% | 23% | 100% | 0% | 23% | 100% | 0% | 22% | 100% | 0% | 100% | 0% | 22% | 100% | 0% | 100% |
| Hebei | 0% | 50% | 100% | 0% | 50% | 100% | 0% | 50% | 100% | 0% | 50% | 100% | 0% | 100% | 0% | 50% | 100% | 0% | 100% |
| Heilongjiang | 0% | 49% | 100% | 0% | 49% | 100% | 0% | 49% | 100% | 0% | 49% | 100% | 0% | 100% | 0% | 49% | 100% | 0% | 100% |
| Henan | 0% | 47% | 100% | 0% | 47% | 100% | 0% | 47% | 100% | 0% | 34% | 100% | 0% | 100% | 0% | 34% | 100% | 0% | 100% |
| Hubei | 0% | 72% | 100% | 0% | 72% | 100% | 0% | 72% | 100% | 0% | 72% | 100% | 0% | 100% | 0% | 72% | 100% | 0% | 100% |
| Hunan | 0% | 42% | 100% | 0% | 42% | 100% | 0% | 42% | 100% | 0% | 40% | 100% | 0% | 100% | 0% | 40% | 100% | 0% | 100% |
| Inner Mongolia | 0% | 41% | 100% | 0% | 41% | 100% | 0% | 41% | 100% | 0% | 41% | 100% | 0% | 100% | 0% | 41% | 100% | 0% | 100% |
| Jiangsu | 0% | 36% | 100% | 0% | 36% | 100% | 0% | 36% | 100% | 0% | 36% | 100% | 0% | 100% | 0% | 36% | 100% | 0% | 100% |
| Jiangxi | 0% | 32% | 100% | 0% | 32% | 100% | 0% | 32% | 100% | 0% | 31% | 100% | 0% | 100% | 0% | 31% | 100% | 0% | 100% |
| Jilin | 0% | 30% | 100% | 0% | 30% | 100% | 0% | 30% | 100% | 0% | 30% | 100% | 0% | 100% | 0% | 30% | 100% | 0% | 100% |
| Liaoning | 0% | 26% | 100% | 0% | 26% | 100% | 0% | 26% | 100% | 0% | 26% | 100% | 0% | 100% | 0% | 26% | 100% | 0% | 100% |
| Ningxia | 0% | 32% | 100% | 0% | 32% | 100% | 0% | 32% | 100% | 0% | 31% | 100% | 0% | 100% | 0% | 31% | 100% | 0% | 100% |
| Qinghai | 0% | 52% | 100% | 0% | 52% | 100% | 0% | 52% | 100% | 0% | 52% | 100% | 0% | 100% | 0% | 52% | 100% | 0% | 100% |
| Shaanxi | 0% | 31% | 100% | 0% | 31% | 100% | 0% | 31% | 100% | 0% | 28% | 100% | 0% | 100% | 0% | 28% | 100% | 0% | 100% |
| Shandong | 0% | 37% | 100% | 0% | 37% | 100% | 0% | 37% | 100% | 0% | 37% | 100% | 0% | 100% | 0% | 37% | 100% | 0% | 100% |
| Shanghai | 0% | 41% | 100% | 0% | 41% | 100% | 0% | 41% | 100% | 0% | 41% | 100% | 0% | 100% | 0% | 41% | 100% | 0% | 100% |
| Shanxi | 0% | 33% | 100% | 0% | 33% | 100% | 0% | 33% | 100% | 0% | 30% | 100% | 0% | 100% | 0% | 30% | 100% | 0% | 100% |
| Sichuan | 0% | 39% | 100% | 0% | 39% | 100% | 0% | 39% | 100% | 0% | 39% | 100% | 0% | 100% | 0% | 39% | 100% | 0% | 100% |
| Tianjin | 0% | 40% | 100% | 0% | 40% | 100% | 0% | 40% | 100% | 0% | 39% | 100% | 0% | 100% | 0% | 39% | 100% | 0% | 100% |
| Tibet | 0% | 70% | 100% | 0% | 70% | 100% | 0% | 70% | 100% | 0% | 75% | 100% | 0% | 100% | 0% | 75% | 100% | 0% | 100% |
| Xinjiang | 0% | 38% | 100% | 0% | 38% | 100% | 0% | 38% | 100% | 0% | 38% | 100% | 0% | 100% | 0% | 38% | 100% | 0% | 100% |
| Yunnan | 0% | 42% | 100% | 0% | 42% | 100% | 0% | 42% | 100% | 0% | 43% | 100% | 0% | 100% | 0% | 43% | 100% | 0% | 100% |
| Zhejiang | 0% | 31% | 100% | 0% | 31% | 100% | 0% | 31% | 100% | 0% | 31% | 100% | 0% | 100% | 0% | 31% | 100% | 0% | 100% |

**S4 Table. The percentage of EV71-HFMD cases among mild test-negative cases in each province in each of the 19 test-negative scenarios.**

|  | Societal perspective | | | | Excluding productivity loss of parents/caregivers | | | |
| --- | --- | --- | --- | --- | --- | --- | --- | --- |
|  | 3% discounting | | 6% discounting | | 3% discounting | | 6% discounting | |
|  | WTP 1 GDPpc | WTP 3 GDPpc | WTP 1 GDPpc | WTP 3 GDPpc | WTP 1 GDPpc | WTP 3 GDPpc | WTP 1 GDPpc | WTP 3 GDPpc |
| A  (Base case) | 0% [0%, 0%] | 81% [83%, 79%] | -18% [-19%, -18%] | 32% [33%, 31%] | -10% [-10%, -11%] | 71% [73%, 69%] | -28% [-29%, -28%] | 22% [23%, 21%] |
| B | 23% [22%, 24%] | 69% [72%, 67%] | -16% [-17%, -15%] | 28% [29%, 27%] | -12% [-12%, -13%] | 57% [60%, 54%] | -28% [-28%, -27%] | 16% [18%, 15%] |
| C | 57% [55%, 59%] | 58% [61%, 56%] | -14% [-14%, -13%] | 24% [26%, 23%] | -14% [-14%, -14%] | 44% [47%, 41%] | -27% [-27%, -27%] | 11% [13%, 10%] |
| D | 6% [7%, 6%] | 83% [85%, 81%] | -19% [-19%, -18%] | 32% [33%, 31%] | -10% [-10%, -10%] | 73% [75%, 71%] | -28% [-28%, -28%] | 23% [24%, 22%] |
| E | 29% [29%, 30%] | 71% [74%, 69%] | -16% [-17%, -16%] | 28% [30%, 27%] | -12% [-12%, -12%] | 59% [62%, 57%] | -28% [-28%, -27%] | 17% [19%, 16%] |
| F | 63% [62%, 65%] | 60% [63%, 58%] | -14% [-14%, -13%] | 25% [26%, 24%] | -14% [-13%, -14%] | 46% [49%, 44%] | -27% [-27%, -27%] | 12% [14%, 11%] |
| G | 8% [9%, 8%] | 83% [85%, 81%] | -19% [-19%, -18%] | 32% [33%, 31%] | -10% [-10%, -10%] | 73% [75%, 71%] | -28% [-28%, -28%] | 23% [24%, 22%] |
| H | 31% [31%, 32%] | 72% [74%, 69%] | -16% [-17%, -16%] | 29% [30%, 27%] | -12% [-11%, -12%] | 60% [63%, 58%] | -28% [-28%, -27%] | 17% [19%, 16%] |
| I | 65% [64%, 67%] | 61% [63%, 58%] | -14% [-15%, -14%] | 25% [27%, 24%] | -14% [-13%, -14%] | 47% [50%, 44%] | -27% [-27%, -27%] | 12% [14%, 11%] |
| J | 1% [1%, 1%] | 79% [81%, 76%] | -18% [-18%, -17%] | 31% [32%, 30%] | -11% [-11%, -11%] | 68% [71%, 66%] | -28% [-28%, -28%] | 21% [22%, 19%] |
| K | 20% [19%, 21%] | 69% [72%, 67%] | -16% [-16%, -15%] | 28% [29%, 27%] | -13% [-12%, -13%] | 56% [59%, 54%] | -28% [-28%, -27%] | 16% [17%, 15%] |
| L | 50% [48%, 52%] | 59% [62%, 57%] | -14% [-14%, -13%] | 25% [26%, 24%] | -14% [-14%, -15%] | 45% [48%, 42%] | -27% [-28%, -27%] | 11% [13%, 10%] |
| M | 10% [10%, 10%] | 80% [83%, 78%] | -18% [-19%, -18%] | 31% [33%, 30%] | -10% [-10%, -10%] | 70% [73%, 68%] | -28% [-28%, -28%] | 22% [23%, 20%] |
| N | 59% [58%, 61%] | 61% [64%, 59%] | -14% [-15%, -14%] | 25% [27%, 24%] | -14% [-13%, -14%] | 48% [51%, 45%] | -27% [-27%, -27%] | 13% [14%, 11%] |
| O | 13% [13%, 12%] | 81% [83%, 79%] | -18% [-19%, -18%] | 31% [33%, 30%] | -10% [-10%, -10%] | 71% [73%, 69%] | -28% [-28%, -27%] | 22% [23%, 21%] |
| P | 32% [31%, 32%] | 72% [74%, 69%] | -16% [-17%, -16%] | 28% [30%, 27%] | -12% [-11%, -12%] | 60% [63%, 58%] | -27% [-28%, -27%] | 17% [19%, 16%] |
| Q | 62% [60%, 63%] | 62% [65%, 59%] | -14% [-15%, -14%] | 26% [27%, 24%] | -13% [-13%, -14%] | 48% [51%, 46%] | -27% [-27%, -27%] | 13% [14%, 11%] |
| R | 4% [4%, 3%] | 84% [86%, 82%] | -19% [-20%, -19%] | 33% [34%, 32%] | -10% [-9%, -10%] | 75% [77%, 73%] | -28% [-29%, -28%] | 23% [25%, 22%] |
| S | 66% [64%, 68%] | 59% [62%, 57%] | -14% [-14%, -13%] | 25% [26%, 24%] | -14% [-13%, -14%] | 46% [49%, 43%] | -27% [-27%, -27%] | 12% [13%, 10%] |

**S5 Table. Changes in** *EVC_max_* **across the scenarios considered in Table 2.** In the first column (i.e. societal perspective, 3% discount rate, willingness-to-pay threshold of 1 × GDPpc), the point estimates and 95% confidence intervals of *EVC_max_* in scenarios B-S were compared to that in the base case (i.e. scenario A). For the remaining columns, the point estimates and 95% confidence intervals of *EVC_max_* in each scenario were compared to their counterparts in the first column, i.e. *EVC_max_* in scenario *X* of column *Y* was compared to *EVC_max_* in scenario *X* of column 1.

| **Region** | **Province composition** |
| --- | --- |
| Northeast | Heilongjiang, Jilin, Liaoning |
| East | Shandong, Jiangsu, Anhui, Jiangxi, Zhejiang, Fujian, Shanghai |
| South | Guangxi, Guangdong, Hainan |
| Central | Hubei, Hunan, Henan |
| North | Beijing, Tianjin, Hebei, Shanxi, Inner Mongolia |
| Northwest | Ningxia, Xinjiang, Qinghai, Shanxi, Gansu |
| Southwest | Sichuan, Yunnan, Guizhou, Xizang, Chongqing |

**S6 Table. Seven regions in China from which equal representation in the sample of our telephone survey was obtained.**

|  | | **Mild outpatient** | **Mild inpatient** | **Severe** | **Fatal** |
| --- | --- | --- | --- | --- | --- |
| **Total** | | 451 | 484 | 798 | 54 |
| **Gender** | **Male** | 297 (66%) | 318 (66%) | 536 (67%) | 30 (63%) |
|  | **Female** | 154 (34%) | 166 (34%) | 262 (33%) | 24 (37%) |
| **Urban/rural** | **Urban** | 186 (41%) | 292 (60%) | 443 (56%) | 26 (48%) |
|  | **Rural** | 265 (59%) | 192 (40%) | 355 (44%) | 28 (52%) |
| **Median age (2.5% - 97.5%)** | | 2.77 (0.88 – 4.87) | 2.39 (0.82 – 4.99) | 2.21 (0.98 – 4.58) | 2.03 (0.55 – 4.99) |
| **Duration of illness (2.5% - 97.5%)** | | 7.0 (2.0 – 15.0) | 7.0 (4.0 – 20.0) | 10.0 (5.0 – 30.0) | 6.0 (1.0 – 21.6) |
| **Geographical region** | **Northeast** | 65 (14%) | 45 (9%) | 140 (18%) | 3 (6%) |
|  | **East** | 69 (15%) | 89 (18%) | 11 (1%) | 10 (19%) |
|  | **South** | 57 (13%) | 126 (26%) | 61 (8%) | 2 (4%) |
|  | **Central** | 70 (16%) | 64 (13%) | 119 (15%) | 7 (13%) |
|  | **North** | 65 (14%) | 55 (11%) | 159 (20%) | 5 (9%) |
|  | **Northwest** | 64 (14%) | 54 (11%) | 164 (21%) | 16 (30%) |
|  | **Southwest** | 61 (14%) | 51 (11%) | 144 (18%) | 11 (20%) |

**S7 Table. Demographic characteristic and geographic distribution of 1,787 EV71-HFMD patients whose parents or caregivers were telephone survey participants**

|  | | **Mild outpatient** | **Mild inpatient** | **Severe** | **Fatal** |
| --- | --- | --- | --- | --- | --- |
| **Overall** | | 185 | 1400 | 3170 | 2738 |
| **Gender** | **Male** | 190 | 1417 | 3126 | 3326 |
|  | **Female** | 173 | 1369 | 3259 | 2003 |
| **Urban/rural** | **Urban** | 196 | 1284 | 3097 | 2338 |
|  | **Rural** | 168 | 1476 | 3228 | 3168 |
| **Age group** | **6 mo – 1 yr** | 208 | 1207 | 3133 | 3758 |
|  | **1 – 2 yrs** | 204 | 1517 | 3221 | 3359 |
|  | **2 – 3 yrs** | 193 | 1532 | 3318 | 2311 |
|  | **3 – 4 yrs** | 164 | 1211 | 2919 | 2000 |
|  | **4 – 5 yrs** | 154 | 1264 | 2985 | 1805 |
| **Geographical region** | **Northeast** | 143 | 1380 | 2999 | Not stratified |
|  | **East** | 239 | 1011 | 3592 |  |
|  | **South** | 200 | 2168 | 3085 |  |
|  | **Central** | 260 | 1145 | 3122 |  |
|  | **North** | 141 | 1669 | 4077 |  |
|  | **Northwest** | 143 | 1081 | 2655 |  |
|  | **Southwest** | 156 | 569 | 2964 |  |

**S8 Table. Costs for 1,787 EV71-HFMD patients whose parents or caregivers were telephone survey participants (mean, in US dollars)**

|  | | **Mild outpatient** | **Mild inpatient** | **Severe** | **Fatal** |
| --- | --- | --- | --- | --- | --- |
| **Overall** | | 0.0036 | 0.0082 | 0.0149 | 0.0111 |
| **Gender** | **Male** | 0.0033 | 0.0082 | 0.0143 | 0.0092 |
|  | **Female** | 0.0040 | 0.0082 | 0.0162 | 0.0136 |
| **Urban/rural** | **Urban** | 0.0034 | 0.0075 | 0.0134 | 0.0117 |
|  | **Rural** | 0.0038 | 0.0087 | 0.0162 | 0.0105 |
| **Age group** | **6 mo – 1 yr** | 0.0034 | 0.0064 | 0.0120 | 0.0114 |
|  | **1 – 2 yrs** | 0.0038 | 0.0081 | 0.0137 | 0.0078 |
|  | **2 – 3 yrs** | 0.0040 | 0.0087 | 0.0165 | 0.0093 |
|  | **3 – 4 yrs** | 0.0029 | 0.0087 | 0.0152 | 0.0176 |
|  | **4 – 5 yrs** | 0.0031 | 0.0078 | 0.0150 | 0.0155 |
| **Geographical region** | **Northeast** | 0.0034 | 0.0092 | 0.0141 | Not stratified |
|  | **East** | 0.0043 | 0.0064 | 0.0332 |  |
|  | **South** | 0.0031 | 0.0105 | 0.0135 |  |
|  | **Central** | 0.0038 | 0.0064 | 0.0143 |  |
|  | **North** | 0.0032 | 0.0100 | 0.0168 |  |
|  | **Northwest** | 0.0036 | 0.0079 | 0.0146 |  |
|  | **Southwest** | 0.0035 | 0.0055 | 0.0138 |  |

**S9 Table. QALY loss during illness for 1,787 EV71-HFMD patients whose parents or caregivers were telephone survey participants (mean)**

| **Province** | **Urban average annual income in 2013 (USD)** | **Rural average annual income in 2013 (USD)** | **Weighted average annual income in 2013 (USD)** | **Percentage of mild cases hospitalized** |
| --- | --- | --- | --- | --- |
| Beijing | 7265 | 2943 | 6668 | 0.4% |
| Tianjin | 5722 | 2542 | 5134 | 0.4% |
| Hebei | 3874 | 1461 | 2590 | 6.1% |
| Shanxi | 3854 | 1148 | 2535 | 0.8% |
| Inner Mongolia | 4329 | 1379 | 3083 | 3.7% |
| Liaoning | 4478 | 1689 | 3520 | 0.3% |
| Jilin | 3778 | 1544 | 2744 | 2.9% |
| Heilongjiang | 3394 | 1546 | 2598 | 3.2% |
| Shanghai | 7844 | 3144 | 7340 | 1.4% |
| Jiangsu | 5638 | 2182 | 4359 | 2.3% |
| Zhejiang | 6618 | 2585 | 5133 | 1.9% |
| Anhui | 4013 | 1300 | 2561 | 7.0% |
| Fujian | 5357 | 1795 | 3918 | 2.8% |
| Jiangxi | 3683 | 1409 | 2489 | 13.1% |
| Shandong | 4915 | 1704 | 3388 | 39.2% |
| Henan | 3801 | 1360 | 2396 | 34.9% |
| Hubei | 4041 | 1423 | 2824 | 5.5% |
| Hunan | 3955 | 1343 | 2561 | 2.5% |
| Guangdong | 5858 | 1873 | 4559 | 1.0% |
| Guangxi | 4016 | 1090 | 2364 | 4.0% |
| Hainan | 3999 | 1339 | 2711 | 2.2% |
| Chongqing | 4309 | 1337 | 3030 | 0.7% |
| Sichuan | 3834 | 1267 | 2385 | 5.4% |
| Guizhou | 3436 | 872 | 1806 | 2.9% |
| Yunnan | 3963 | 985 | 2156 | 2.3% |
| Tibet | 3620 | 1056 | 1639 | 1.8% |
| Shaanxi | 3869 | 1044 | 2457 | 6.9% |
| Gansu | 3233 | 820 | 1755 | 1.5% |
| Qinghai | 3551 | 994 | 2208 | 0.0% |
| Ningxia | 3814 | 1112 | 2482 | 0.1% |
| Xinjiang | 3593 | 1171 | 2236 | 1.6% |

**S10 Table. Average annual income in 2013 (urban income, rural income and income weighted by urban and rural population) and the percentage of mild HFMD cases that were inpatients in each of the 31 provinces.**

| **Variable** | **Severity** | **Region** | **Cost p-value** | **QALY loss p-value** | **Adjusted cost p-value** | **Adjusted QALY loss p-value** |
| --- | --- | --- | --- | --- | --- | --- |
| Gender | Mild outpatient | Northeast | 0.962 | 0.887 | 0.962 | 0.977 |
|  |  | East | 0.224 | 0.922 | 0.523 | 0.977 |
|  |  | South | 0.219 | 0.411 | 0.523 | 0.873 |
|  |  | Central | 0.560 | 0.499 | 0.785 | 0.873 |
|  |  | North | 0.320 | 0.144 | 0.559 | 0.873 |
|  |  | Northwest | 0.774 | 0.977 | 0.903 | 0.977 |
|  |  | Southwest | 0.082 | 0.446 | 0.523 | 0.873 |
|  | Mild inpatient | Northeast | 0.009 | 0.073 | 0.062 | 0.512 |
|  |  | East | 0.868 | 0.709 | 0.868 | 0.832 |
|  |  | South | 0.150 | 0.836 | 0.325 | 0.836 |
|  |  | Central | 0.592 | 0.589 | 0.690 | 0.832 |
|  |  | North | 0.097 | 0.232 | 0.325 | 0.541 |
|  |  | Northwest | 0.186 | 0.223 | 0.325 | 0.541 |
|  |  | Southwest | 0.529 | 0.713 | 0.690 | 0.832 |
|  | Severe | Northeast | 0.051 | 0.127 | 0.177 | 0.320 |
|  |  | East | 0.100 | 0.714 | 0.192 | 0.823 |
|  |  | South | 0.452 | 0.823 | 0.501 | 0.823 |
|  |  | Central | 0.501 | 0.050 | 0.501 | 0.320 |
|  |  | North | 0.011 | 0.200 | 0.079 | 0.349 |
|  |  | Northwest | 0.110 | 0.137 | 0.192 | 0.320 |
|  |  | Southwest | 0.162 | 0.548 | 0.227 | 0.768 |
|  | Fatal | Nationwide | 0.076 | 0.116 | 0.076 | 0.116 |
| Age | Mild outpatient | Northeast | 0.572 | 0.210 | 0.672 | 0.446 |
|  |  | East | 0.445 | 0.415 | 0.672 | 0.582 |
|  |  | South | 0.072 | 0.076 | 0.271 | 0.276 |
|  |  | Central | 0.124 | 0.754 | 0.290 | 0.880 |
|  |  | North | 0.077 | 0.255 | 0.271 | 0.446 |
|  |  | Northwest | 0.868 | 0.079 | 0.868 | 0.276 |
|  |  | Southwest | 0.576 | 0.916 | 0.672 | 0.916 |
|  | Mild inpatient | Northeast | 0.925 | 0.574 | 0.925 | 0.850 |
|  |  | East | 0.108 | 0.752 | 0.318 | 0.850 |
|  |  | South | 0.437 | 0.386 | 0.612 | 0.850 |
|  |  | Central | 0.018 | 0.451 | 0.129 | 0.850 |
|  |  | North | 0.866 | 0.817 | 0.925 | 0.850 |
|  |  | Northwest | 0.277 | 0.850 | 0.485 | 0.850 |
|  |  | Southwest | 0.136 | 0.099 | 0.318 | 0.693 |
|  | Severe | Northeast | 0.462 | 0.025 | 0.992 | 0.178 |
|  |  | East | 0.604 | 0.204 | 0.992 | 0.475 |
|  |  | South | 0.156 | 0.633 | 0.545 | 0.948 |
|  |  | Central | 0.992 | 0.753 | 0.992 | 0.948 |
|  |  | North | 0.968 | 0.830 | 0.992 | 0.948 |
|  |  | Northwest | 0.131 | 0.948 | 0.545 | 0.948 |
|  |  | Southwest | 0.760 | 0.070 | 0.992 | 0.244 |
|  | Fatal | Nationwide | 0.855 | 0.674 | 0.855 | 0.674 |
| Urban residence status | Mild outpatient | Northeast | 0.133 | 0.058 | 0.464 | 0.175 |
|  |  | East | 0.757 | 0.079 | 0.922 | 0.175 |
|  |  | South | 0.909 | 0.100 | 0.922 | 0.175 |
|  |  | Central | 0.554 | 0.016 | 0.922 | 0.110 |
|  |  | North | 0.922 | 0.284 | 0.922 | 0.331 |
|  |  | Northwest | 0.109 | 0.284 | 0.464 | 0.331 |
|  |  | Southwest | 0.302 | 0.611 | 0.704 | 0.611 |
|  | Mild inpatient | Northeast | 0.007 | 0.852 | 0.050 | 0.852 |
|  |  | East | 0.625 | 0.348 | 0.724 | 0.590 |
|  |  | South | 0.177 | 0.652 | 0.457 | 0.761 |
|  |  | Central | 0.361 | 0.155 | 0.505 | 0.362 |
|  |  | North | 0.262 | 0.144 | 0.459 | 0.362 |
|  |  | Northwest | 0.724 | 0.421 | 0.724 | 0.590 |
|  |  | Southwest | 0.196 | 0.060 | 0.457 | 0.362 |
|  | Severe | Northeast | 0.642 | 0.820 | 0.642 | 0.952 |
|  |  | East | 0.100 | 0.271 | 0.234 | 0.949 |
|  |  | South | 0.558 | 0.918 | 0.642 | 0.952 |
|  |  | Central | 0.599 | 0.653 | 0.642 | 0.952 |
|  |  | North | 0.062 | 0.033 | 0.217 | 0.233 |
|  |  | Northwest | 0.016 | 0.952 | 0.114 | 0.952 |
|  |  | Southwest | 0.300 | 0.835 | 0.524 | 0.952 |
|  | Fatal | Nationwide | 0.213 | 0.294 | 0.213 | 0.294 |

**S11 Table. Association ofcosts and QALY loss with age, gender and urban residence status.** A Kruskal–Wallis test was performed in each severity-region stratum. Multiple testing of the same hypothesis in the 7 different regions was corrected for using false discovery rate control. Associations with *p* < 0.05 are highlighted.

|  | **Region** | ***M_Q_s,d_***  (10^-3^) | ***V_Q_s,d_* (**10^-7^) | **Societal perspective** | | | **Excluding productivity loss** | | |
| --- | --- | --- | --- | --- | --- | --- | --- | --- | --- |
|  |  |  |  | ***M_C_s,d_*** | ***V_C_s,d_* (**10^4^) | ***CV_Cs,d_Q_s,d_*** | ***M_C_s,d_*** | ***V_C_s,d_* (**10^4^) | ***CV_Cs,d_Q_s,d_*** |
| **Mild outpatients** | Northeast | 4.04 | 6.14 | 223 | 0.080 | 0.011 | 158 | 0.048 | 0.011 |
|  | East | 3.55 | 0.94 | 244 | 0.100 | 0.002 | 164 | 0.046 | 0.001 |
|  | South | 3.38 | 4.06 | 136 | 0.017 | 0.004 | 105 | 0.009 | 0.003 |
|  | Central | 3.02 | 1.57 | 134 | 0.029 | 0.001 | 86 | 0.016 | 0.000 |
|  | North | 2.92 | 1.83 | 188 | 0.107 | 0.000 | 131 | 0.059 | 0.000 |
|  | Northwest | 3.21 | 1.70 | 134 | 0.016 | 0.002 | 102 | 0.008 | 0.001 |
|  | Southwest | 3.27 | 2.34 | 147 | 0.053 | 0.002 | 121 | 0.042 | 0.002 |
| **Mild inpatients­­** | Northeast | 6.00 | 2.51 | 941 | 0.755 | 0.015 | 874 | 0.699 | 0.014 |
|  | East | 6.13 | 7.01 | 1088 | 1.294 | 0.038 | 953 | 1.186 | 0.036 |
|  | South | 7.56 | 11.27 | 1032 | 5.334 | 0.143 | 957 | 4.499 | 0.131 |
|  | Central | 9.56 | 31.10 | 1596 | 11.004 | 0.301 | 1509 | 10.519 | 0.290 |
|  | North | 9.97 | 6.00 | 2063 | 1.690 | 0.044 | 1955 | 1.615 | 0.044 |
|  | Northwest | 8.69 | 56.68 | 1304 | 7.354 | 0.106 | 1235 | 7.329 | 0.099 |
|  | Southwest | 5.18 | 2.26 | 538 | 0.315 | 0.014 | 517 | 0.289 | 0.013 |
| **Severe** | Northeast | 31.45 | 3604.7 | 3389 | 73.880 | 13.587 | 3278 | 70.423 | 13.611 |
|  | East | 13.67 | 11.47 | 2978 | 1.695 | 0.053 | 2771 | 1.516 | 0.047 |
|  | South | 13.98 | 16.07 | 2537 | 4.286 | 0.109 | 2426 | 4.121 | 0.100 |
|  | Central | 16.18 | 10.30 | 3932 | 10.882 | 0.139 | 3815 | 10.526 | 0.134 |
|  | North | 12.95 | 14.86 | 2957 | 5.199 | 0.105 | 2837 | 4.983 | 0.100 |
|  | Northwest | 13.44 | 8.43 | 2851 | 2.090 | 0.053 | 2725 | 2.010 | 0.051 |
|  | Southwest | 13.12 | 10.32 | 2823 | 2.146 | 0.072 | 2761 | 2.124 | 0.070 |
| **Fatal** | Nationwide | 30424 | 41.62 | 2449 | 25.859 | 0.292 | 2228 | 24.713 | 0.247 |

**S12 Table. Mean, variance and covariance of our survey data on cost and QALY loss per mild outpatient, mild inpatient, severe case and fatal case of EV71-HFMD in each region.**

| **Province** | **Region** | **Expected cost (USD)**  **Societal perspective** | | | **Expected cost (USD)**  **Excluding productivity loss** | | | **Expected QALY loss** | | |
| --- | --- | --- | --- | --- | --- | --- | --- | --- | --- | --- |
|  |  | **Fatal** | **Severe** | **Mild** | **Fatal** | **Severe** | **Mild** | **Fatal** | **Severe** | **Mild** |
| Beijing | North | 2449 | 2957 | 196 | 2228 | 2837 | 138 | 30.42 | 0.0130 | 0.0029 |
| Tianjin | North | 2449 | 2957 | 196 | 2228 | 2837 | 138 | 30.42 | 0.0130 | 0.0029 |
| Hebei | North | 2449 | 2957 | 304 | 2228 | 2837 | 243 | 30.42 | 0.0130 | 0.0034 |
| Shanxi | North | 2449 | 2957 | 204 | 2228 | 2837 | 147 | 30.42 | 0.0130 | 0.0030 |
| Inner Mongolia | North | 2449 | 2957 | 258 | 2228 | 2837 | 199 | 30.42 | 0.0130 | 0.0032 |
| Liaoning | Northeast | 2449 | 3389 | 225 | 2228 | 3278 | 160 | 30.42 | 0.0315 | 0.0040 |
| Jilin | Northeast | 2449 | 3389 | 243 | 2228 | 3278 | 178 | 30.42 | 0.0315 | 0.0041 |
| Heilongjiang | Northeast | 2449 | 3389 | 246 | 2228 | 3278 | 181 | 30.42 | 0.0315 | 0.0041 |
| Shanghai | East | 2449 | 2978 | 256 | 2228 | 2771 | 175 | 30.42 | 0.0137 | 0.0036 |
| Jiangsu | East | 2449 | 2978 | 263 | 2228 | 2771 | 182 | 30.42 | 0.0137 | 0.0036 |
| Zhejiang | East | 2449 | 2978 | 260 | 2228 | 2771 | 179 | 30.42 | 0.0137 | 0.0036 |
| Anhui | East | 2449 | 2978 | 303 | 2228 | 2771 | 219 | 30.42 | 0.0137 | 0.0037 |
| Fujian | East | 2449 | 2978 | 268 | 2228 | 2771 | 186 | 30.42 | 0.0137 | 0.0036 |
| Jiangxi | East | 2449 | 2978 | 355 | 2228 | 2771 | 268 | 30.42 | 0.0137 | 0.0039 |
| Shandong | East | 2449 | 2978 | 574 | 2228 | 2771 | 473 | 30.42 | 0.0137 | 0.0046 |
| Henan | Central | 2449 | 3932 | 643 | 2228 | 3815 | 583 | 30.42 | 0.0162 | 0.0053 |
| Hubei | Central | 2449 | 3932 | 214 | 2228 | 3815 | 164 | 30.42 | 0.0162 | 0.0034 |
| Hunan | Central | 2449 | 3932 | 170 | 2228 | 3815 | 121 | 30.42 | 0.0162 | 0.0032 |
| Guangdong | South | 2449 | 2537 | 144 | 2228 | 2426 | 114 | 30.42 | 0.0140 | 0.0034 |
| Guangxi | South | 2449 | 2537 | 171 | 2228 | 2426 | 139 | 30.42 | 0.0140 | 0.0035 |
| Hainan | South | 2449 | 2537 | 156 | 2228 | 2426 | 124 | 30.42 | 0.0140 | 0.0035 |
| Chongqing | Southwest | 2449 | 2823 | 150 | 2228 | 2761 | 124 | 30.42 | 0.0131 | 0.0033 |
| Sichuan | Southwest | 2449 | 2823 | 168 | 2228 | 2761 | 143 | 30.42 | 0.0131 | 0.0034 |
| Guizhou | Southwest | 2449 | 2823 | 159 | 2228 | 2761 | 133 | 30.42 | 0.0131 | 0.0033 |
| Yunnan | Southwest | 2449 | 2823 | 156 | 2228 | 2761 | 130 | 30.42 | 0.0131 | 0.0033 |
| Tibet | Southwest | 2449 | 2823 | 154 | 2228 | 2761 | 129 | 30.42 | 0.0131 | 0.0033 |
| Shaanxi | Northwest | 2449 | 2851 | 215 | 2228 | 2725 | 179 | 30.42 | 0.0134 | 0.0036 |
| Gansu | Northwest | 2449 | 2851 | 152 | 2228 | 2725 | 119 | 30.42 | 0.0134 | 0.0033 |
| Qinghai | Northwest | 2449 | 2851 | 134 | 2228 | 2725 | 102 | 30.42 | 0.0134 | 0.0032 |
| Ningxia | Northwest | 2449 | 2851 | 135 | 2228 | 2725 | 103 | 30.42 | 0.0134 | 0.0032 |
| Xinjiang | Northwest | 2449 | 2851 | 154 | 2228 | 2725 | 120 | 30.42 | 0.0134 | 0.0033 |

**S13 Table. Expected cost and QALY loss per case of mild, severe and fatal EV71-HFMD in each of the 31 provinces.**

| **County** | **Study period** | **Age range** | **No. of subjects in placebo group** | **No. of EV71-HFMD in placebo group (per-protocol; EV71 associated diseases)** | **EV71-HFMD incidence rate**  **(per 1000 person-year)** |
| --- | --- | --- | --- | --- | --- |
| Donghai, Jiangsu  [ Lancet 2013 ] | Jan 2012 – Mar 2013 | 6 – 35 months | 1438 (Appendix 6) | 5 (Appendix 12) | 3.5 (1.1-8.1) |
| Pizhou, Jiangsu  [ Lancet 2013 ] | Jan 2012 – Mar 2013 | 6 – 35 months | 2227 (Appendix 6) | 3 (Appendix 12) | 1.3 (0.3-3.9) |
| Baoying, Jiangsu  [ Lancet 2013 ] | Jan 2012 – Mar 2013 | 6 – 35 months | 1046 (Appendix 6) | 28 (Appendix 12) | 26.8 (17.9-38.5) |
| Chaoyang, Beijing  [ Lancet 2013 ] | Jan 2012 – Mar 2013 | 6 – 35 months | 414 (Appendix 6) | 5 (Appendix 12) | 12.1(3.9-28.0) |
| Ganyu, Jiangsu  [ Zhu NEJM 2014 ] | Jan 2012 – Mar 2013 | 6 – 35 months | 2323 (S7 Table) | 19 (S11 Table) | 8.2 (4.9-12.7) |
| Sheyang Jiangsu  [Zhu NEJM 2014 ] | Jan 2012 – Mar 2013 | 6 – 35 months | 1675 (S7 Table) | 63 (S11 Table) | 37.6 (29.0-47.9) |
| Taixing, Jiangsu  [ Zhu NEJM 2014 ] | Jan 2012 – Mar 2013 | 6 – 35 months | 1035 (S7 Table) | 19 (S11 Table) | 18.4 (11.1-28.5) |
| 7 contiguous counties, Guangxi  [ Li NEJM 2014 ] | Mar 2012 – Feb 2013 | 6 – 71 months | 5499 (S2 Table) | 145 (S2 Table) | 26.4 (22.3-31.0) |
| Aggregated | Jan 2012 –Mar 2013 | 6 – 71 months | 15657 | 287 | 18.3 (16.3-20.6) |

**S14 Table. Estimated incidence rate of EV71-HFMD in the study areas of the EV71 vaccine phase III trials.**

| **County** | **Population size (2010 census)** | **No. of HFMD cases, *K_X_*,*_T_*** | **No. of test-positives** | **No. of test-positives that were EV71** | **Percentage of HFMD cases that were EV71, *R_X,T_*** | **EV71-HFMD**  **incidence rate**  **(per 1000 person-year)** | **EV71-HFMD incidence rate in national surveillance/ EV71-HFMD incidence rate in vaccine trials** |
| --- | --- | --- | --- | --- | --- | --- | --- |
| Donghai, Jiangsu | 97273 | 281 | 21 | 5 | 0.24 (0.08-0.47) | 0.7 (0.3-1.3) | 0.20 (0.05-0.99) |
| Pizhou, Jiangsu | 140184 | 273 | 3 | 1 | 0.33 (0.01-0.91) | 0.6 (0.0-1.8) | 0.48 (0.00-10.0) |
| Baoying, Jiangsu | 30721 | 568 | 41 | 11 | 0.27 (0.14-0.43) | 5.0 (2.8-7.7) | 0.19 (0.09-0.33) |
| Chaoyang, Beijing | 118625 | 2504 | 123 | 40 | 0.33 (0.24-0.42) | 6.9 (5.2-8.7) | 0.58 (0.27-2.77) |
| Ganyu, Jiangsu | 78769 | 1629 | 35 | 4 | 0.11 (0.03-0.27) | 2.4 (0.7-5.5) | 0.28 (0.06-0.69) |
| Sheyang Jiangsu | 47424 | 402 | 21 | 16 | 0.76 (0.53-0.92) | 6.5 (4.6-7.7) | 0.17 (0.12-0.24) |
| Taixing, Jiangsu | 41092 | 550 | 26 | 4 | 0.15 (0.04-0.35) | 2.1 (0.7-4.5) | 0.11 (0.03-0.26) |
| 7 contiguous counties, Guangxi | 207696 | 17415 | 496 | 251 | 0.51 (0.46-0.55) | 42.4 (38.8-46.1) | 1.61 (1.35-1.95) |
| Aggregated | 761784 | 23622 | 766 | 332 | 0.43 (0.40-0.47) | 13.4 (12.4-14.5) | 0.74 (0.64-0.84) |

**S15 Table. Estimated incidence rate of EV71-HFMD cases in the national surveillance database in the study areas of the EV71 vaccine phase III trials**.

### References

1. The World Bank. Life expectancy at birth [cited 2015 29 January]. Available from: <http://data.worldbank.org/indicator/SP.DYN.LE00.IN>.

2. Liu GG, Wu H, Li M, Gao C, Luo N. Chinese time trade-off values for EQ-5D health states. Value Health. 2014;17(5):597-604. doi: 10.1016/j.jval.2014.05.007. PubMed PMID: 25128053.

3. National Bureau of Statistics of China. National Data: National Accounts [cited 2015 29 January]. Available from: <http://data.stats.gov.cn/english/easyquery.htm?cn=C01>.

4. Zhu FC, Meng FY, Li JX, Li XL, Mao QY, Tao H, et al. Efficacy, safety, and immunology of an inactivated alum-adjuvant enterovirus 71 vaccine in children in China: a multicentre, randomised, double-blind, placebo-controlled, phase 3 trial. Lancet. 2013;381(9882):2024-32. doi: 10.1016/S0140-6736(13)61049-1. PubMed PMID: 23726161.

5. Zhu F, Xu W, Xia J, Liang Z, Liu Y, Zhang X, et al. Efficacy, safety, and immunogenicity of an enterovirus 71 vaccine in China. The New England journal of medicine. 2014;370(9):818-28. doi: 10.1056/NEJMoa1304923. PubMed PMID: 24571754.

6. Li R, Liu L, Mo Z, Wang X, Xia J, Liang Z, et al. An inactivated enterovirus 71 vaccine in healthy children. The New England journal of medicine. 2014;370(9):829-37. doi: 10.1056/NEJMoa1303224. PubMed PMID: 24571755.

7. Takahashi S, Liao Q, Van Boeckel TP, Xing W, Sun J, Hsiao VY, et al. Hand, foot, and mouth disease in China: modelling epidemic dynamics of enterovirus serotypes and implications for vaccination. PLOS Medicine. 2016. doi: 10.1371/journal.pmed.1001958.

8. Ang LW, Koh B, Chan KP, Chua LT, James L, Goh KT. Epidemiology and control of hand, foot and mouth disease in Singapore. Ann Acad Med Singapore. 2009;38:106-12.

9. Huang F-L, Chen C-H, Huang S-K, Chen P-Y. An outbreak of enterovirus 71 in a nursery. Scandinavian journal of infectious diseases. 2010;42(8):609-12.

10. Ma E, Wong S, Wong C, Chuang SK, Tsang T. Effects of public health interventions in reducing transmission of hand, foot, and mouth disease. The Pediatric infectious disease journal. 2011;30(5):432-5.

11. Ruan F, Yang T, Ma H, Jin Y, Song S, Fontaine RE, et al. Risk factors for hand, foot, and mouth disease and herpangina and the preventive effect of hand-washing. Pediatrics. 2011;127(4):e898-e904.
